# Synthesis, Antioxidant, and Antifungal Activities of β-Ionone Thiazolylhydrazone Derivatives and Their Application in Anti-Browning of Freshly Cut Potato

**DOI:** 10.3390/molecules28186713

**Published:** 2023-09-20

**Authors:** Cong Huang, Yuan Zhong, Rong Zeng, Jie Wang, Qingwen Fang, Shuzhen Xiao, Ji Zhang, Zongde Wang, Shangxing Chen, Dayong Peng

**Affiliations:** 1 College of Forestry, East China Woody Fragrance and Flavor Engineering Research Center of National Forestry and Grassland Administration, Jiangxi Agricultural University, Nanchang 330045, China; huangc5020@163.com (C.H.);; 2 College of Chemistry and Materials, Key Laboratory of Chemical Utilization of Plant Resources of Nanchang, East China Woody Fragrance and Flavor Engineering Research Center of National Forestry and Grassland Administration, Jiangxi Agricultural University, Nanchang 330045, China

**Keywords:** thiazolylhydrazone, synthesis, antioxidant, antifungal, anti-browning

## Abstract

In order to develop a new type of antioxidants with high efficiency, a series of β-ionone thiazolylhydrazone derivatives were designed and synthesized from β-ionone, and their structures were characterized by ^1^H-NMR, ^13^C-NMR, FT-IR, and HR-MS. The antioxidant test in vitro indicated that most of the target compounds had high biological activity. Among them, compound **1k** exhibited very strong DPPH (1,1-diphenyl-2-picrylhydrazyl radical)-scavenging activity with a half-maximal effective concentration (IC_50_) of 86.525 μM. Furthermore, in the ABTS (2,2-azinobis (3-ethylbenzothiazoline-6-sulfonate) diammonium salt)-scavenging experiment, compound **1m** exhibited excellent activity with an IC_50_ of 65.408 μM. Their biological activities were significantly better than those of the positive control Trolox. These two compounds, which have good free-radical-scavenging activity in vitro, were used as representative compounds in the anti-browning experiment of fresh-cut potatoes. The results showed that **1k** and **1m** had the same anti-browning ability as kojic acid, and they were effective browning inhibitors. In addition, it is well known that microbial infection is one of the reasons for food oxidation. Therefore, we investigated the antifungal activity of 25 target compounds against eight plant fungi at a concentration of 125 mg/L. The results indicated that these compounds all have some antifungal activity and may become new potential fungicides. Notably, compound 1u showed the best inhibitory effect against *Poria vaporaria*, with an inhibition rate as high as 77.71%; it is expected to become the dominant structure for the development of new antifungal agents.

## 1. Introduction

Microbial contamination and nutrient oxidation are considered to be the two most important causes of food deterioration in the process of food processing, storage, transportation, and marketing, which seriously affect human health and economic benefits [1]. It is particularly important to take some measures to prevent the oxidation of fruits and vegetables with short shelf-life and easy oxidation. The browning reaction is the most common form of fruit and vegetable oxidation, which is mainly divided into enzymatic browning and non-enzymatic browning [2]. The most intuitive effect of browning involves adverse changes in the sensory characteristics of fruits and vegetables, and some cases of browning will even produce toxic substances, which will directly affect the acceptance of consumers, resulting in serious economic losses. Therefore, preventing the oxidation of fruits and vegetables during storage or processing has become the primary concern of the food industry, and the development and application of antioxidants have naturally become a current research focus [3].

Ionone, a monoterpene found in natural products [4], is an important raw material widely used in the fields of medicine and perfume [5]. Experimental results have shown that the β-isomer of ionone has good biological activity [6,7]. In the field of science, the thiazole hydrazone skeleton is a key pharmacophore as it is widely present in compounds with various biological properties [8,9,10,11,12]. Petrou [13] reviewed 124 relevant studies in the past 5 years and found that many natural and synthetic thiazoles and their derivatives show remarkable biological activities, such as antioxidant, analgesic, antifungal, anticancer, antiallergic, antihypertensive, anti-inflammatory, antimalarial, antifungal, and antipsychotic [14,15,16]. Due to the significant biological activities of thiazolidines, scientists are actively involved in the design of new thiazolidine derivatives.

In recent years, there have been an increasing number of studies on antioxidants, and Kauthale [17] and Djafarou [18] have conducted systematic studies on the antioxidant properties of thiazolhydrazone compounds. What is different is that the scavenging activity of the compounds against ABTS free radicals was also studied in this paper, and the effect of introducing more types of functional groups on the antioxidant activity of the compounds was studied. In the study of natural compounds, the biological activity of antioxidants is often characterized by directly eliminating free radicals or providing reducing power to counteract the oxidative stress caused by free radicals. Lee [19] evaluated the antioxidant activity of about 700 plant extracts using the DPPH free-radical-scavenging assay. The antioxidant activity of thiazole hydrazone compounds can be demonstrated by their ability to scavenge DPPH and ABTS radicals [20,21], which is expected to provide assistance in the development of antioxidant drugs [22].

Previous research in our laboratory demonstrated that the six membered cy-clic monoterpenoid thiazole derivatives have good biological activity [23]. In this paper, 25 compounds containing the structure of thiazole hydrazone were designed and synthesized using β-ionone as the lead compound. The newly synthesized thiazole hydrazone derivatives with different substituent groups were characterized by ^1^H-NMR, ^13^C-NMR, FT-IR, and HRMS. All compounds were tested for free-radical scavenging and antifungal activity in vitro, and the potential of these compounds for food preservation in vivo was evaluated using fresh-cut potato as an example. The synthesis and bioactivity testing of these compounds can provide some reference for research on food antioxidants and antifungal drugs.

## 2. Results and Discussion

### 2.1. Synthesis and Characterization

**Scheme 1:**
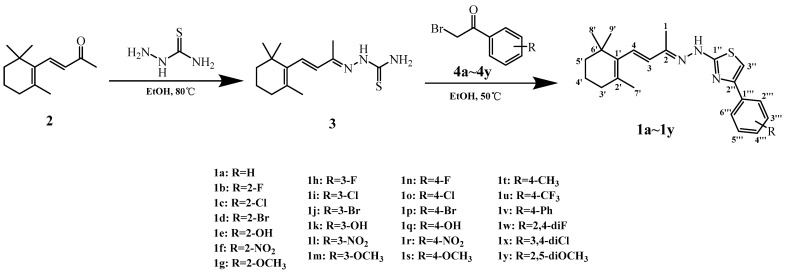
Synthesis of compounds **3** and **1a**~**1y**.

Compounds **1a**~**1y** were synthesized according to the multi-step synthetic route described in Scheme 1. Specifically, β-ionone (**2**) was condensed with thiosemicarbazide to form intermediate **3**. Then, intermediate **3** and 2-bromoacetophenone (**4a**~**4y**) were cyclized to synthesize thiazole hydrazone derivatives (**1a**~**1y**). 

All compounds were characterized by infrared spectroscopy in the range of 400–4000 cm^−1^. In compounds **1a**~**1y**, the peak near 3200 cm^−1^ was caused by N–H stretching vibrations. The absorption peak of the C=N bond appeared around 1600 cm^−1^. The absorption peak of the C–S bond appeared around 1100 cm^−1^. The stretching vibration of the aromatic ring (C–H) was observed at around 3000 cm^−1^. In addition, the aromatic ring could be further confirmed by the continuous peak packets appearing in the shape of fingers at 1400–1600 cm^−1^. In the ^1^H-NMR spectrum of compounds, the chemical shift of the proton on the imino group appeared at about 12.49 ppm, presenting as a single peak. The protons of the aromatic rings (Ar–H) appeared in the range of about 7.85–7.45 ppm. In the spectrum simulated by MestReNova 14 program, these aromatic protons also showed similar chemical shifts and shapes. In addition, the chemical shift of the proton on the thiazole ring appeared at about 6.80 ppm. Lastly, chemical shifts of protons on saturated carbon appeared in the range of 2.36–1.02 ppm. In the ^13^C-NMR spectrum of compounds, it was obvious that the carbon on the benzene ring appeared in the range of 120–160 ppm. The three carbons in the thiazole ring appeared at about 169 ppm, 140 ppm, and 125 ppm, and the carbon atoms in the methyl group appeared at about 20–30 ppm. The remaining carbon peaks appeared around 14–40 ppm. From the above characterization data, the correctness of the compound structure could be basically established. In addition, the measured values of HRMS (ESI) of all compounds were generally consistent with the predicted values.

### 2.2. Antioxidant Activity

Free-radical-scavenging tests such as DPPH and ABTS are widely used to evaluate the antioxidant capacity of compounds, and they have become simple, rapid, and low-cost methods. Through regression analysis, it was found that there was a linear relationship between the concentration of compounds and the radical-scavenging rate, and the IC_50_ value was calculated using the obtained linear regression equation, enabling a comparison of the strength of the free-radical-scavenging ability of each compound. Trolox is a vitamin E antioxidant with strong scavenging capacity against DPPH and ABTS radicals with IC_50_ values of 108.334 and 91.897 μM, respectively. The experimental results exhibited that compounds **1a**~**1y** had a good scavenging effect on the above two free radicals, and the correlation coefficients of the linear regression equations were all greater than 0.9, which indicated that their scavenging effect on free radicals would be enhanced with the increase in compound concentration. 

**Figure 1:**
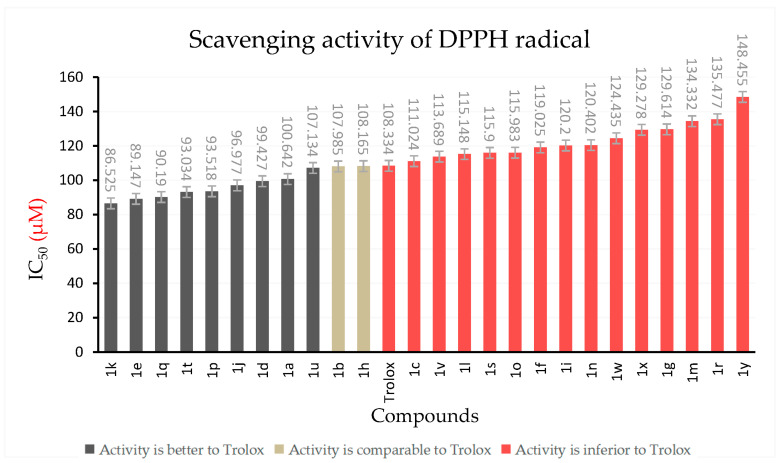
Scavenging activity of compounds **1a**~**1y** and Trolox against DPPH radicals.

As shown in Table 1 and Figure 1, eight compounds (**1a**, **1d**, **1e**, **1j**, **1k**, **1p**, **1q**, **1t**) showed significantly better DPPH radical-scavenging activity than the positive control Trolox. In contrast to compound **1a**, which contains no substituents, it was found that the introduction of functional groups such as hydroxyl, methyl, and halogen bromide favored DPPH scavenging. Among them, compound **1k** showed the greatest potential scavenging activity on DPPH free radicals with an IC_50_ value of 86.525 μM. The introduction of different halogen atoms also significantly changed the scavenging activity of target compounds on DPPH free radicals. It is worth noting that the introduction of bromine atoms enhanced the DPPH-scavenging ability of the compounds, while the introduction of fluorine atoms and chlorine atoms weakened the DPPH-scavenging ability, decreasing more obviously with the increase in fluorine atoms and chlorine atoms. This result may be due to the fact that fluorine atoms and chlorine atoms absorb more electricity than bromine atoms. The nitro group is a strong electron-withdrawing group, and its introduction weakened the scavenging ability of target compounds to DPPH, further proving that the introduction of electron-withdrawing groups weakened the scavenging ability of target compounds to DPPH free radicals. In addition, phenolic hydroxyl groups can react with DPPH free radicals [24]; therefore, compounds **1k**, **1e**, and **1q** containing phenolic hydroxyl groups had excellent DPPH free-radical-scavenging ability. However, when the hydroxyl group was replaced by methoxy group, the ability of the compound to scavenge DPPH radicals was greatly reduced [25].

**Figure 2:**
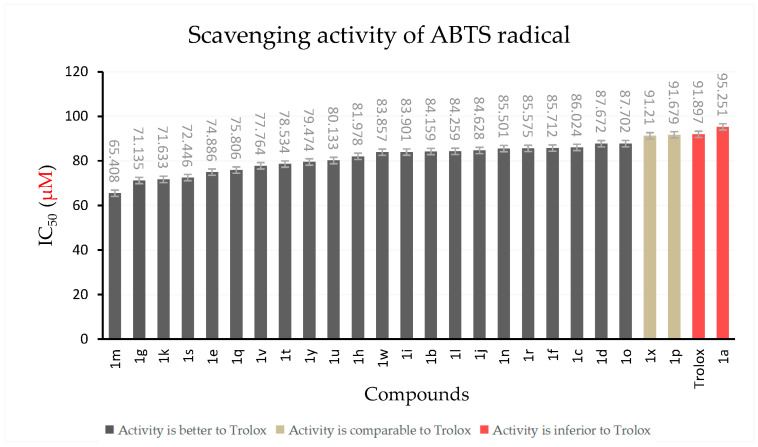
Scavenging activity of compounds **1a**~**1y** and Trolox against ABTS radical.

As shown in Table 2 and Figure 2, 22 compounds (except **1a**, **1p**, and **1x**) showed significantly better ABTS radical-scavenging activity than the positive control Trolox. Compared with compound **1a**, it was found that the introduction of substituents could significantly enhance the ability of the compounds to scavenge ABTS free radicals. Among them, compound **1m** had the strongest ABTS free-radical-scavenging activity, and its IC_50_ value was 65.408 μM. Compounds containing electron-donating groups (hydroxyl group, methyl group, and methoxy group) had a better ABTS radical-scavenging effect than other compounds, especially with regard to the methoxy group. In general, the effect of introducing halogen atoms on the ABTS radical-scavenging activity was slightly worse than that of other functional groups; however, when halogen atoms were introduced at the same position of the compound, compounds containing fluorine atoms were often the most active. In addition, it was found that, when the same functional group was introduced in the compound, introduction at the 3-position produced better ABTS free-radical-scavenging activity than at other positions.

The radical-scavenging activity of thiazole hydrazones may be related to the existence of N–H active groups in the hydrazone moiety, which has the ability to provide hydrogen atoms to eliminate free radicals [26]. The scavenging effect of these compounds on ABTS free radicals is better than that on DPPH free radicals, which may be due to the different scavenging mechanisms of the two radicals. The ABTS free-radical-scavenging mechanism of thiazole hydrazone compounds is based on hydrogen atom transfer (HAT), and the DPPH free radical-scavenging mechanism is based on hydrogen atom transfer (HAT) and single-electron transfer (SET) [24].

### 2.3. Anti-Browning Effect on Fresh-Cut Potatoes

**Figure 3:**
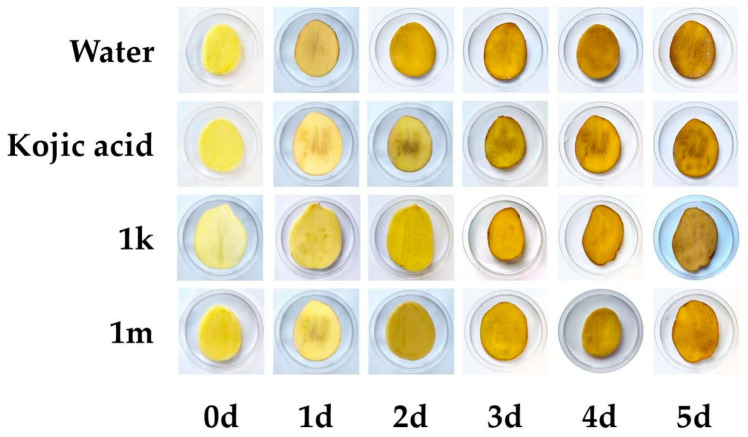
Visual observation of browning at 20 °C for 5 days.

**Figure 4:**
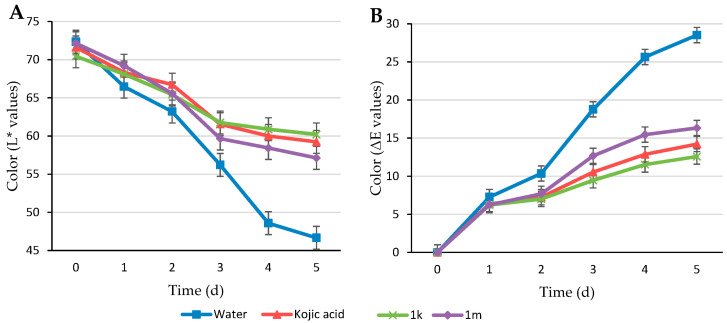
L* value changes (**A**); ΔE value changes (**B**).

In the process of food processing, the tissue damage of fresh fruits and vegetables leads to oxidative browning, and the degree of browning gradually deepens within a certain period. In order to verify whether these compounds have an anti-browning effect, compounds **1k** and **1m**, which showed good results in the free-radical-scavenging test in vitro, were selected for anti-browning experiments using fresh-cut potato slices. The experimental results showed that this effect was equivalent to that of the positive control kojic acid. It is evident from Figure 3 that the color of the potatoes gradually darkened with prolonged storage, indicating that browning occurred. In this study, L* and ΔE values were selected as key parameters to evaluate the anti-browning activity of compounds **1k** and **1m** on fresh-cut potato slices. As shown in Figure 4, the initial L* values of the experimental and control groups were statistically close or equal, both showing a downward trend with longer storage time. Compared with potato chips soaked in distilled water, kojic acid and other compounds added to the soaking solution had a significant effect on slowing the browning of potatoes. According to the degree of decrease in L* values, compounds **1k** and **1m** showed comparable browning resistance to kojic acid, with compound **1k** showing slightly stronger browning resistance than kojic acid. According to the ΔE value calculated by the formula, a similar conclusion was presented. Combined with the experimental data and visual sensory evaluation, it can be concluded that these two compounds had a certain anti-browning effect on fresh-cut fruits and vegetables. Compounds containing similar structures are of great interest in the development of fruit and vegetable preservatives.

### 2.4. Antifungal Activity

The antifungal activities of compounds **1a**~**1y** against eight fungi (*Sphaeropsis sapinea*, *Colletotrichum acutatum*, *Rhizoctonia solani AG1*, *Coriolus versicolor*, *Poria vaporaria*, *Phytophthora parasitica* var. *nicotianae*, *Fusarium oxysporum* f. sp. *niveum*, and *Fusarium verticillioides*) were evaluated at the concentration of 125 mg/L. These eight kinds of fungi are the most common plant pathogenic fungi affecting growth processes. The diameter of each colony was measured, and the inhibition rate was calculated. The broad-spectrum fungicide azoxystrobin was used as a positive control, and the results are shown in Table 3. All compounds had a certain antifungal activity. 

Importantly, the results obtained in our study suggested that compounds with fluorine-containing substituents (**1b**, **1h**, **1n**, **1u**, **1w**) possessed potent antifungal activities against most of the fungi, especially against *Poria vaporaria* fungi, all of which showed inhibition rates of more than 45%. It has been reported that fluorine atoms can effectively enhance the stability and biological activity of compounds in many aspects owing to their small atomic radius and strong electronegativity, thus improving the hydrophobicity and liposolubility of compounds [27,28,29,30]. Compounds containing nitro and methoxy groups (**1f**, **1g**, **1l**, **1m**, **1r**, **1s**, **1y**) usually had less effective antifungal effects on the eight kinds of fungi. In addition, the inhibition rate of target compounds **1n** and **1u** on *Poria vaporaria* was over 75%, which indicated that it had the potential to control tree rot. Therefore, compounds **1n** and **1u** could be used as a lead compound to control tree rot, which deserves further study. Our research also found that these compounds have relatively weak inhibitory effects on three fungi: *Coriolus versicolor*, *Fusarium oxysporum* f. sp. *niveum*, and *Fusarium verticillioides*.

## 3. Experimental

### 3.1. Materials and Methods

All chemicals were reagent-grade and without purification purchased from Xi-Long Co. (Shantou, China) and Aladdin. All plant fungi were provided by the Plant Pathology Laboratory of Agricultural College of Jiangxi Agricultural University. FT-IR spectra of the samples were performed between 400 and 4000 cm^−1^ from KBr pellets on. ^1^H-NMR and ^13^C-NMR spectra were carried out by AV 400 MHz spectrometer (CDCl_3_ as solvents). High-resolution mass spectrometry (HRMS) spectra were recorded on a triple time-of-flight TOF 5600+ (AB Sciex) mass spectrometer (Concord, ON, Canada). Absorbance was measured using a microplate reader (Multikan-FC). Compound melting points were determined using a melting point apparatus (44X-6T). The color change of potato samples was recorded using a colorimetric spectrophotometer (LS171, Linshang, Shenzhen, China).

### 3.2. Synthesis

#### 3.2.1. (*E*)-2-((*E*)-4-(2,6,6-Trimethylcyclohex-1-en-1-yl)but-3-en-2-ylidene)hydrazine-1-carbothioamide (**3**)

*β-Ionone* (**2**) (1 eq, 20 mmol, 3.85 g) and thiosemicarbazide (1.2 eq, 24 mmol, 2.19 g) were introduced in a flask and dissolved in ethanol; then, diluted hydrochloric acid (2.87 mol, 3 mL) was added and refluxed at 80 °C for 1 h. The reaction was monitored by TLC. When the reaction was completed, the obtained yellow solid was filtered [31]. It was recrystallized from 70% ethanol. After filtration and drying, compound **3** was obtained: m.p. 130–133 °C decomposition; yield: 86.84% (4.62 g); FT-IR *v* (cm^−1^): 3208 (*v* _N–H_), 3027 (*v* _C=C-H_), 2814 (*v* _C–H_), 1603 (*v* _C–N_), 1088 (*v* _C=S_) cm^−1^; ^1^H-NMR (400 MHz, CDCl_3_) δ 8.68 (s, ^1^H, NH), 6.61 (d, *J* = 16.55 Hz, ^1^H, NH), 6.44–6.32 (m, ^1^H, _4_-CH), 6.16–6.06 (m, ^1^H, _3_-CH), 2.03 (d, *J* = 2.15 Hz, 5H, _1_-CH_3_, _3′_-CH_2_), 1.71 (s, 3H, _7′_-CH_3_), 1.61 (d, *J* = 6.19 Hz, 2H, _4′_-CH_2_), 1.47 (dd, *J* = 2.34, 6.13 Hz, 2H, _5′_-CH_2_), 1.03 (s, 6H, _8′_-CH_3_, _9′_-CH_3_). ^13^C-NMR (101 MHz, CDCl_3_) δ 178.94 (C_1″_), 149.11 (C_2_), 136.69 (C_4_), 134.76 (C_1′_), 131.96 (C_2′_), 120.63 (C_3_), 39.59 (C_5′_), 34.19 (C_6′_), 33.16 (C_3′_), 28.88 (C_8′_, C_9′_), 21.67 (C_7′_), 19.07 (C_4′_), 11.13 (C_1_). HRMS calculated for C_14_H_23_N_3_S [M + H]^+^ 266.1613, found 266.1673.

#### 3.2.2. β-Ionone Thiazole Hydrazone Derivatives (**1a**~**1y**)

The synthesis methods were modified from Chen [31] and Babu [32]. Briefly, the above condensation product **3** (1 eq, 20 mmol, 5.32 g) was introduced in a flask and dissolved in ethanol. To this solution, 2-bromoacetophenone (1.2 eq, 24 mmol) was added, and the mixture was stirred at 50 °C. The reaction was monitored by TLC. When completion of the reaction was reached, it was recrystallized from 70% ethanol. After filtration and drying, compound β-ionone thiazole hydrazone derivatives (**1a**~**1y**) were obtained. All the obtained results indicated that compounds **1a**~**1y** were successfully synthesized in this study (see Supplementary Materials).

### 3.3. Antioxidant Activity

#### 3.3.1. DPPH Assay

The antioxidant activities of the compounds were measured by measuring the change in DPPH radical absorbance, referring to the methods in [33,34]. DPPH was weighed accurately, and 100 mL of an ethanol solution of DPPH (200 μM) was shaken and stored at room temperature in the dark. Then quantities of the compounds were weighed to prepare test solutions with different concentrations (200, 160, 120, 80, 40 μM). Then, 200 μL of DPPH–ethanol solution taken accurately was mixed with 50 μL of different concentrations of test solutions. After incubation in the dark for 30 min, the absorbance of the mixed solution was recorded at 517 nm wavelength as A_2_. In addition, 200 μL of DPPH solution and 50 μL of ethanol were mixed as blank, and the absorbance A_0_ was measured. Lastly, 200 μL of ethanol and 50 μL of the test solutions was mixed as a control, and the absorbance A_1_ was measured. The calculation formula used for the determination of the DPPH radical-scavenging rate was as follows:

$$\mathrm{DPPH}\;\mathrm{radical}\;\mathrm{scavenging}\;\mathrm{rate}=\left(1-\frac{A_{2}-A_{1}}{A_{0}}\right)\times 100\%$$

where A_0_ is the measured absorbance of ethanol and DPPH, A_1_ is the measured absorbance of test solution and ethanol, and A_2_ is the measured absorbance of test solution and DPPH. 

#### 3.3.2. ABTS Assay

The scavenging effect of the compounds on ABTS free radicals was determined, referring to the methods in [35,36,37]. ABTS solution (7 mmol) and K_2_S_2_O_8_ solution (2.45 mmol) were prepared with ultrapure water, mixed in equal volume, and placed at 4 °C in the dark for 24 h. The stock solution was valid for 2 days. The ABTS solution was diluted with ethanol to an absorbance of 0.7 ± 0.02 Abs at 734 nm. Then, quantities of the compounds were weighed to prepare test solutions with different concentrations (200, 160, 120, 80, 40 μM). A volume of 200 μL of ABTS solution was mixed with 50 μL of different concentrations of test solutions. After incubation in the dark for 10 min, the absorbance of the mixed solution was recorded at 734 nm. In addition, 200 μL of ABTS solution and 50 μL of ethanol were mixed as the blank, and the absorbance A_0_ was measured. Then, 200 μL of ethanol and 50 μL of the test solutions were mixed as a control, and the absorbance A_1_ was measured. The calculation formula used for determination of the ABTS radical\-scavenging rate was as follows:

$$\mathrm{ABTS}\;\mathrm{radical}\;\mathrm{scavenging}\;\mathrm{rate}=\left(1-\frac{A_{2}-A_{1}}{A_{0}}\right)\times 100\%$$

where A_0_ is the measured absorbance of ethanol and ABTS, A_1_ is the measured absorbance of test solution and ethanol, and A_2_ is the measured absorbance of test solution and ABTS. 

### 3.4. Anti-Browning Effect on Fresh-Cut Potatoes

Potatoes were purchased from local supermarkets and were of similar size, without damage or disease. The washed potatoes were cut into thin slices about 5 mm thick and completely immersed in the prepared solution (500 μM) for 5 min. They were drained and placed in sterile Petri dishes and stored at 20 °C. Distilled water and kojic acid were selected as control groups. The L* (lightness), a* (red-green), and b* (yellow-blue) values of potatoes at different times were measured using a colorimetric spectrophotometer [18,38]. To increase the confidence of the experimental results, all experiments were repeated several times. The total color difference (ΔE) was calculated as follows:

ΔE = [(L_t_* − L_initial_*)^2^ + (a_t_* − a_initial_*)^2^ + (b_t_* − b_initial_*)^2^]^0.5^

### 3.5. Antifungal Activity

In this research, eight plant fungi, namely, *Sphaeropsis sapinea*, *Colletotrichum acutatum*, *Rhizoctonia solani AG1*, *Coriolus versicolor*, *Poria vaporaria*, *Phytophthora parasitica* var. *nicotianae*, *Fusarium oxysporum* f. sp. *Niveum* and *Fusarium verticillioides* were used to determine the antifungal activity of the compounds according to the mycelial growth rate method. The compounds were dissolved in dimethyl sulfoxide to generate stock solutions (1.0 × 10^4^ mg/L), and then were further diluted to 125 mg/L on PDA plates. After PDA solidification, an inoculation loop was used to take 5 mm diameter fungus cakes from the edge of the 7-day-cultured pathogenic fungi colony. The fungus cake was moved to the center of the petri dishes, with the hyphae facing down; after the treatment, the petri dishes were placed in a constant-temperature biochemical incubator at 28 °C for cultivation, and the growth diameter of the hyphae was measured after 3–10 days. As a positive control, azoxystrobin (antifungal agent) was used, while the solution without the test compound was used as a blank control. All experiments were independently repeated three times [39,40]. 

## 4. Conclusions

In this paper, 25 new unreported thiazole hydrazone compounds were successfully synthesized using β-ionone as the main raw material and verified by a series of characterization data. In the in vitro antioxidant experiments, all target compounds showed a good ability to scavenge free radicals. Compared with the positive control Trolox, it was found that eight compounds were more effective in scavenging DPPH free radicals, and 22 compounds were more effective in scavenging ABTS free radicals. This result may be closely related to the structure of hydrazone in the target compounds. In the anti-browning experiment of freshly cut potatoes, compounds **1k** and **1m** showed similar anti-browning effects to kojic acid, and compound **1k** was even slightly more effective than kojic acid. This result further confirmed that the target compounds had excellent antioxidant activity. Although most compounds showed weaker effects than the positive control in antifungal experiments, there are few antifungal agents with this structure on the market. It is of great significance for the development of antifungal agents to further optimize this structure and improve the efficacy. To sum up, the excellent antioxidant properties of these target products are of great significance for developing more efficient antioxidants.

## Figures and Tables

**Table 1:** Scavenging activity of DPPH radicals.

Compound	R	DPPH	
		IC_50_ (μM)	r^2^
**1a**	H	100.642	0.970
**1b**	2-F	107.985	0.965
**1c**	2-Cl	111.024	0.952
**1d**	2-Br	99.427	0.947
**1e**	2-OH	89.147	0.942
**1f**	2-NO_2_	119.025	0.982
**1g**	2-OCH_3_	129.614	0.973
**1h**	3-F	108.165	0.997
**1i**	3-Cl	120.200	0.963
**1j**	3-Br	96.977	0.935
**1k**	3-OH	86.525	0.980
**1l**	3-NO_2_	115.148	0.966
**1m**	3-OCH_3_	134.332	0.963
**1n**	4-F	120.402	0.969
**1o**	4-Cl	115.983	0.973
**1p**	4-Br	93.518	0.917
**1q**	4-OH	90.190	0.972
**1r**	4-NO_2_	135.477	0.998
**1s**	4-OCH_3_	115.900	0.973
**1t**	4-CH_3_	93.034	0.989
**1u**	4-CF_3_	107.134	0.963
**1v**	4-Ph	113.689	0.925
**1w**	2,4-diF	124.435	0.990
**1x**	3,4-diCl	129.278	0.960
**1y**	2,5-diOCH_3_	148.455	0.967
**Trolox**	-	108.334	0.991

R, compound substituent; r^2^, correlation coefficient in linear regression equation.

**Table 2:** Scavenging activity of ABTS radical.

Compound	R	ABTS	
		IC_50_ (μM)	r^2^
**1a**	H	95.251	0.946
**1b**	2-F	84.159	0.984
**1c**	2-Cl	86.024	0.977
**1d**	2-Br	87.672	0.945
**1e**	2-OH	74.886	0.991
**1f**	2-NO_2_	85.712	0.990
**1g**	2-OCH_3_	71.135	0.959
**1h**	3-F	81.978	0.982
**1i**	3-Cl	83.901	0.957
**1j**	3-Br	84.628	0.987
**1k**	3-OH	71.633	0.966
**1l**	3-NO_2_	84.259	0.944
**1m**	3-OCH_3_	65.408	0.950
**1n**	4-F	85.501	0.943
**1o**	4-Cl	87.702	0.949
**1p**	4-Br	91.679	0.989
**1q**	4-OH	75.806	0.985
**1r**	4-NO_2_	85.575	0.965
**1s**	4-OCH_3_	72.446	0.949
**1t**	4-CH_3_	78.534	0.974
**1u**	4-CF_3_	80.133	0.978
**1v**	4-Ph	77.764	0.952
**1w**	2,4-diF	83.857	0.970
**1x**	3,4-diCl	91.210	0.956
**1y**	2,5-diOCH_3_	79.474	0.956
**Trolox**	-	91.897	0.958

R, compound substituent; r^2^, correlation coefficient in linear regression equation.

**Table 3:** Antifungal activity of compounds **1a**~**1y**.

Compound	R	Inhibition Rate (%)							
		S. S.	C. A.	R. S.	C. V.	P. V.	P. P.	F. N.	F. V.
**1a**	H	38.98	25.39	**63.13**	<10	**46.67**	40.19	14.22	22.58
**1b**	2-F	**46.25**	34.55	**61.75**	10.24	**45.42**	**45.45**	17.16	<10
**1c**	2-Cl	37.69	30.42	39.02	<10	**51.75**	41.04	<10	<10
**1d**	2-Br	27.62	23.81	37.77	11.64	**48.04**	37.91	<10	<10
**1e**	2-OH	34.87	21.99	41.15	<10	24.79	<10	<10	19.89
**1f**	2-NO_2_	27.30	20.03	31.90	<10	30.74	41.70	<10	<10
**1g**	2-OCH_3_	39.95	14.66	31.11	<10	35.42	22.73	11.76	21.77
**1h**	3-F	41.89	24.08	**51.24**	12.62	**46.25**	**45.08**	20.34	18.28
**1i**	3-Cl	37.00	29.02	40.55	<10	**57.75**	44.60	13.45	<10
**1j**	3-Br	29.30	19.37	21.66	<10	43.13	32.78	<10	<10
**1k**	3-OH	36.02	24.35	40.46	12.10	30.37	<10	<10	17.53
**1l**	3-NO_2_	23.97	24.87	21.20	<10	29.58	39.23	<10	<10
**1m**	3-OCH_3_	31.23	29.84	17.51	<10	41.25	16.51	10.29	11.29
**1n**	4-F	**49.64**	30.10	**59.45**	12.14	**76.25**	42.11	13.24	28.23
**1o**	4-Cl	35.35	24.08	41.24	10.48	**64.17**	40.19	<10	<10
**1p**	4-Br	27.60	25.39	41.94	<10	**49.17**	42.34	<10	<10
**1q**	4-OH	33.50	27.64	44.05	<10	25.80	<10	<10	20.48
**1r**	4-NO_2_	31.96	13.87	42.86	<10	20.83	40.91	<10	<10
**1s**	4-OCH_3_	29.30	39.01	31.34	<10	35.42	19.14	<10	11.02
**1t**	4-CH_3_	26.39	28.27	**55.07**	<10	**61.67**	23.92	<10	<10
**1u**	4-CF_3_	**46.25**	33.77	**64.29**	<10	77.71	**51.20**	<10	<10
**1v**	4-Ph	19.61	<10	18.43	<10	29.17	16.30	<10	<10
**1w**	2,4-diF	**58.60**	34.55	**74.42**	12.62	**62.29**	**52.15**	21.33	<10
**1x**	3,4-diCl	27.85	23.04	<10	10.24	**45.83**	39.47	<10	<10
**1y**	2,5-diOCH_3_	30.75	20.42	<10	<10	26.30	<10	<10	<10
**AZO**	-	50.52	58.64	67.84	51.43	84.79	45.93	54.90	49.60

Data are given as the mean of triplicate experiments. An inhibition rate >45% indicates good antifungal activity, as shown in bold. S. S., *Sphaeropsis sapinea*; C. A., *Colletotrichum acutatum*; R. S., *Rhizoctonia solani AG1*; C. V., *Coriolus versicolor*; P. V., *Poria vaporaria*; P. P., *Phytophthora parasitica* var. *nicotianae*; F. N., *Fusarium oxysporum* f. sp. *niveum*; F. V., *Fusarium verticillioides.* AZO, azoxystrobin.

## Data Availability

Data are contained within the manuscript.

## Supplementary Materials

The following supporting information can be downloaded at: https://www.mdpi.com/article/10.3390/molecules28186713/s1.
