# Supplementary material for: Synthesis, Antioxidant, and Antifungal Activities of β-Ionone Thiazolylhydrazone Derivatives and Their Application in Anti-Browning of Freshly Cut Potato
Source: Molecules. 2023 Sep 20;28(18):6713. doi: 10.3390/molecules28186713 (PMC10537339; doi:10.3390/molecules28186713)
Supplement: Supplementary file 1 [file molecules-28-06713-s001.zip › molecules-2576282-supplementary.pdf]

Supporting Information

Synthesis, Antioxidant, and Antifungal Activities of β-Ionone Thiazolylhydrazone Derivatives and Their Application in Anti-Browning of Freshly Cut Potato

Cong Huang [1], Yuan Zhong [1], Rong Zeng [1], Jie Wang [2], Qingwen Fang [1], Shuzhen Xiao [2], Ji Zhang [1], Zongde Wang [1,2], Shangxing Chen [1,*] and Dayong Peng [2,*]

[1] College of Forestry, East China Woody Fragrance and Flavor Engineering Research Center of National Forestry and Grassland Administration, Jiangxi Agricultural University, Nanchang 330045, China; huangc5020@163.com (C.H.)

[2] College of Chemistry and Materials, Key Laboratory of Chemical Utilization of Plant Resources of Nanchang, East China Woody Fragrance and Flavor Engineering Research Center of National Forestry and Grassland Administration, Jiangxi Agricultural University, Nanchang 330045, China

* Correspondence: csxing@126.com (S.C.); dayongpeng@163.com (D.P.)

Content

1. Structural characterization data of compounds 1a~1y.................................... 3

2. Spectrums of compounds 3 and 1a~1y... 14

1.Structural characterization data of compounds 1a~1y.

4-phenyl-2-(2-((2E,3E)-4-(2,6,6-trimethylcyclohex-1-en-1-yl)but-3-en-2-ylidene)hydrazinyl)thiazole(**1a**)

Pink solid, Yield 62.33%, FT-IR *v* (cm^{-1}): 3230 (v_{N-H}), 3096, 3084 ($v_{C=C-H}$), 2956, 2923, 2891, 2857, 2818 (v_{C-H}), 1614 ($v_{C=N}$), 1587, 1493, 1360 ($v_{Benzene\ ring}$), 1094 (v_{C-S-C}). ^{1}H NMR (400 MHz, $CDCl_3$) δ 12.47 (s, 1H, NH), 7.72 – 7.69 (m, 2H, $_{2''''}$-CH, $_{6''''}$-CH), 7.45 (d, *J* = 7.48 Hz, 3H, $_{3''''}$-CH, $_{4''''}$-CH, $_{5''''}$-CH), 6.74 (d, *J* = 23.45 Hz, 2H, $_4$-CH, $_{3''}$-CH), 6.21 (d, *J* = 16.49 Hz, 1H, $_3$-CH), 2.30 (s, 3H, $_{13}$-CH_3), 2.03 (s, 2H, $_{3'}$-CH_2), 1.73 (s, 3H, $_{7'}$-CH_3), 1.60 (d, *J* = 6.14 Hz, 2H, $_{4'}$-CH_2), 1.47 (d, *J* = 6.12 Hz, 2H, $_{5'}$-CH_2), 1.04 (s, 6H, $_{8'}$-CH_3, $_{9'}$-CH_3). ^{13}C NMR (101 MHz, $CDCl_3$) δ 169.18(C-$_{1''}$), 157.45(C-$_2$), 140.58(C-$_{2''}$), 136.66(C-$_4$), 132.93(C-$_{1'}$), 130.74(C-$_{1''''}$), 130.38(C-$_{2'}$), 129.58(C-$_{3''''}$, C-$_{5''''}$), 127.23(C-$_{4''''}$), 125.57(C-$_{2''''}$, C-$_{6''''}$), 101.97(C-$_{3''}$), 101.16(C-$_3$), 39.49(C-$_{5'}$), 34.17(C-$_{3'}$), 33.20(C-$_{6'}$), 28.89（C-$_{8'}$, C-$_{9'}$), 21.78（C-$_{7'}$), 19.00（C-$_{4'}$), 13.79（C-$_1$). HRMS calculated for $C_{22}H_{27}N_3S$ $[M+H]^+$ 366.2004, found 366.1993.

4-(2-fluorophenyl)-2-(2-((2E,3E)-4-(2,6,6-trimethylcyclohex-1-en-1-yl)but-3-en-2-ylidene)hydrazinyl)thiazole(**1b**)

Yellow solid, Yield 59.33%, FT-IR *v* (cm^{-1}): 3236 (v_{N-H}), 3102 ($v_{C=C-H}$), 2926, 2861, 2826 (v_{C-H}), 1618 ($v_{C=N}$), 1503, 1459, 1361 ($v_{Benzene\ ring}$), 1093 (v_{C-S-C}). ^{1}H NMR (400 MHz, $CDCl_3$) δ 12.50 (s, 1H, NH), 7.88 (d, *J* = 1.63 Hz, 1H, $_{6''''}$-CH), 7.43 – 7.28 (m, 2H, $_{4''''}$-CH, $_{3''''}$-CH), 7.21 – 7.13 (m, 1H, $_{5''''}$-CH), 7.04 (s, 1H, $_{3''}$-CH), 6.73 (d, *J* = 16.50 Hz, 1H, $_4$-CH), 6.20 (d, *J* = 16.51 Hz, 1H, $_3$-CH), 2.30 (s, 3H, $_{13}$-CH_3), 2.02 (s, 2H, $_{3'}$-CH_2), 1.72 (s, 3H, $_{7'}$-CH_3), 1.63 – 1.57 (m, 2H, $_{4'}$-CH_2), 1.46 (d, *J* = 6.06 Hz, 2H, $_{5'}$-CH_2), 1.03 (s, 6H, $_{8'}$-CH_3, $_{9'}$-CH_3). ^{13}C NMR (101 MHz, DMSO) δ 168.65(C-$_{1''}$), 153.90(C-F, $^{2'''}J_{C-F}$=284.4Hz), 137.04(C-$_{2''}$), 133.36(C-F, $^{4'''}J_{C-F}$=1.8Hz), 132.41(C-$_{2'}$), 131.39(C-F, $^{1'''}J_{C-F}$=7.2Hz), 130.29(C-$_2$), 127.81(C-$_{5''''}$), 119.80(C-$_{1'}$), 117.52(C-F, $^{3'''}J_{C-F}$=26.2Hz), 117.26(C-$_{3''}$), 104.81(C-$_3$), 39.55(C-$_{5'}$), 34.28(C-$_{3'}$), 33.02(C-$_{6'}$), 29.17(C-$_{8'}$, C-$_{9'}$), 21.92(C-$_{7'}$), 19.10(C-$_{4'}$), 13.10(C-$_1$). HRMS calculated for $C_{22}H_{26}FN_3S$ $[M+H]^+$ 384.1910, found 384.1919.

4-(2-chlorophenyl)-2-(2-((2E,3E)-4-(2,6,6-trimethylcyclohex-1-en-1-yl)but-3-en-2-ylidene)hydrazinyl)thiazole(**1c**)

White solid, Yield 63.66%, FT-IR *v* (cm^{-1}): 3176 (ν_{N-H}), 3104, 3055 ($\nu_{C=C-H}$), 2958, 2935 (ν_{C-H}), 1607 ($\nu_{C=N}$), 1485, 1453, 1356 ($\nu_{Benzene\ ring}$), 1080(ν_{C-S-C}). ^1H NMR (400 MHz, $CDCl_3$) δ 12.48 (s, 1H, NH), 7.80 (d, J = 1.89 Hz, 1H, 6''''-CH), 7.71 (dd, J = 7.33, 2.27 Hz, 1H, 4''''-CH), 7.57 – 7.52 (m, 1H, 2''''-CH), 7.37 (t, J = 7.96 Hz, 1H, 5''''-CH), 6.82 – 6.71 (m, 2H, 3''-CH, 4-CH), 6.21 (d, J = 16.42 Hz, 1H, 3-CH), 2.32 (s, 3H, 13-CH_3), 2.05 (s, 2H, 3'-CH_2), 1.75 (s, 3H, 7'-CH_3), 1.62 (d, J = 6.19 Hz, 2H, 4'-CH_2), 1.51 – 1.47 (m, 2H, 5'-CH_2), 1.06 (s, 6H, 8'-CH_3, 9'-CH_3). ^{13}C NMR (101 MHz, $CDCl_3$) δ 169.34(C-1''), 157.89(C-2), 139.42(C-2''), 136.97(C-4), 136.61(C-1'), 135.57(C-6'''), 133.07(C-1'''), 131.11(C-4'''), 130.65(C-5'''), 130.51(C-3''), 128.99(C-2'''), 125.68(C-3'''), 123.87(C-2'), 102.22(C-3), 39.57(C-5'), 34.21(C-3'), 33.24(C-6'), 28.90(C-8', C-9'), 21.75(C-7'), 19.03(C-4'), 13.86(C-1). HRMS calculated for $C_{22}H_{26}ClN_3S$ $[M+H]^+$ 400.1614, found 400.1672.

4-(2-bromophenyl)-2-(2-((2E,3E)-4-(2,6,6-trimethylcyclohex-1-en-1-yl)but-3-en-2-ylidene)hydrazinyl)thiazole(**1d**)

White solid, Yield 49.95%, FT-IR *v* (cm^{-1}): 3127 (ν_{N-H}), 3060 ($\nu_{C=C-H}$), 2959, 2927, 2862 (ν_{C-H}), 1555 ($\nu_{C=N}$), 1455, 1430, 1359 ($\nu_{Benzene\ ring}$), 1072(ν_{C-S-C}). ^1H NMR (400 MHz, $CDCl_3$) δ 12.48 (s, 1H, NH), 7.82 – 7.69 (m, 2H, 6''''-CH, 4''''-CH), 7.57 – 7.53 (m, 1H, 2''''-CH), 7.37 (t, *J* = 7.94 Hz, 1H, 5''''-CH), 6.77 (d, *J* = 30.26 Hz, 2H, 3''-CH, 4-CH), 6.21 (d, *J* = 16.50 Hz, 1H, 3-CH), 2.31 (s, 3H, 13-CH_3), 2.04 (t, *J* = 6.32 Hz, 2H, 3'-CH_2), 1.74 (s, 3H, 7'-CH_3), 1.66 – 1.57 (m, 2H, 4'-CH_2), 1.50 – 1.44 (m, 2H, 5'-CH_2), 1.05 (s, 6H, 8'-CH_3, 9'-CH_3). HRMS calculated for $C_{22}H_{26}BrN_3S$ $[M+H]^+$ 444.1109, found 444.1155.

2-(2-(2-((2E,3E)-4-(2,6,6-trimethylcyclohex-1-en-1-yl)but-3-en-2-ylidene)hydraziney l)thiazol-4-yl)phenol(**1e**)

Yellow solid, Yield 66.72%, FT-IR *v* (cm^{-1}):3473 (ν_{O-H}), 3403 (ν_{N-H}), 3149, 3046 ($\nu_{C=C-H}$), 2943, 2865 (ν_{C-H}), 1604 ($\nu_{C=N}$), 1488.13, 1455, 1358 ($\nu_{Benzene\ ring}$), 1094(ν_{C-S-C}).^1H NMR (400 MHz, $CDCl_3$) δ ^1H NMR (400 MHz, $CDCl_3$) δ 12.71 (s, 1H, NH), 7.52 (dd, *J* = 1.63, 7.95 Hz, 1H, 6''''-CH), 7.25 – 7.19 (m, 1H, 4''''-CH), 7.06 (d, *J* = 8.15 Hz, 1H, 3''-CH), 6.96 (t, *J* = 7.55 Hz, 1H, 5''''-CH), 6.83 (s, 1H, 3''''-CH), 6.73 (d, *J* = 16.59 Hz, 1H, 4-CH), 6.22 (d, *J* = 16.48 Hz, 1H, 3-CH), 2.36 (s, 3H, 13-CH_3), 2.05 (s, 2H, 3'-CH_2), 1.63 (dd, *J* = 2.66, 6.15 Hz, 3H, 7'-CH_3), 1.51 – 1.46 (m, 2H, 4'-CH_2), 1.25

(s, 2H, 5'-CH_2), 1.06 (s, 6H, 8'-CH_3, 9'-CH_3). ^{13}C NMR (101 MHz, $CDCl_3$) δ 168.68(C-1''), 160.93(C-6'''), 158.42(C-2), 157.58(C-2''), 136.72(C-4), 134.42(C-1'), 132.94(C-4'''), 131.71(C-2'), 130.70(C-2'''), 127.73(C-5'''), 125.47(C-1'''), 116.55(C-3'''), 115.61(C-3''), 105.94(C-3), 39.51(C-5'), 34.16(C-3'), 33.19(C-6'), 28.87(C-8', C-9'), 21.74(C-7'), 18.98(C-4'), 13.82(C-1). HRMS calculated for $C_{22}H_{27}N_3OS$ $[M+H]^+$ 382.1953, found 382.1931.

4-(2-nitrophenyl)-2-(2-((2E,3E)-4-(2,6,6-trimethylcyclohex-1-en-1-yl)but-3-en-2-ylidene)hydrazinyl)thiazole(**1f**)

Yellow solid, Yield 39.44%, FT-IR *ν* (cm^{-1}): 3232 ($ν_{N-H}$), 3093, 2956 ($ν_{C=C-H}$), 2956, 2924, 2896, 2858, 2818($ν_{C-H}$), 1618 ($ν_{C=N}$), 1507, 1485, 1365 ($ν_{Benzene\ ring}$), 1090($ν_{C-S-C}$). ^1H NMR (400 MHz, $CDCl_3$) δ 8.53 (d, *J* = 2.02 Hz, 1H, 2'''-CH), 8.19 – 8.08 (m, 2H, 6'''-CH, 4'''-CH), 7.55 (m, 1H, 5'''-CH), 6.99 (s, 1H, 3''-CH), 6.62 (d, *J* = 15.54 Hz, 1H, 4-CH), 6.21 (d, *J* = 15.92 Hz, 1H, 3-CH), 2.16 (s, 3H, 13-CH_3), 2.02 (s, 2H, 3'-CH_2), 1.73 (s, 3H, 7'-CH_3), 1.64 – 1.55 (m, 2H, 4'-CH_2), 1.49 – 1.42 (m, 2H, 5'-CH_2), 1.03 (s, 6H, 8'-CH_3, 9'-CH_3). ^{13}C NMR (101 MHz, $CDCl_3$) δ 169.22(C-1''), 157.79(C-2), 141.98(C-2''), 139.53(C-4), 136.93(C-1'), 136.55(C-4'''), 133.06(C-1'''), 130.63(C-2'), 129.87(C-3''', C-5'''), 126.87(C-2''', C-6'''), 125.71(C-3''), 101.53(C-3), 39.52(C-5'), 34.19(C-3'), 33.22(C-6'), 28.88(C-8', C-9'), 21.76(C-7'), 19.00(C-4'), 13.78(C-1). HRMS calculated for $C_{22}H_{26}N_4O_2S$ $[M+H]^+$ 411.1855, found 411.1841.

4-(2-methoxyphenyl)-2-(2-((2E,3E)-4-(2,6,6-trimethylcyclohex-1-en-1-yl)but-3-en-2-ylidene)hydrazineyl)thiazole(**1g**)

White solid, Yield 71.82%, FT-IR *ν* (cm^{-1}): 3196($ν_{N-H}$), 3121($ν_{C=C-H}$), 2938, 2772 ($ν_{C-H}$), 1599 ($ν_{C=N}$), 1572, 1462 ($ν_{Benzene\ ring}$), 1280 ($ν_{Ph-O-C}$), 1083 ($ν_{C-S-C}$). ^1H NMR (400 MHz, $CDCl_3$) δ 12.99 (s, 1H, NH), 7.61 – 7.58 (m, 1H, 6'''-CH), 7.38 (d, *J* = 1.69 Hz, 1H, 4'''-CH), 7.04 – 6.99 (m, 2H, 3'''-CH, 5'''-CH), 6.88 (s, 1H, 3''-CH), 6.70 (d, *J* = 16.45 Hz, 1H, 4-CH), 6.19 (d, *J* = 16.46 Hz, 1H, 3-CH), 4.07 (s, 3H, OCH_3), 2.34 (s, 3H, 13-CH_3), 2.02 (t, *J* = 6.36 Hz, 2H, 3'-CH_2), 1.72 (s, 3H, 7'-CH_3), 1.62 – 1.57 (m, 2H, 4'-CH2), 1.47 – 1.44 (m, 2H, 5'-CH_2), 1.03 (s, 6H, 8'-CH_3, 9'-CH_3). ^{13}C NMR (101 MHz, $CDCl_3$) δ 168.52(C-1''), 157.40(C-6'''), 156.11(C-2), 137.95(C-2''), 136.59(C-4), 136.28(C-1'), 132.61(C-2'''), 131.68(C-2'), 130.98(C-4'''), 128.07(C-5'''), 121.29(C-1'''),

115.87(C-3'''), 111.77(C-3''), 103.08(C-3), 56.18(C-7'''), 39.48(C-5'), 34.16(C-3'), 33.16(C-6'), 28.87(C-8', C-9'), 21.74(C-7'), 19.00(C-4'), 14.42(C-1). HRMS calculated for $C_{23}H_{29}N_3OS$ $[M+H]^+$ 396.2110, found 396.2105.

4-(3-fluorophenyl)-2-((E)-2-((3E)-4-(2,6,6-trimethylcyclohex-1-en-1-yl)but-3-en-2-yl idene)hydrazin-1-yl)-1,3-thiazole(**1h**)

Yellow solid, Yield 70.81%, FT-IR *v* (cm^{-1}): 3232 (ν_{N-H}), 3093, 2956 ($\nu_{C=C-H}$), 2956, 2924, 2896, 2858, 2818(ν_{C-H}), 1618 ($\nu_{C=N}$), 1507, 1485, 1365 ($\nu_{Benzene\ ring}$), 1090(ν_{C-S-C}). ^1H NMR (400 MHz, $CDCl_3$) δ 12.52 (s, 1H, NH), 7.57 (d, *J* = 7.85 Hz, 1H, 6'''-CH), 7.51 – 7.45 (m, 1H, 5'''-CH), 7.39 (d, *J* = 9.26 Hz, 1H, 2'''-CH), 7.14 (d, *J* = 2.44 Hz, 1H, 3''-CH), 6.79 (s, 2H, 4'''-CH, 4-CH), 6.22 (d, *J* = 16.51 Hz, 1H, 3-CH), 2.32 (s, 3H, 13-CH_3), 2.05 (s, 2H, 3'-CH_2), 1.74 (s, 3H, 7'-CH_3), 1.62 (d, *J* = 6.18 Hz, 2H, 4'-CH2), 1.50 – 1.46 (m, 2H, 5'-CH_2), 1.05 (s, 6H, 8'-CH_3, 9'-CH_3). ^{13}C NMR (101 MHz, $CDCl_3$) δ 169.29(C-1''), 163.06(C-F, $^{3'''}J_{C-F}$=247.2Hz), 157.88(C-2), 139.47(C-4), 136.77 (C-F, $^{2'''}J_{C-F}$=37.2Hz), 133.07(C-2'), 131.60(C-F, $^{5'''}J_{C-F}$=8.4Hz), 130.66(C-2''), 129.20(C-F, $^{6'''}J_{C-F}$=8.0Hz), 121.47(C-3''), 117.66 (C-F, $^{2'''}J_{C-F}$=20.8Hz), 112.71(C-F, $^{4'''}J_{C-F}$=23.8Hz), 102.27(C-3), 39.55(C-5'), 34.21(C-3'), 33.24(C-6'), 28.91(C-8', C-9'), 21.77(C-7'), 19.02(C-4'), 13.85(C-1). HRMS calculated for $C_{22}H_{26}FN_3S$ $[M+H]^+$ 384.1910, found 384.1905.

4-(3-chlorophenyl)-2-(2-((2E,3E)-4-(2,6,6-trimethylcyclohex-1-en-1-yl)but-3-en-2-yli dene)hydrazinyl)thiazole(**1i**)

Yellow solid, Yield 67.43%, FT-IR *v* (cm^{-1}): 3243 (ν_{N-H}), 3118, 3064($\nu_{C=C-H}$), 2932, 2905, 2864(ν_{C-H}), 1622($\nu_{C=N}$), 1587, 1505, 1362($\nu_{Benzene\ ring}$), 1097(ν_{C-S-C}). ^1H NMR (400 MHz, $CDCl_3$) δ 12.44 (s, 1H, NH), 7.66 (d, J = 8.59 Hz, 2H, 6'''-CH, 4'''-CH), 7.45 (d, J = 8.59 Hz, 2H, 5'''-CH, 2'''-CH), 6.82 – 6.69 (m, 2H, 3''-CH, 4-CH), 6.22 (d, J = 16.55 Hz, 1H, 1H, 3-CH), 2.31 (s, 3H, 13-CH_3), 2.10 – 2.01 (m, 2H, 3'-CH_2), 1.74 (s, 3H, 7'-CH_3), 1.67 – 1.57 (m, 2H, 4'-CH_2), 1.52 – 1.45 (m, 2H, 5'-CH_2), 1.05 (s, 6H, 8'-CH_3, 9'-CH_3). ^{13}C NMR (101 MHz, $CDCl_3$) δ 169.22(C-1''), 157.79(C-2), 139.53(C-2''), 136.92(C-4), 136.55(C-1'), 133.06(C-1'''), 130.63(C-2'), 129.87(C-4''', C-2'''), 126.87(C-3'''), 125.71(C-2'), 101.53(C-3''), 39.52(C-5'), 34.19(C-3'), 33.22(C-6'), 28.88(C-8', C-9'), 21.76(C-7'), 19.00(C-4'), 13.78(C-1). HRMS calculated for $C_{22}H_{26}ClN_3S$ $[M+H]^+$ 400.1614, found 400.1667.

4-(3-bromophenyl)-2-((E)-2-((3E)-4-(2,6,6-trimethylcyclohex-1-en-1-yl)but-3-en-2-yl

idene)hydrazin-1-yl)-1,3-thiazole(**1j**)

Brown solid, Yield 67.55%, FT-IR *v* (cm^{-1}): 3241(v_{N-H}), 3116, 3064($v_{C=C-H}$), 2940, 2902, 2863, 2712 (v_{C-H}), 1623($v_{C=N}$), 1587, 1504, 1284($v_{Benzene\ ring}$), 1094 (v_{C-S-C}). ^1H NMR (400 MHz, $CDCl_3$) δ 12.49 (s, 1H, NH), 7.81 (d, J = 1.89 Hz, 1H, 6'''-CH), 7.73 (d, J = 9.60 Hz, 1H, 4'''-CH), 7.56 (d, J = 8.08 Hz, 1H, 2'''-CH), 7.38 (t, J = 7.96 Hz, 1H, 5'''-CH), 6.78 (d, J = 30.32 Hz, 2H, 3''-CH, 4-CH), 6.23 (d, J = 16.42 Hz, 1H, 3-CH), 2.32 (s, 3H, 13-CH_3), 2.05 (s, 2H, 3'-CH_2), 1.75 (s, 3H, 7'-CH_3), 1.62 (d, J = 6.19 Hz, 2H, 4'-CH_2), 1.51 – 1.47 (m, 2H, 5'-CH_2), 1.06 (s, 6H, 8'-CH_3, 9'-CH_3). ^{13}C NMR (101 MHz, $CDCl_3$) δ 169.22(C-1''), 157.83(C-2), 139.06(C-2''), 136.94(C-4), 136.54(C-1'), 133.38(C-1'''), 133.08(C-5'''), 131.28(C-6'''), 130.62(C-2'), 129.13(C-4'''), 128.50(C-2'''), 124.25(C-3'''), 123.47(C-3''), 102.42(C-3), 39.50(C-5'), 34.18(C-3'), 33.22(C-6'), 28.89(C-8', C-9'), 21.78(C-7'), 18.99(C-4'), 13.82(C-1). HRMS calculated for $C_{22}H_{26}BrN_3S$ $[M+2H]^+$ 445.1187, found 445.1155.

3-(2-(2-((2E,3E)-4-(2,6,6-trimethylcyclohex-1-en-1-yl)but-3-en-2-ylidene)hydrazinyl) thiazol-4-yl)phenol(**1k**)

Yellow solid, Yield 44.80%, FT-IR *v* (cm^{-1}):3229 (v_{O-H}), 3176 (v_{N-H}), 3104, 3055 ($v_{C=C-H}$), 2958, 2935 (v_{C-H}), 1607 ($v_{C=N}$), 1485, 1453, 1356 ($v_{Benzene\ ring}$), 1080(v_{C-S-C}). ^1H NMR (400 MHz, $CDCl_3$) δ 12.16 (s, 1H, NH), 7.29 (t, J = 7.93 Hz, 2H, 6'''-CH, 4'''-CH), 7.17 (dt, J = 7.78, 1.30 Hz, 1H, 3''-CH), 6.98 – 6.93 (m, 1H, 5'''-CH), 6.80 – 6.68 (m, 2H, 2'''-CH, 4-CH), 6.24 (d, J = 16.48 Hz, 1H, 3-CH), 2.31 (s, 3H, 13-CH_3), 2.06 (t, J = 6.56 Hz, 2H, 3'-CH_2), 1.80 – 1.70 (m, 3H, 7'-CH_3), 1.66 – 1.60 (m, 2H, 4'-CH_2), 1.52 – 1.47 (m, 2H, 5'-CH_2), 1.07 (s, 6H, 8'-CH_3, 9'-CH_3). ^{13}C NMR (101 MHz, $CDCl_3$) δ 168.96(C-1''), 157.57(C-6'''), 157.34(C-2), 140.47(C-2''), 136.85(C-4), 136.61(C-1'), 133.04(C-4'''), 130.93(C-2'), 130.69(C-2'''), 128.08(C-5'''), 118.07(C-1'''), 117.17(C-3'''), 112.37(C-3''), 101.13(C-3), 39.57(C-5'), 34.22(C-3'), 33.24(C-6'), 28.91(C-8', C-9'), 21.76(C-7'), 19.03(C-4'), 13.63(C-1). HRMS calculated for $C_{22}H_{27}N_3OS$ $[M+H]^+$ 382.1953, found 382.1899.

4-(3-nitrophenyl)-2-((E)-2-((3E)-4-(2,6,6-trimethylcyclohex-1-en-1-yl)but-3-en-2-ylidene)hydrazin-1-yl)-1,3-thiazole(**1l**)

Yellow solid, Yield 51.44%, FT-IR *v* (cm^{-1}): 3437(v_{N-H}), 3208, 3112 ($v_{C=C-H}$), 3067, 2923, 2860 (v_{C-H}), 1609($v_{C=N}$), 1560 (Ph-NO_2), 1529, 1453, 1343 ($v_{Benzene\ ring}$), 1096(v_{C-S-C}). ^1H NMR (400 MHz, $CDCl_3$) δ 8.53 (d, *J* = 2.06 Hz, 1H, 2'''-CH), 8.18 –

8.09 (m, 2H, 6'''-CH, 4'''-CH), 7.62 – 7.57 (m, 1H, 5'''-CH), 6.99 (s, 1H, 3''-CH), 6.62 (d, *J* = 16.47 Hz, 1H, 4-CH), 6.21 (d, *J* = 16.48 Hz, 1H, 3-CH), 2.16 (s, 3H, 13-CH_3), 2.03 (s, 2H, 3'-CH_2), 1.73 (s, 3H, 7'-CH_3), 1.60 (dd, *J* = 2.51, 6.15 Hz, 2H, 4'-CH_2), 1.48 – 1.44 (m, 2H, 5'-CH_2), 1.03 (s, 6H, 8'-CH_3, 9'-CH_3). ^{13}C NMR (101 MHz, $CDCl_3$) δ 169.22(C-1''), 148.57(C-2), 136.64(C-2''), 134.60(C-3'''), 132.63(C-4), 132.18(C-1'), 131.35(C-1'''), 130.33(C-2'''), 123.37(C-6'''), 120.64(C-2'), 105.01(C-3''), 39.50(C-5'), 34.17(C-3'), 33.16(C-6'), 28.89(C-8', C-9'), 21.78(C-7'), 19.04(C-4'), 12.41(C-1). HRMS calculated for $C_{22}H_{26}N_4O_2S$ $[M+H]^+$ 411.1855, found 411.1841.

4-(3-methoxyphenyl)-2-((E)-2-((3E)-4-(2,6,6-trimethylcyclohex-1-en-1-yl)but-3-en-2-ylidene)hydrazin-1-yl)-1,3-thiazole(**1m**)

White solid, Yield 60.18%, FT-IR *v* (cm^{-1}): 3118 (v N-H), 2958, 2934 (v C=C-H), 2863, 2827, 2693 (v C-H), 1613 (v C=N), 1510, 1461.08, 1363 (v Benzene ring), 1289 (v Ph-O-C), 1091 (v C-S-C). ^{1}H NMR (400 MHz, $CDCl_3$) δ 12.40 (s, 1H, NH), 7.42 – 7.27 (m, 3H, 2'''-CH, 5'''-CH, 6'''-CH), 6.96 (d, *J* = 7.64 Hz, 1H, 3''-CH), 6.83 (s, 1H, 4'''-CH), 6.76 (d, *J* = 16.53 Hz, 1H, 4-CH), 6.23 (d, *J* = 16.50 Hz, 1H, 3-CH), 3.89 (s, 3H, OCH_3), 2.31 (s, 3H, 13-CH_3), 2.06 (s, 2H, 3'-CH_2), 1.76 (s, 3H, 7'-CH_3), 1.63 (d, *J* = 6.56 Hz, 2H, 4'-CH_2), 1.51 – 1.47 (m, 2H, 5'-CH_2), 1.07 (s, 6H, 8'-CH_3, 9'-CH_3). ^{13}C NMR (101 MHz, $CDCl_3$) δ 169.02(C-1''), 160.28(C-3'''), 157.36(C-2), 140.38(C-2''), 136.63(C-4), 136.56(C-1'), 132.94(C-1'''), 130.74(C-2'), 130.64(C-5'''), 128.38(C-2'''), 117.81(C-4'''), 116.78(C-6'''), 110.47(C-3''), 101.58(C-3), 55.81(C-7'''), 39.53(C-5'), 34.18(C-3'), 33.21(C-6'), 28.90(C-8', C-9'), 21.76(C-7'), 19.01(C-4'), 13.72(C-1). HRMS calculated for $C_{23}H_{29}N_3OS$ $[M-H]^-$ 394.1953, found 394.1931.

4-(4-fluorophenyl)-2-((E)-2-((3E)-4-(2,6,6-trimethylcyclohex-1-en-1-yl)but-3-en-2-ylidene)hydrazin-1-yl)-1,3-thiazole(**1n**)

Yellow solid, Yield 54.55%, FT-IR *v* (cm^{-1}): 3235(v N-H), 3159, 3024 (v C=C-H), 2950, 2905, 2865, 2694(v C-H), 1622(v C=N), 1580, 1557, 1514 (v Benzene ring), 1096(v C-S-C). ^{1}H NMR (400 MHz, $CDCl_3$) δ 7.72 (dd, *J* = 5.04, 8.58 Hz, 2H, 2'''-CH, 6'''-CH), 7.16 (t, *J* = 8.44 Hz, 2H, 3'''-CH, 5'''-CH), 6.69 (s, 1H, 3''-CH), 6.22 (d, *J* = 16.48 Hz, 1H, 4-CH), 5.06 – 4.82 (m, 1H, 3-CH), 2.30 (s, 3H, 13-CH_3), 2.04 (s, 2H, 3'-CH_2), 1.74 (s, 3H, 7'-CH_3), 1.65 – 1.58 (m, 2H, 4'-CH2), 1.48 (d, *J* = 6.05 Hz, 2H, 5'-CH_2), 1.05 (s, 6H, 8'-CH_3, 9'-CH_3). ^{13}C NMR (101 MHz, $CDCl_3$) δ 169.23(C-1''), 163.67(C-F, $^{4'''}J_{C-F}$=250.5Hz), 157.24(C-2''), 140.20(C-2), 136.60(C-4), 132.94(C-1'), 130.77(C-2'),

127.77(C-F, $^{2'''}J_{C\text{-}F}$=8.8Hz), 124.03(C-F, $^{1'''}J_{C\text{-}F}$=1.8Hz), 116.78(C-F, $^{3'''}J_{C\text{-}F}$=22.2Hz), 100.94(C-$_{3}$), 39.55(C-$_{5'}$), 34.20(C-$_{3'}$), 33.23(C-$_{6'}$), 28.90(C-$_{8'}$, C-$_{9'}$), 21.77(C-$_{7'}$), 19.02(C-$_{4'}$), 13.64(C-$_{1}$). HRMS calculated for $C_{22}H_{26}FN_3S$ $[M+H]^+$ 384.1910, found 384.1909.

4-(4-chlorophenyl)-2-((E)-2-((3E)-4-(2,6,6-trimethylcyclohex-1-en-1-yl)but-3-en-2-yl idene)hydrazin-1-yl)-1,3-thiazole(**1o**)

White solid, Yield 63.92%, FT-IR *ν* (cm^{-1}): 3500 (v $_{N\text{-}H}$), 3220, 3099(v $_{C=C\text{-}H}$), 2927, 2902, 2864, 2827(v $_{C\text{-}H}$), 1613(v $_{C=N}$), 1584, 1488.83, 1362(v $_{Benzene\ ring}$), 1092(v $_{C\text{-}S\text{-}C}$). ^{1}H NMR (400 MHz, $CDCl_3$) δ 12.44 (s, 1H, NH), 7.66 (d, *J* = 8.59 Hz, 2H, $_{2''''}$-CH, $_{6''''}$-CH), 7.45 (d, *J* = 8.38 Hz, 2H, $_{3''''}$-CH, $_{5''''}$-CH), 6.75 (s, 2H, $_{3''}$-CH, $_{4}$-CH), 6.22 (d, *J* = 16.53 Hz, 1H, $_{3}$-CH), 2.31 (s, 3H, $_{13}$-CH_3), 2.05 (s, 2H, $_{3'}$-CH_2), 1.74 (s, 3H, $_{7'}$-CH_3), 1.62 (d, *J* = 2.68 Hz, 2H, $_{4'}$-CH_2), 1.49 (d, *J* = 2.56 Hz, 2H, $_{5'}$-CH_2), 1.05 (s, 6H, $_{8'}$-CH_3, $_{9'}$-CH_3). ^{13}C NMR (101 MHz, $CDCl_3$) δ 169.22(C-$_{1''}$), 157.79(C-$_{2}$), 141.98(C-$_{2''}$), 139.53(C-$_{4}$), 136.93(C-$_{1'}$), 136.55(C-$_{4''''}$), 133.06(C-$_{1''''}$), 130.63(C-$_{2'}$), 129.87(C-$_{3''''}$, C-$_{5''''}$), 126.87(C-$_{2''''}$, C-$_{6''''}$), 125.71(C-$_{3''}$), 101.53(C-$_{3}$), 39.52(C-$_{5'}$), 34.19(C-$_{3'}$), 33.22(C-$_{6'}$), 28.88(C-$_{8'}$, C-$_{9'}$), 21.76(C-$_{7'}$), 19.00(C-$_{4'}$), 13.78(C-$_{1}$). HRMS calculated for $C_{22}H_{26}ClN_3S$ $[M-H]^-$ 398.1458, found 398.1477.

4-(4-bromophenyl)-2-((E)-2-((3E)-4-(2,6,6-trimethylcyclohex-1-en-1-yl)but-3-en-2-yl idene)hydrazin-1-yl)-1,3-thiazole(**1p**)

Yellow solid, Yield 70.30%, FT-IR *ν* (cm^{-1}): 3231(v $_{N\text{-}H}$), 3097, 3085(v $_{C=C\text{-}H}$), 2956, 2923, 2892, 2858, 2818 (v $_{C\text{-}H}$), 1614 (v $_{C=N}$), 1587, 1493, 1361(v $_{Benzene\ ring}$), 1095(v $_{C\text{-}S\text{-}C}$). ^{1}H NMR (400 MHz, $CDCl_3$) δ 12.41 (s, 1H, NH), 7.59 (s, 4H, $_{2''''}$-CH, $_{6''''}$-CH, $_{3''''}$-CH, $_{5''''}$-CH), 6.76 (d, *J* = 21.81 Hz, 2H, $_{3''}$-CH, $_{4}$-CH), 6.22 (d, *J* = 16.48 Hz, 1H, $_{3}$-CH), 2.30 (s, 3H, $_{13}$-CH_3), 2.04 (s, 2H, $_{3'}$-CH_2), 1.74 (s, 3H, $_{7'}$-CH_3), 1.65 – 1.58 (m, 2H, $_{4'}$-CH_2), 1.48 (d, *J* = 5.98 Hz, 2H, $_{5'}$-CH_2), 1.05 (s, 6H, $_{8'}$-CH_3, $_{9'}$-CH_3). ^{13}C NMR (101 MHz, $CDCl_3$) δ 169.17(C-$_{1''}$), 157.74(C-$_{2}$), 139.53(C-$_{2''}$), 136.91(C-$_{4}$), 136.54(C-$_{1'}$), 133.07(C-$_{1''''}$), 132.80(C-$_{3''''}$, C-$_{5''''}$), 130.64(C-$_{2'}$), 127.03(C-$_{2''''}$, C-$_{6''''}$), 126.14(C-$_{4''''}$), 124.76(C-$_{3''}$), 101.75(C-$_{3}$), 39.50(C-$_{5'}$), 34.18(C-$_{3'}$), 33.22(C-$_{6'}$), 28.89(C-$_{8'}$, C-$_{9'}$), 21.78(C-$_{7'}$), 18.99(C-$_{4'}$), 13.76(C-$_{1}$). HRMS calculated for $C_{22}H_{26}BrN_3S$ $[M+2H]^+$ 445.1187, found 445.1139.

4-(2-(2-((2E,3E)-4-(2,6,6-trimethylcyclohex-1-en-1-yl)but-3-en-2-ylidene)hydrazinyl) thiazol-4-yl)phenol(**1q**)

Yellow solid, Yield 59.70%, FT-IR *v* (cm^{-1}):3306 (v $_{O-H}$), 3224 (v $_{N-H}$), 3095, 3021 (v $_{C=C-H}$), 2931, 2862 (v $_{C-H}$), 1610 (v $_{C=N}$), 1515, 1431, 1360 (v $_{Benzene\ ring}$), 1080(v $_{C-S-C}$). ^{1}H NMR (400 MHz, CDCl$_3$) δ 12.26 (s, 1H, NH), 7.56 – 7.49 (m, 2H, 2'''-CH, 6'''-CH), 7.02 (d, J = 8.70 Hz, 2H, 3'''-CH, 5'''-CH), 6.76 (d, J = 16.33 Hz, 1H, 3''-CH), 6.53 (s, 1H, 4-CH), 6.23 (d, J = 16.48 Hz, 1H, 3-CH), 2.32 (s, 3H, 13-CH$_3$), 2.06 (t, J = 6.26 Hz, 2H, 3'-CH$_2$), 1.75 (s, 3H, 7'-CH$_3$), 1.63 (dd, J = 7.78, 4.43 Hz, 2H, 4'-CH$_2$), 1.51 – 1.47 (m, 2H, 5'-CH$_2$), 1.07 (s, 6H, 8'-CH$_3$, 9'-CH$_3$). ^{13}C NMR (101 MHz, CDCl$_3$) δ 169.06(C-1''), 158.44(C-4'''), 157.41(C-2''), 140.84(C-2), 136.67(C-1'), 136.62(C-2'), 132.89(C-4), 130.80(C-1'''), 127.20(C-2''', C-6'''), 119.38(C-2'), 116.71(C-3''', C-5'''), 98.59(C-3), 39.57(C-5'), 34.22(C-3'), 33.23(C-6'), 28.91(C-8', C-9'), 21.76(C-7'), 19.04(C-4'), 13.73(C-1). HRMS calculated for $C_{22}H_{27}N_3OS$ $[M+H]^+$ 382.1953, found 382.1941.

4-(4-nitrophenyl)-2-((E)-2-((3E)-4-(2,6,6-trimethylcyclohex-1-en-1-yl)but-3-en-2-ylidene)hydrazin-1-yl)-1,3-thiazole(**1r**)

Yellow solid, Yield 71.02%, FT-IR *v* (cm^{-1}): 3330.68(v $_{N-H}$), 3110 (v $_{C=C-H}$), 2927, 2861 (v $_{C-H}$), 1599(v $_{C=N}$), 1599, 1562, 1453 (v $_{Benzene\ ring}$), 1508, 1340 (Ph-NO$_2$) 1110(v $_{C-S-C}$). ^{1}H NMR (400 MHz, CDCl$_3$) δ 8.23 (d, *J* = 8.43 Hz, 2H, 3'''-CH, 5'''-CH), 7.91 (d, *J* = 8.33 Hz, 2H, 2'''-CH, 6'''-CH), 7.09 (s, 1H, 3''-CH), 6.48 (d, *J* = 16.67 Hz, 1H, 4-CH), 6.22 (d, *J* = 16.17 Hz, 1H, 3-CH), 2.00 (d, *J* = 10.14 Hz, 5H, 13-CH$_3$, 4-CH$_2$), 1.73 (s, 3H, 7'-CH$_3$), 1.61 (s, 2H, 4'-CH$_2$), 1.46 (s, 2H, 5'-CH$_2$), 1.03 (s, 6H, 8'-CH$_3$, 9'-CH$_3$). ^{13}C NMR (101 MHz, CDCl$_3$) δ 169.41(C-1''), 149.00(C-2), 148.72(C-2''), 146.84(C-4'''), 140.42(C-4), 136.74(C-1'''), 132.33(C-1'), 131.21(C-2'), 126.28(C-2''', C-6'''), 124.13(C-3''', C-5'''), 107.66(C-3), 39.47(C-5'), 34.16(C-3'), 33.05(C-6'), 28.87(C-8', C-9'), 21.75(C-7'), 19.11(C-4'), 11.15(C-1). HRMS calculated for $C_{22}H_{26}N_4O_2S$ $[M+H]^+$ 411.1855, found 411.1860.

4-(4-methoxyphenyl)-2-((E)-2-((3E)-4-(2,6,6-trimethylcyclohex-1-en-1-yl)but-3-en-2-ylidene)hydrazin-1-yl)-1,3-thiazole(**1s**)

Yellow solid, Yield 66.72%, FT-IR *v* (cm^{-1}): 3324 (v $_{N-H}$), 3008 (v $_{C=C-H}$), 2923, 2863(v $_{C-H}$), 1614(v $_{C=N}$), 1583, 1504, 1433(v $_{Benzene\ ring}$), 1257 (Ph-O-C), 1094(v $_{C-S-C}$). ^{1}H NMR (400 MHz, CDCl$_3$) δ 12.44 (s, 1H, NH), 7.66 (d, *J* = 6.84 Hz, 2H, 2'''-CH, 6'''-CH), 7.01 – 6.95 (m, 2H, 3'''-CH, 5'''-CH), 6.74 (d, *J* = 16.52 Hz, 1H, 3''-CH), 6.57 (d, *J* = 1.79 Hz, 1H, 4-CH), 6.22 (dd, *J* = 1.87, 16.51 Hz, 1H, 3-CH), 3.83 (s, 3H, OCH$_3$), 2.31 (s, 3H, 13-CH$_3$), 2.04 (d, *J* = 6.53 Hz, 2H, 3'-CH$_2$), 1.74 (s, 3H, 7'-CH$_3$), 1.62 (d, *J*

= 6.62 Hz, 2H, 4'-CH2), 1.48 (d, J = 6.13 Hz, 2H, 5'-CH_2), 1.05 (s, 6H, 8'-CH_3, 9'-CH_3). ^{13}C NMR (101 MHz, $CDCl_3$) δ 169.09(C-1''), 161.14(C-4'''), 157.34(C-2), 140.54(C-2''), 136.57(C-4), 132.85(C-1'), 130.80(C-2'), 127.15(C-2''', C-6'''), 119.88(C-1'''), 114.93(C-3''', C-5'''), 101.97(C-3''), 98.74(C-3), 55.46(C-7'''), 39.51(C-5'), 34.18(C-3'), 33.20(C-6'), 28.89(C-8', C-9'), 21.76(C-7'), 19.01(C-4'), 13.76(C-1). HRMS calculated for $C_{23}H_{29}N_3OS$ [M-H]$^-$ 394.1953, found 394.1968.

4-(4-methylphenyl)-2-((E)-2-((3E)-4-(2,6,6-trimethylcyclohex-1-en-1-yl)but-3-en-2-ylidene)hydrazin-1-yl)-1,3-thiazole(**1t**)

Yellow solid, Yield 66.65%, FT-IR ν (cm^{-1}): 3231(v N-H), 3131, 3027(v C=C-H), 2924, 2860, 2827, 2714 (v C-H), 1619(v C=N), 1583, 1502, 1361 (v Benzene ring), 1094(v C-S-C). ^{1}H NMR (400 MHz, $CDCl_3$) δ 12.43 (s, 1H, NH), 7.58 (d, J = 8.43 Hz, 2H, 2'''-CH, 6'''-CH), 7.24 (d, J = 7.90 Hz, 1H, 3''-CH), 6.76 – 6.68 (m, 2H, 3'''-CH, 5'''-CH), 6.21 (d, J = 16.46 Hz, 1H, 4-CH), 5.10 (s, 1H, 3-CH), 2.35 (s, 3H, 7'''-CH_3), 2.30 (s, 3H, 13-CH_3), 2.03 (s, 2H, 3'-CH_2), 1.73 (s, 3H, 7'-CH_3), 1.65 – 1.57 (m, 2H, 4'-CH_2), 1.47 (d, J = 6.05 Hz, 2H, 5'-CH_2), 1.04 (s, 6H, 8'-CH_3, 9'-CH_3). ^{13}C NMR (101 MHz, $CDCl_3$) δ 169.09(C-1''), 157.33(C-2), 140.75(C-2''), 136.58(C-4), 132.88(C-1'), 130.78(C-3''', C-5'''), 130.20(C-2'), 125.47(C-2''', C-6'''), 124.47(C-3''), 100.21(C-3), 39.49(C-5'), 34.17(C-3'), 33.19(C-6'), 28.88(C-8', C-9'), 21.77(C-7'''), 21.41(C-7'), 19.00(C-4'), 13.78(C-1). HRMS calculated for $C_{23}H_{29}N_3S$ [M+H]$^+$ 380.2160, found 380.2165.

4-(4-(trifluoromethyl)phenyl)-2-((E)-2-((3E)-4-(2,6,6-trimethylcyclohex-1-en-1-yl)but-3-en-2-ylidene)hydrazin-1-yl)-1,3-thiazole(**1u**)

Yellow solid, Yield 55.73%, FT-IR ν (cm^{-1}): 3361 (v N-H), 3122, 3037(v C=C-H), 2961, 2930, 2864, 2827(v C-H), 1614 (v C=N), 1585, 1504, 1328(v Benzene ring), 1070(v C-S-C). ^{1}H NMR (400 MHz, $CDCl_3$) δ 7.86 (d, J = 8.13 Hz, 2H, 3'''-CH, 5'''-CH), 7.69 (d, J = 8.21 Hz, 2H, 2'''-CH, 6'''-CH), 6.90 (s, 1H, 3''-CH), 6.68 (d, J = 16.52 Hz, 1H, 4-CH), 6.23 (d, J = 16.63 Hz, 1H, 3-CH), 2.22 (s, 3H, 13-CH_3), 2.04 (s, 2H, 3'-CH_2), 1.74 (s, 3H, 7'-CH_3), 1.61 (d, J = 6.18 Hz, 2H, 4'-CH_2), 1.50 – 1.46 (m, 2H, 5'-CH_2), 1.05 (s, 6H, 8'-CH_3, 9'-CH_3). ^{13}C NMR (101 MHz, $CDCl_3$) δ 169.23(C-1''), 154.82(C-2), 142.75(C-2''), 136.66(C-4), 135.39(C-1'), 133.02(C-1'''), 132.50(C-4'''), 131.37(C-2'), 131.05(C-2'''), 126.30(C-6'''), 126.02(C-3''', C-5'''), 125.10(C-7'''), 122.40(C-3''), 104.11(C-3), 39.55(C-5'), 34.20(C-3'), 33.21(C-6'), 28.90(C-8', C-9'), 21.77(C-7'),

19.05(C-4'), 12.85(C-1). HRMS calculated for $C_{23}H_{26}F_3N_3S$ $[M+H]^+$ 434.1878, found 434.1876.

4-(4-phenylphenyl)-2-((E)-2-((3E)-4-(2,6,6-trimethylcyclohex-1-en-1-yl)but-3-en-2-ylidene)hydrazin-1-yl)-1,3-thiazole(**1v**)

Yellow solid, Yield 74.00%, FT-IR *v* (cm^{-1}): 3120 (v_{N-H}), 3031($v_{C=C-H}$), 2922, 2859, 2751 (v_{C-H}), 1607 ($v_{C=N}$), 1584, 1486($v_{Benzene\ ring}$), 1106 (v_{C-S-C}). ^1H NMR (400 MHz, $CDCl_3$) δ 12.46 (s, 1H, NH), 7.76 (d, *J* = 8.10 Hz, 2H, 2'''-CH, 6'''-CH), 7.67 (d, *J* = 8.06 Hz, 2H, 3'''-CH, 5'''-CH), 7.56 (d, *J* = 7.11 Hz, 2H, 8'''-CH, 12'''-CH), 7.45 – 7.34 (m, 3H, 9'''-CH, 10'''-CH, 11'''-CH), 6.79 (s, 2H, 3''-CH, 4-CH), 6.21 (d, *J* = 16.50 Hz, 1H, 3-CH), 2.29 (s, 3H, 13-CH_3), 2.04 (s, 2H, 3'-CH_2), 1.73 (s, 3H, 7'-CH_3), 1.61 (d, *J* = 6.59 Hz, 2H, 4'-CH2), 1.50 – 1.45 (m, 2H, 5'-CH_2), 1.05 (s, 6H, 8'-CH_3, 9'-CH_3). ^{13}C NMR (101 MHz, $CDCl_3$) δ 169.17(C-1''), 157.52(C-2), 142.97(C-2''), 140.22(C-4), 139.48(C-4'''), 136.69(C-7'''), 136.58(C-1'), 133.00(C-1'''), 130.73(C-2'), 129.00(C-9''', C-11'''), 128.08(C-10'''), 128.06(C-2''', C-6'''), 127.00(C-8''', C-12'''), 126.02(C-3''', C-5'''), 125.99(C-3''), 101.13(C-3), 39.56(C-5'), 34.21(C-3'), 33.25(C-6'), 28.92(C-8', C-9'), 21.79(C-7'), 19.03(C-4'), 13.79(C-1). HRMS calculated for $C_{28}H_{31}N_3S$ $[M+H]^+$ 442.2317, found 442.2341.

4-(2,4-difluorophenyl)-2-((E)-2-((3E)-4-(2,6,6-trimethylcyclohex-1-en-1-yl)but-3-en-2-ylidene)hydrazin-1-yl)-1,3-thiazole(**1w**)

Yellow solid, Yield 60.66%, FT-IR *v* (cm^{-1}): 3245(v_{N-H}), 3155, 3024 ($v_{C=C-H}$), 2929, 2866, 2829, 2688(v_{C-H}), 1624($v_{C=N}$), 1579, 1563, 1503($v_{Benzene\ ring}$), 1094(v_{C-S-C}). ^1H NMR (400 MHz, $CDCl_3$) δ 11.87 (s, 1H, NH), 7.88 (s, 1H, 6'''-CH), 7.00 (d, *J* = 9.69 Hz, 2H, 5'''-CH, 3''-CH), 6.92 (d, *J* = 2.76 Hz, 1H, 3'''-CH), 6.71 (d, *J* = 16.70 Hz, 1H, 4-CH), 6.22 – 6.15 (m, 1H, 3-CH), 2.27 (d, *J* = 4.20 Hz, 3H, 13-CH_3), 2.01 (d, *J* = 5.64 Hz, 2H, 3'-CH_2), 1.70 (s, 3H, 7'-CH_3), 1.58 (d, *J* = 5.50 Hz, 2H, 4'-CH2), 1.44 (d, *J* = 6.52 Hz, 2H, 5'-CH_2), 1.01 (s, 6H, 8'-CH_3, 9'-CH_3). ^{13}C NMR (101 MHz, $CDCl_3$) δ 168.62(C-1''), 164.63(C-F, $^{4'''}J_{C-F}$=12.6Hz), 158.11(C-F, $^{2'''}J_{C-F}$=143.6Hz), 136.60(C-F, $^{6'''}J_{C-F}$=17.9Hz), 133.79(C-2''), 132.97(C-2), 130.66(C-4), 129.09(C-F, $^{1'''}J_{C-F}$=9.2Hz), 112.89(C-F, $^{3'''}J_{C-F}$=21.6Hz), 105.41(C-F, $^{5'''}J_{C-F}$=26.0Hz), 39.49(C-5'), 34.14(C-3'), 33.18(C-6'), 28.85(C-8', C-9'), 21.73(C-7'), 18.96(C-4'), 13.64(C-1). HRMS calculated for $C_{22}H_{25}F_2N_3S$ $[M+H]^+$ 402.1815, found 402.1802.

4-(3,4-dichlorophenyl)-2-((E)-2-((3E)-4-(2,6,6-trimethylcyclohex-1-en-1-yl)but-3-en-2-ylidene)hydrazin-1-yl)-1,3-thiazole(**1x**)

Yellow solid, Yield 72.26%, FT-IR *v* (cm^{-1}): 3231(v_{N-H}), 3100, 3051 ($v_{C=C-H}$), 2956, 2925, 2862, 2822 (v_{C-H}), 1617($v_{C=N}$), 1584, 1510, 1476 ($v_{Benzene\ ring}$), 1094(v_{C-S-C}). ^1H NMR (400 MHz, $CDCl_3$) δ 12.42 (s, 1H, NH), 7.78 (d, *J* = 2.14 Hz, 1H, $_{2'''}$-CH), 7.65 – 7.56 (m, 2H, $_{6'''}$-CH, $_{5'''}$-CH), 6.80 (s, 2H, $_{3''}$-CH, $_4$-CH), 6.22 (d, *J* = 16.49 Hz, 1H, $_3$-CH), 2.31 (s, 3H, $_{13}$-CH_3), 2.04 (d, *J* = 6.45 Hz, 2H, $_{3'}$-CH_2), 1.75 (s, 3H, $_{7'}$-CH_3), 1.65 – 1.59 (m, 2H, $_{4'}$-CH2), 1.50 – 1.46 (m, 2H, $_{5'}$-CH_2), 1.06 (s, 6H, $_{8'}$-CH_3, $_{9'}$-CH_3). ^{13}C NMR (101 MHz, $CDCl_3$) δ 169.28(C-$_{1''}$), 158.00(C-$_2$), 138.46(C-$_{2''}$), 137.09(C-$_4$), 136.55(C-$_{1'}$), 134.79(C-$_{4'''}$), 134.00(C-$_{3'''}$), 133.15(C-$_{1'''}$), 131.73(C-$_{5'''}$), 130.55(C-$_{2'}$), 127.40(C-$_{2'''}$), 127.12(C-$_{6'''}$), 124.78(C-$_{3''}$), 102.58(C-$_3$), 39.53(C-$_{5'}$), 34.19(C-$_{3'}$), 33.23(C-$_{6'}$), 28.88(C-$_{8'}$, C-$_{9'}$), 21.75(C-$_{7'}$), 18.99(C-$_{4'}$), 13.78(C-$_1$). HRMS calculated for $C_{22}H_{25}Cl_2N_3S$ $[M+H]^+$ 434.1224, found 434.1226.

4-(2,5-dimethoxyphenyl)-2-((E)-2-((3E)-4-(2,6,6-trimethylcyclohex-1-en-1-yl)but-3-en-2-ylidene)hydrazin-1-yl)-1,3-thiazole(**1y**)

Yellow solid, Yield 73.88%, FT-IR *v* (cm^{-1}): 3388(v_{N-H}), 3180, 3086($v_{C=C-H}$), 2928, 2862, 2833(v_{C-H}), 1602 ($v_{C=N}$), 1494, 1471 ($v_{Benzene\ ring}$), 1237, 1212 (v_{Ph-O-C}) 1080 (v_{C-S-C}). ^1H NMR (400 MHz, $CDCl_3$) δ 13.21 (s, 1H, NH), 7.15 (s, 1H, $_{6'''}$-CH), 6.95 (s, 1H, $_{3'''}$-CH), 6.91 (d, *J* = 1.65 Hz, 2H, $_{4'''}$-CH, $_{3''}$-CH), 6.69 (d, *J* = 16.49 Hz, 1H, $_4$-CH), 6.19 (d, *J* = 16.51 Hz, 1H, $_3$-CH), 4.00 (s, 3H, OCH_3), 3.81 (s, 3H, OCH_3), 2.33 (s, 3H, $_{13}$-CH_3), 2.01 (t, *J* = 6.41 Hz, 2H, $_{3'}$-CH_2), 1.71 (s, 3H, $_{7'}$-CH_3), 1.58 (d, *J* = 6.16 Hz, 2H, $_{4'}$-CH2), 1.47 – 1.43 (m, 2H, $_{5'}$-CH_2), 1.02 (s, 6H, $_{8'}$-CH_3, $_{9'}$-CH_3). ^{13}C NMR (101 MHz, $CDCl_3$) δ 168.44(C-$_{1''}$), 157.37(C-$_{5'''}$), 153.73(C-$_2$), 150.47(C-$_{2'''}$), 137.61(C-$_{2''}$), 136.61(C-$_4$), 136.30(C-$_{1'}$), 132.64(C-$_{2'}$), 130.97(C-$_{1'''}$), 117.30(C-$_{4'''}$), 116.06(C-$_{3'''}$), 112.89(C-$_{6'''}$), 112.68(C-$_{3''}$), 103.72(C-$_3$), 56.42(C-$_{8'''}$), 56.23(C-$_{7'''}$), 39.53(C-$_{5'}$), 34.18(C-$_{3'}$), 33.18(C-$_{6'}$), 28.88(C-$_{8'}$, C-$_{9'}$), 21.74(C-$_{7'}$), 19.03(C-$_{4'}$), 14.36(C-$_1$). HRMS calculated for $C_{24}H_{31}N_3O_2S$ $[M-H]^-$ 424.2059, found 424.2031.

2.Spectrums of compounds 3 and 1a~1y

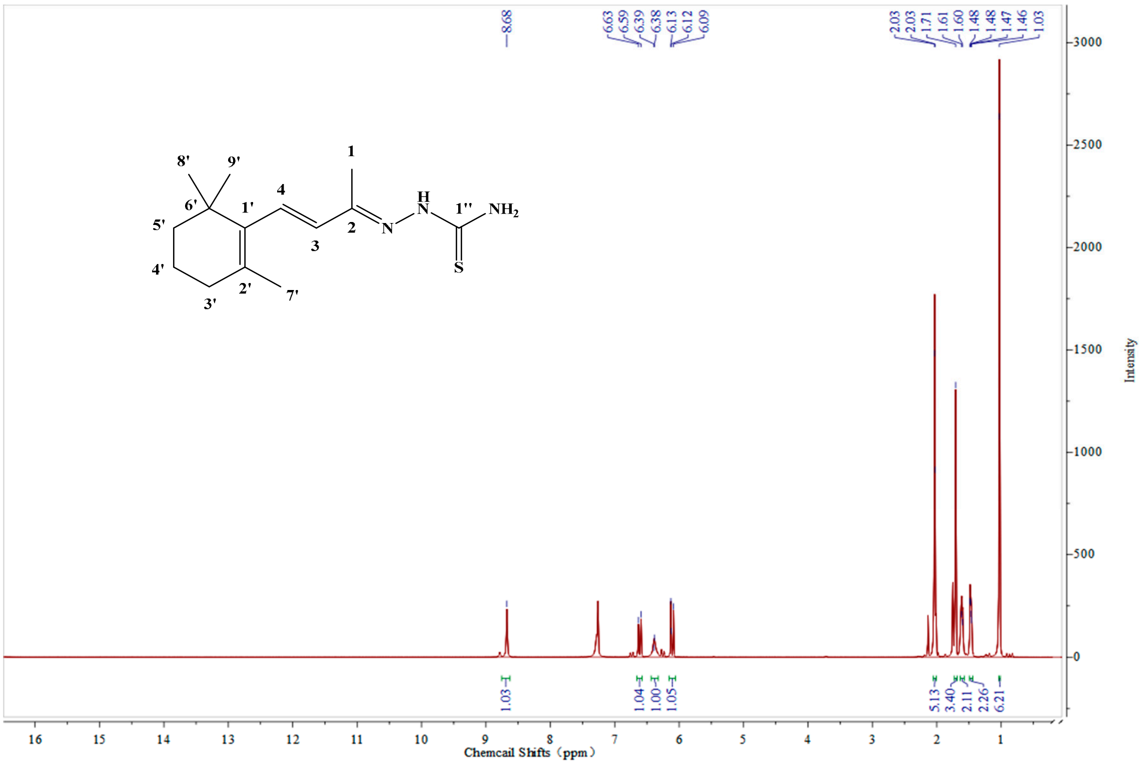

Figure S1. The ^{1}H NMR spectra Compound 3

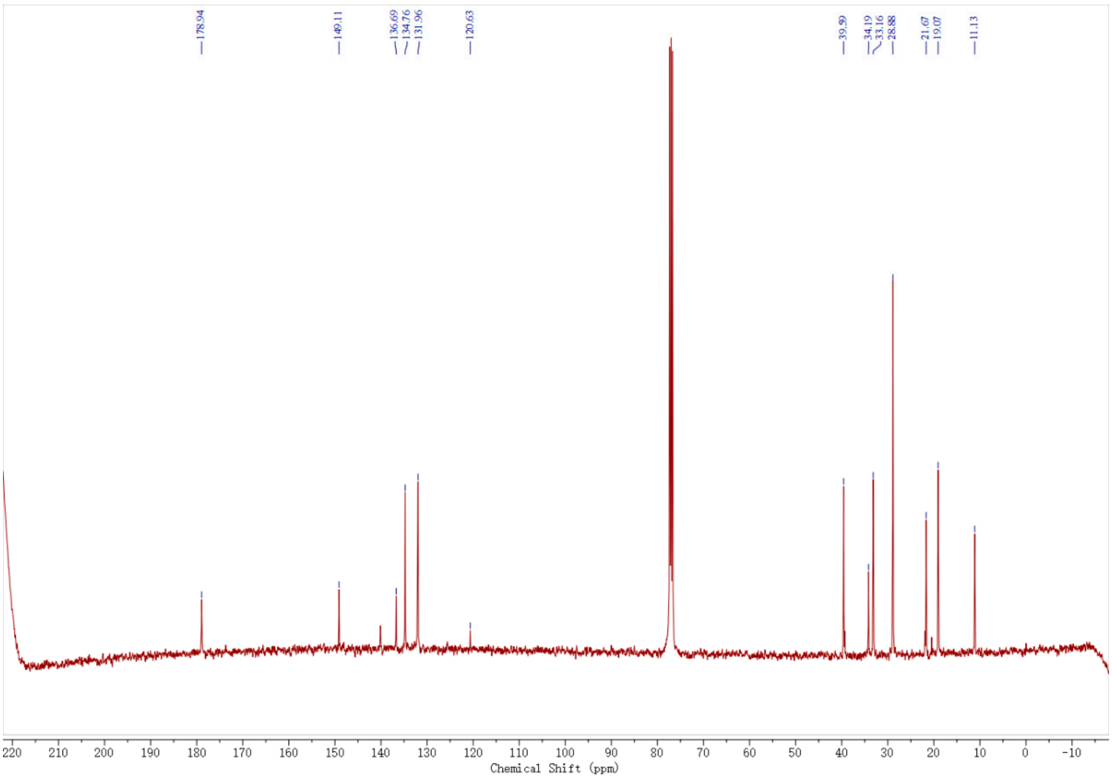

Figure S2. The ^{13}C NMR spectra of Compound 3

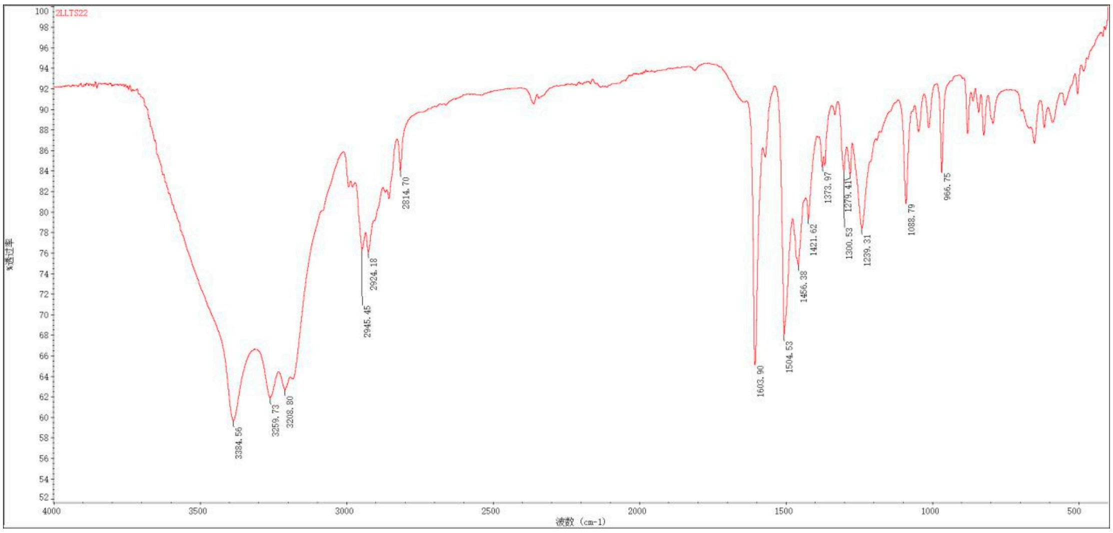

Figure S3. The FT-IR spectra of Compound 3

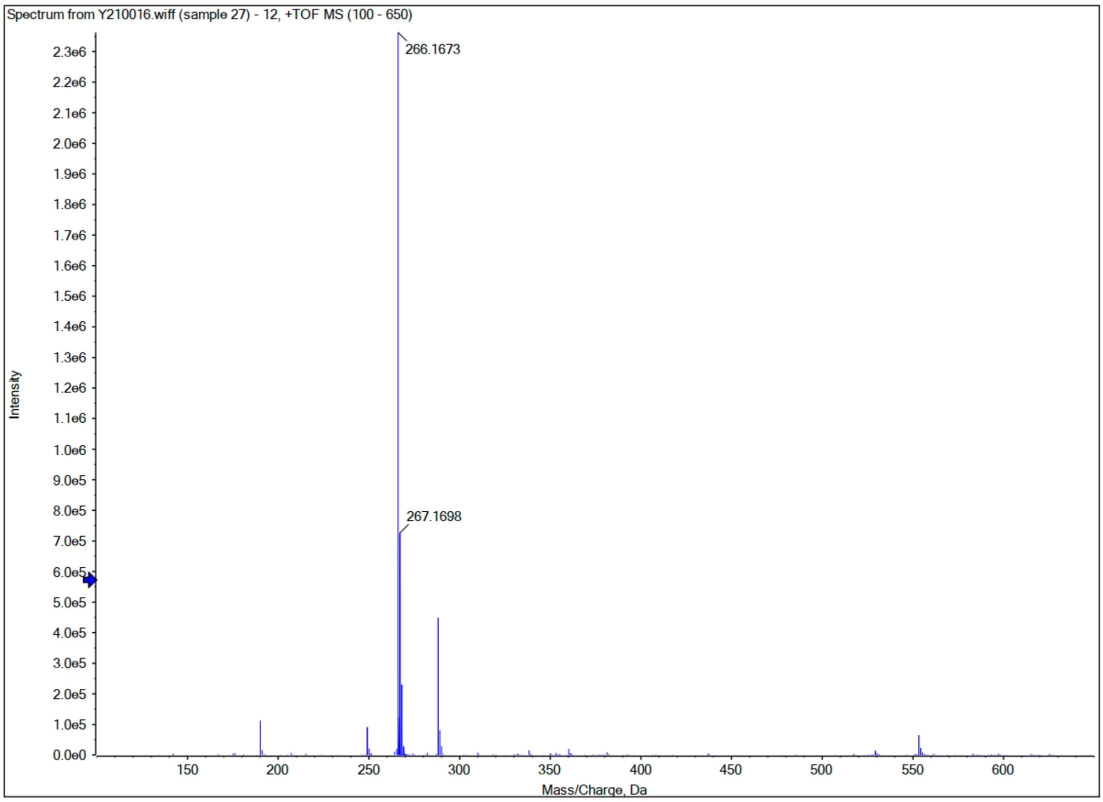

Figure S4. The HRMS spectra of Compound 3

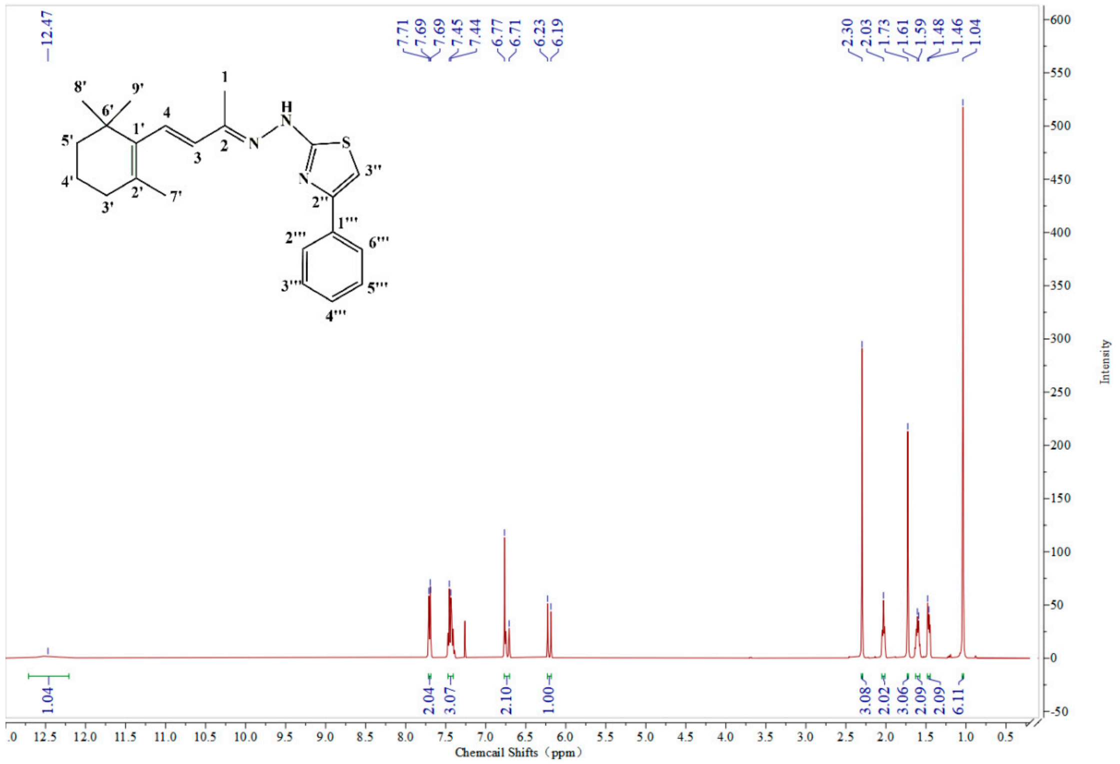

Figure S5. The ^{1}H NMR spectra Compound 1a

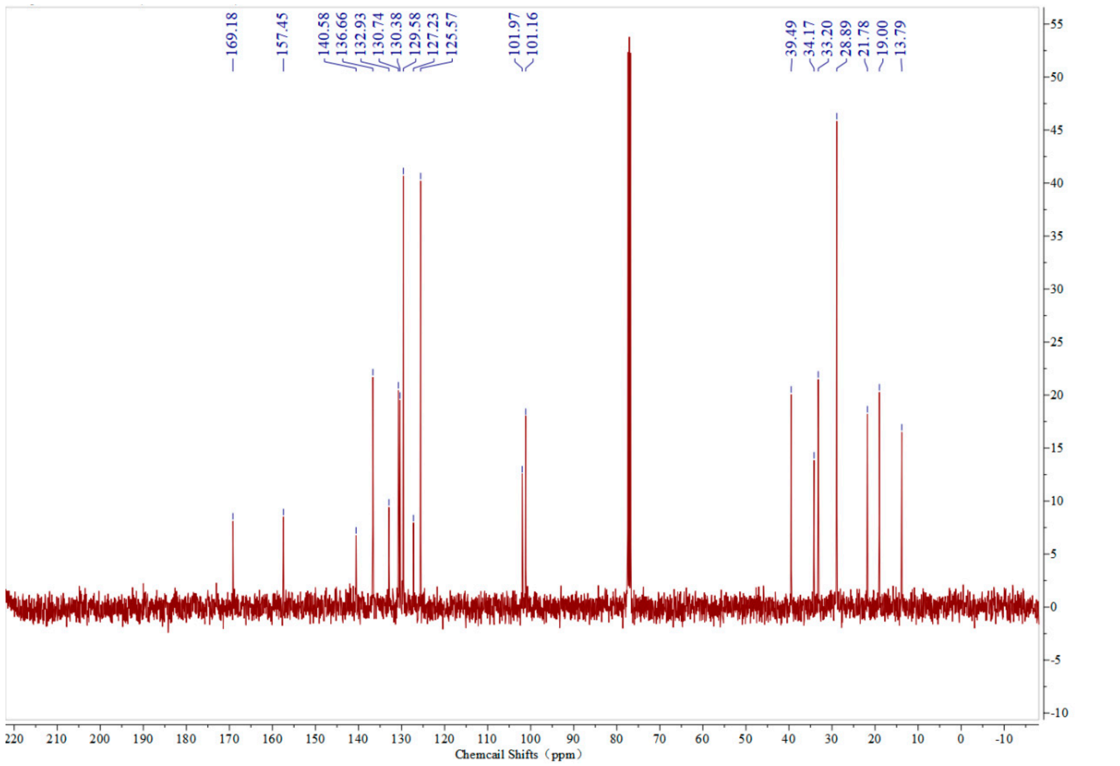

Figure S6. The ^{13}C NMR spectra of Compound 1a

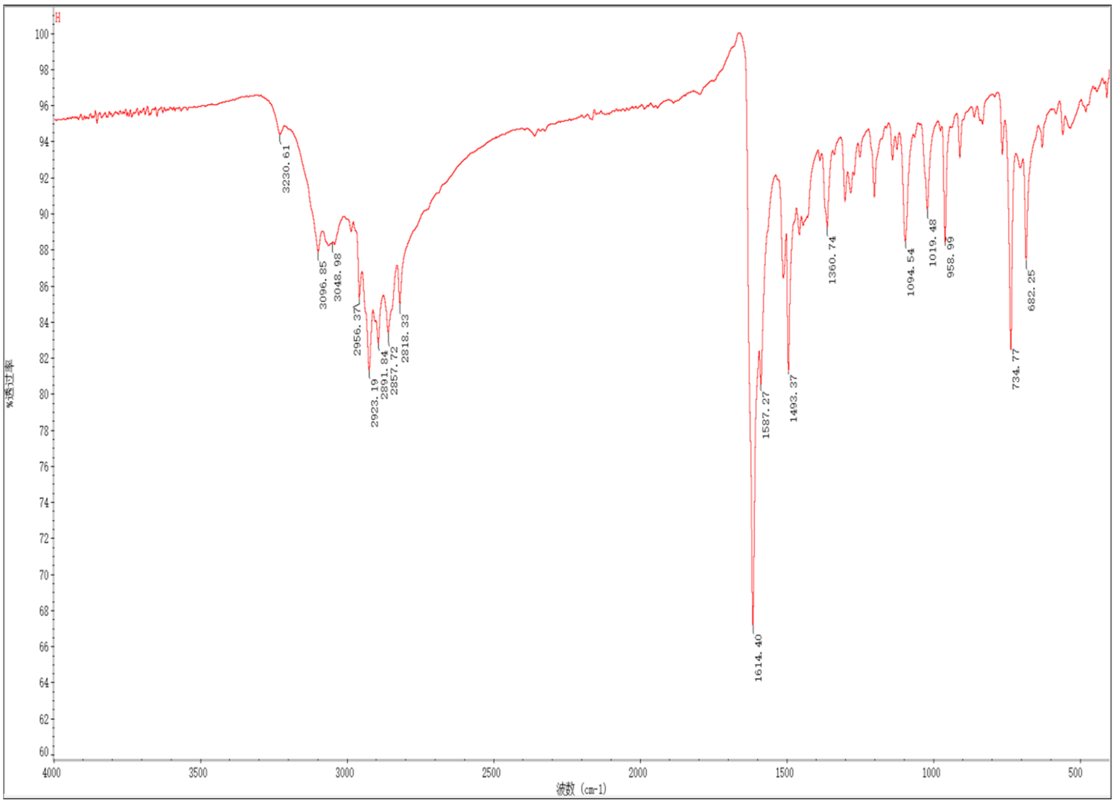

Figure S7. The FT-IR spectra of Compound 1a

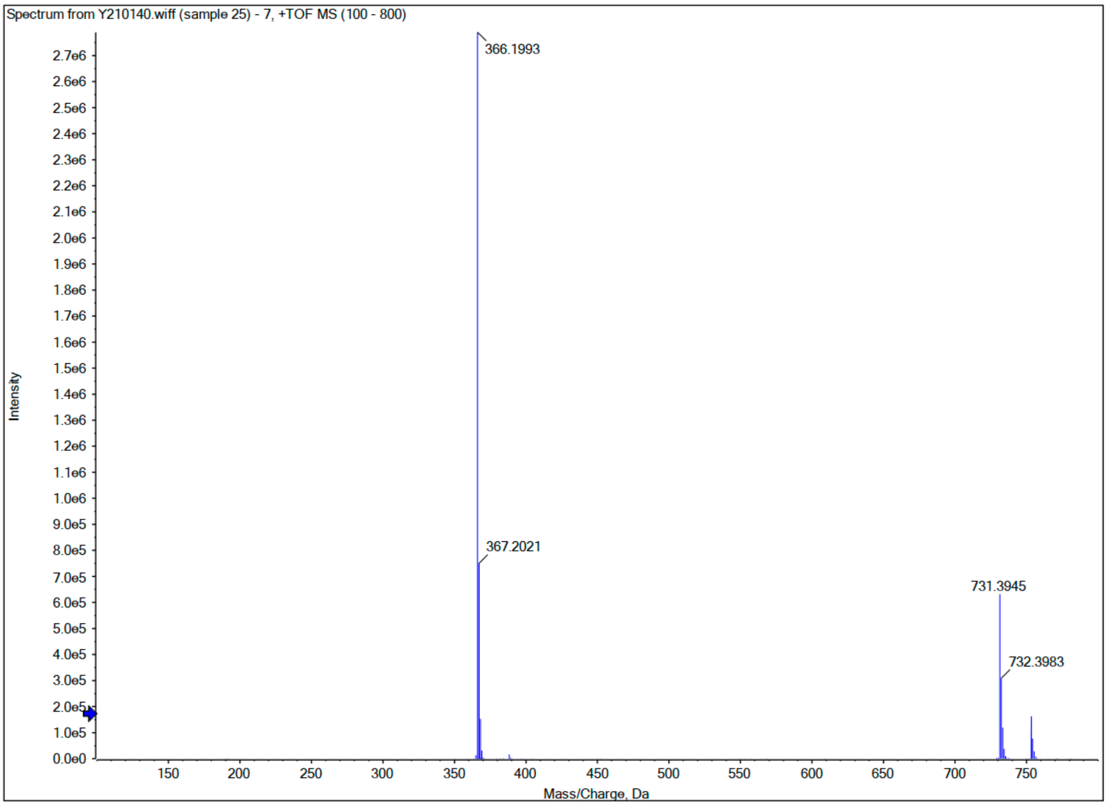

Figure S8. The HRMS spectra of Compound 1a

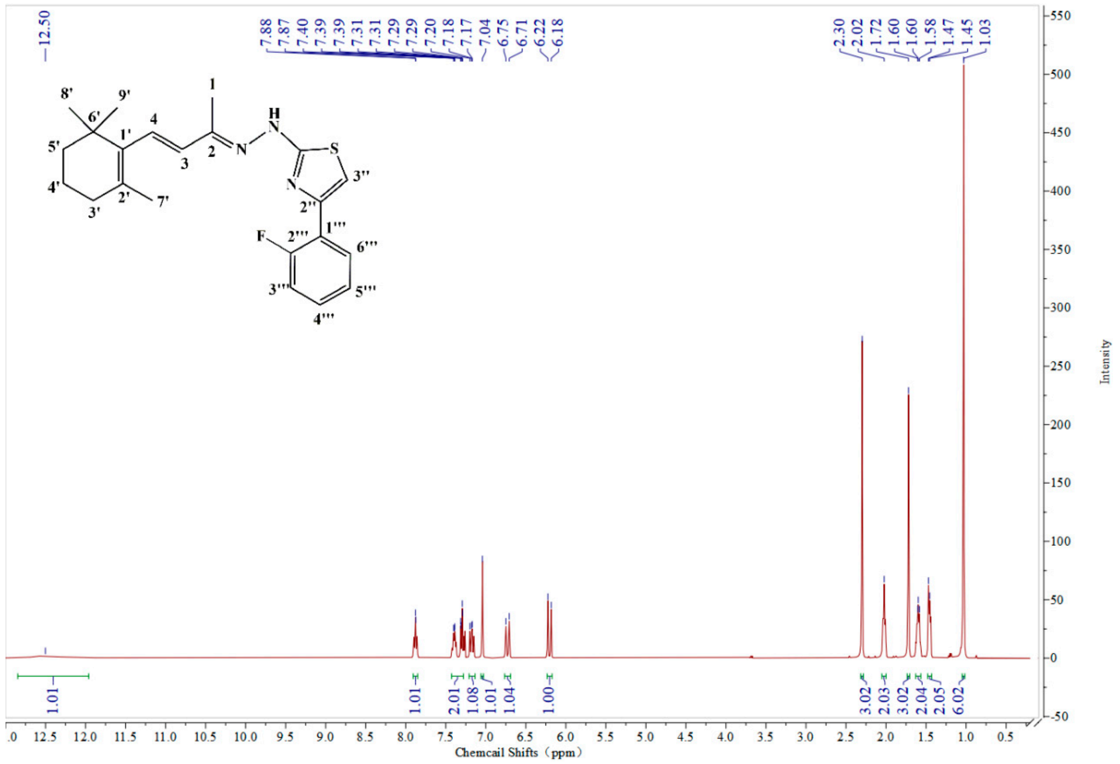

Figure S9. The ^{1}H NMR spectra Compound 1b

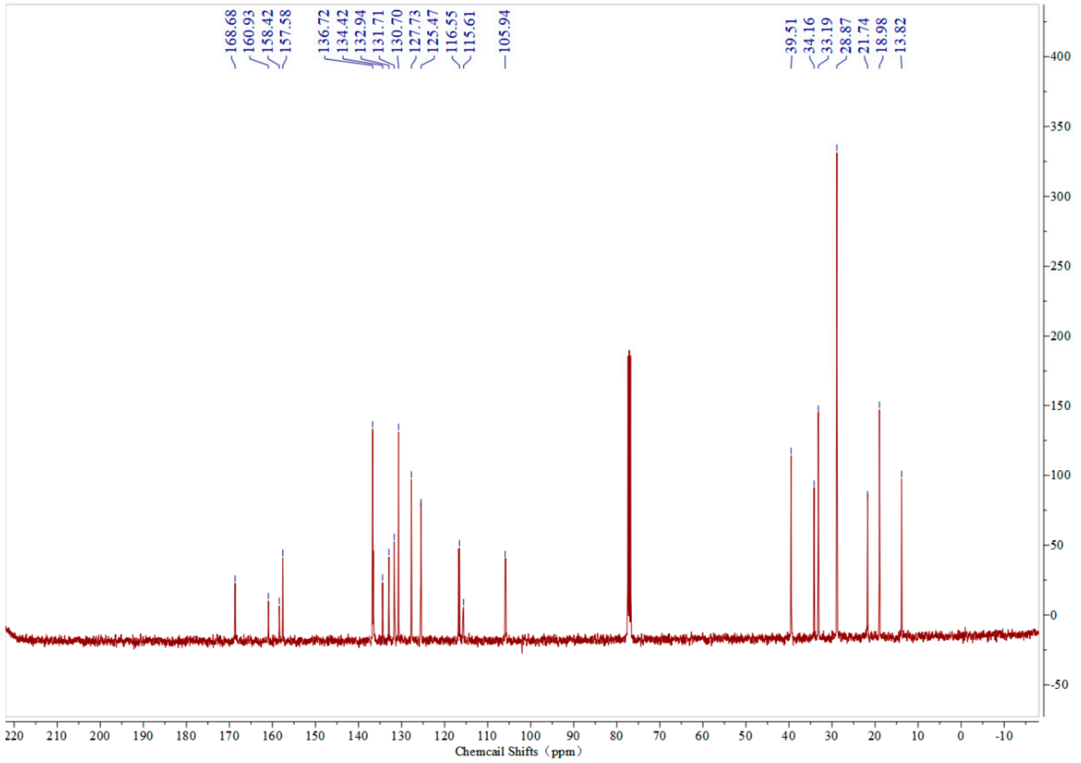

Figure S10. The ^{13}C NMR spectra of Compound 1b

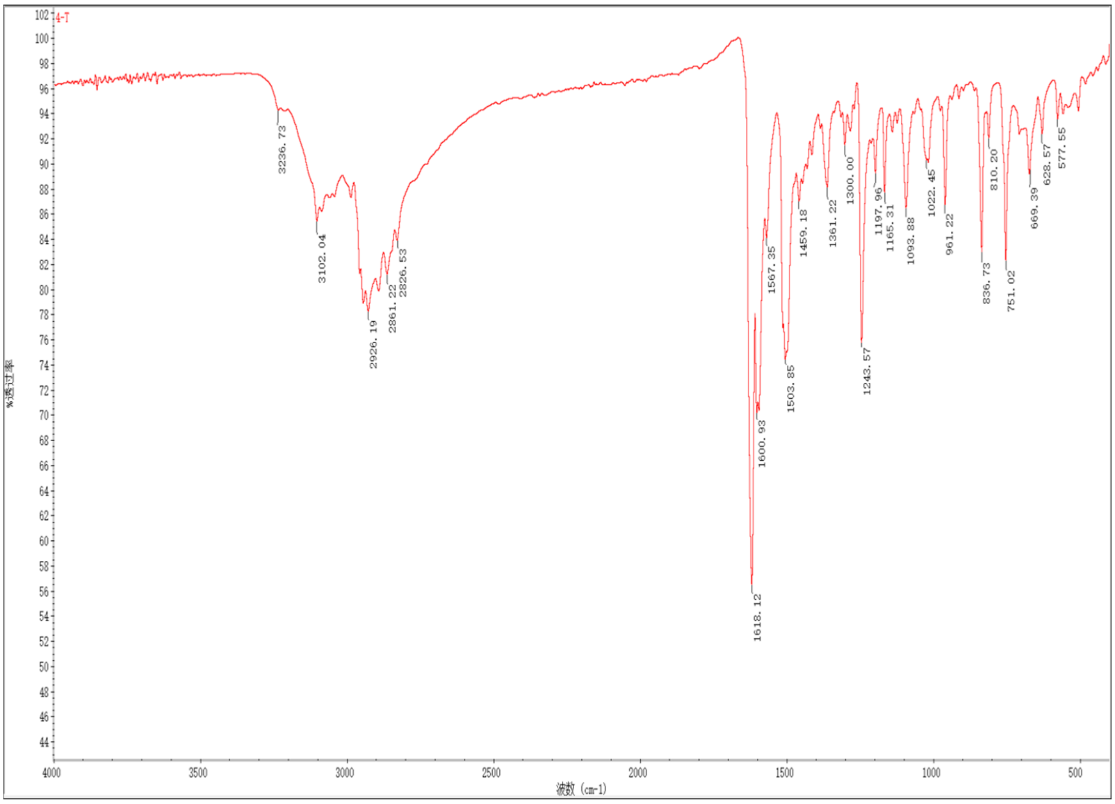

Figure S11. The FT-IR spectra of Compound 1b

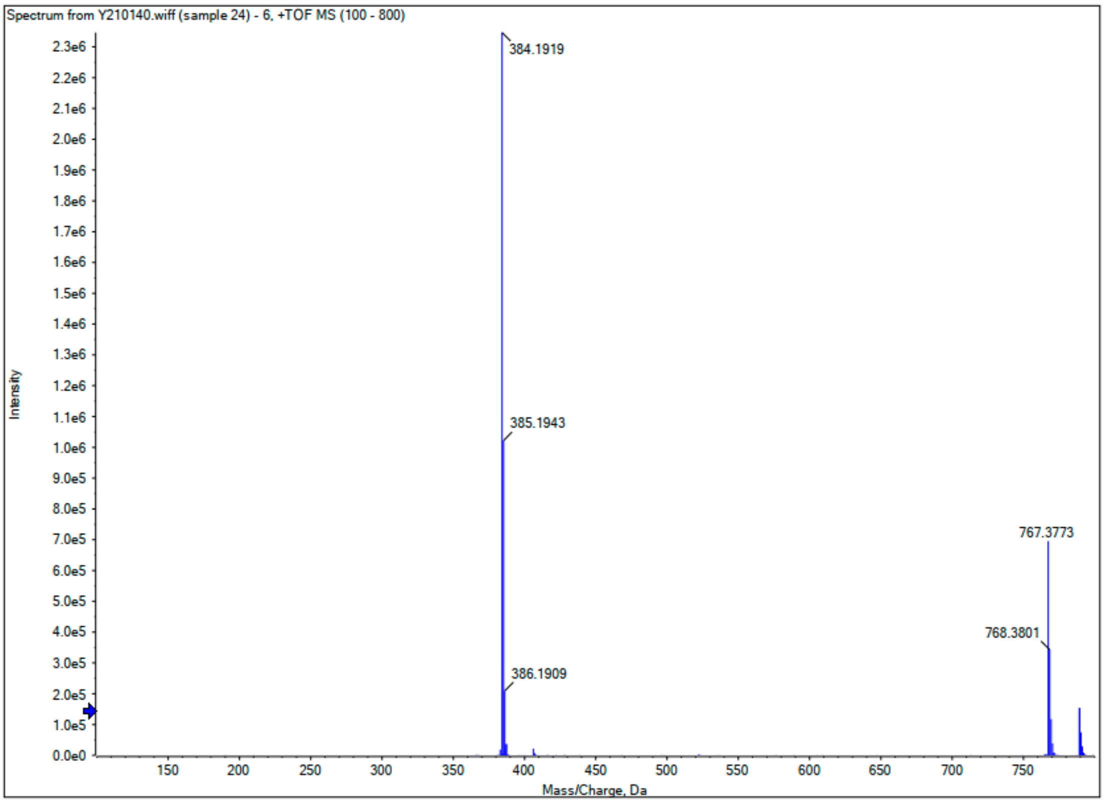

Figure S12. The HRMS spectra of Compound 1b

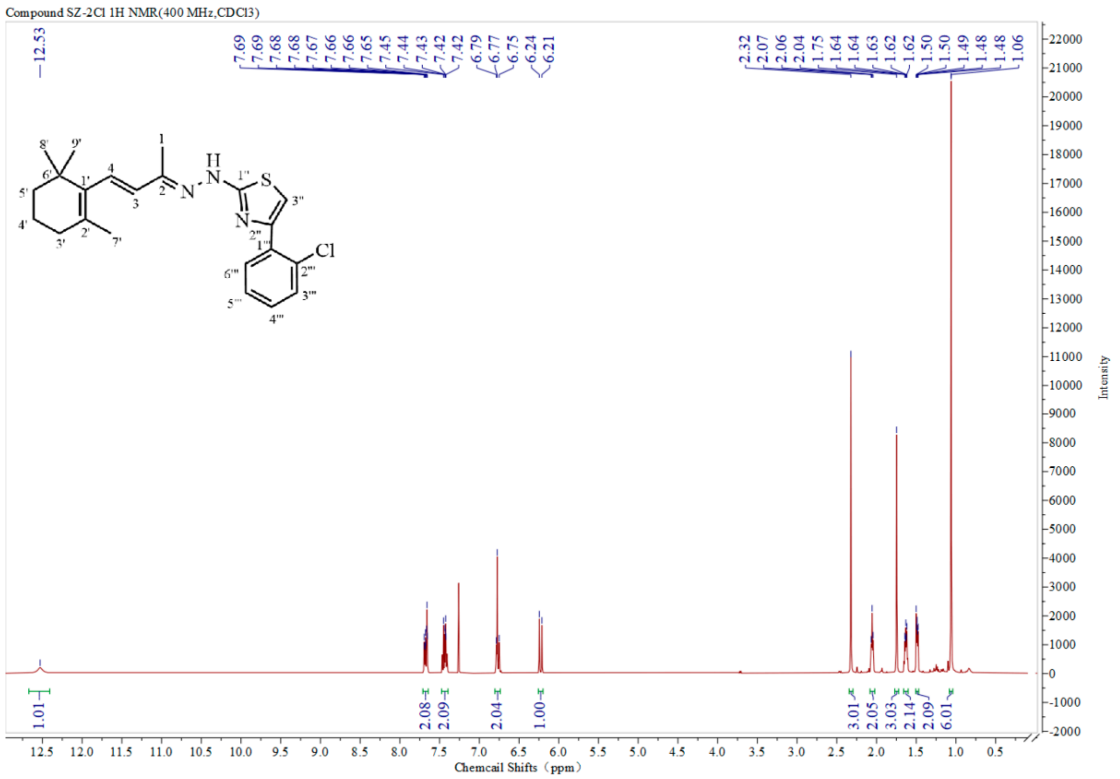

Figure S13. The ^{1}H NMR spectra Compound 1c

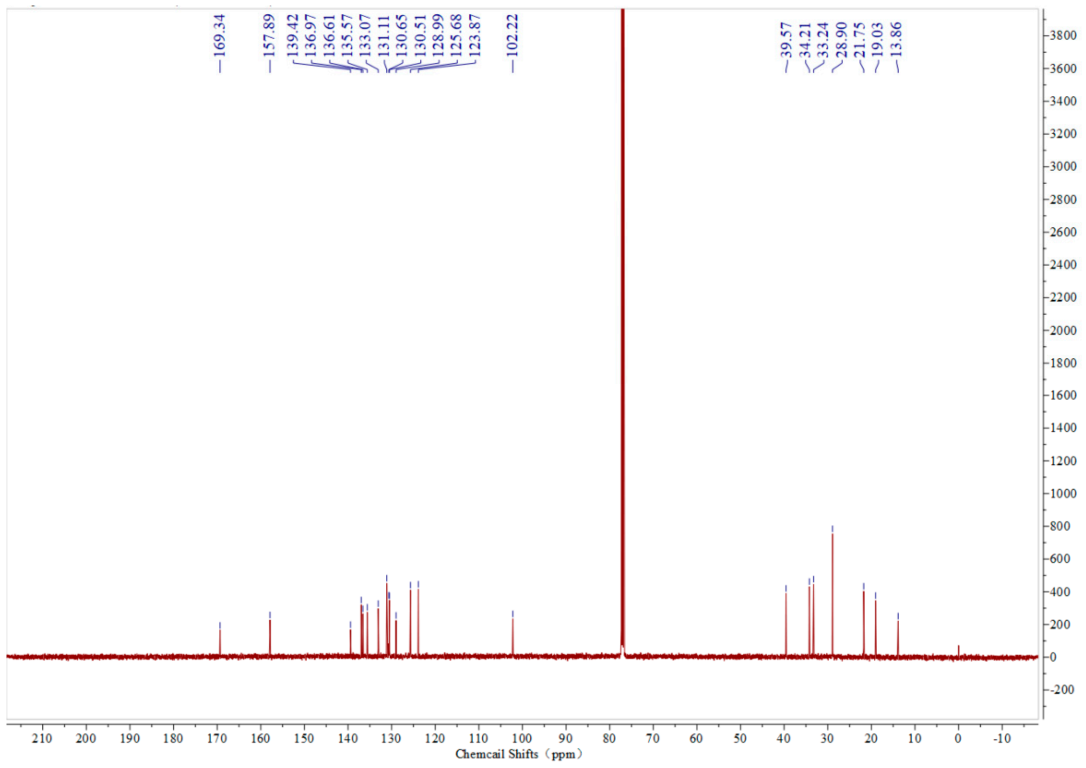

Figure S14. The ^{13}C NMR spectra of Compound 1c

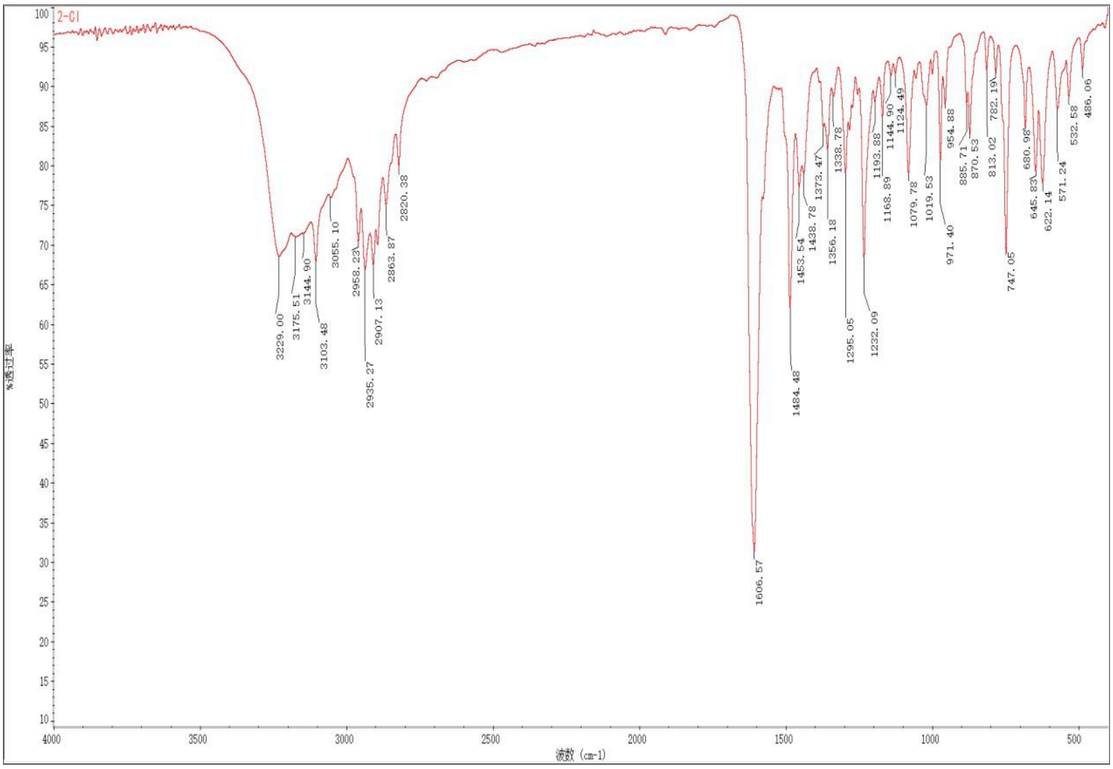

Figure S15. The ¹H NMR spectra Compound 1c

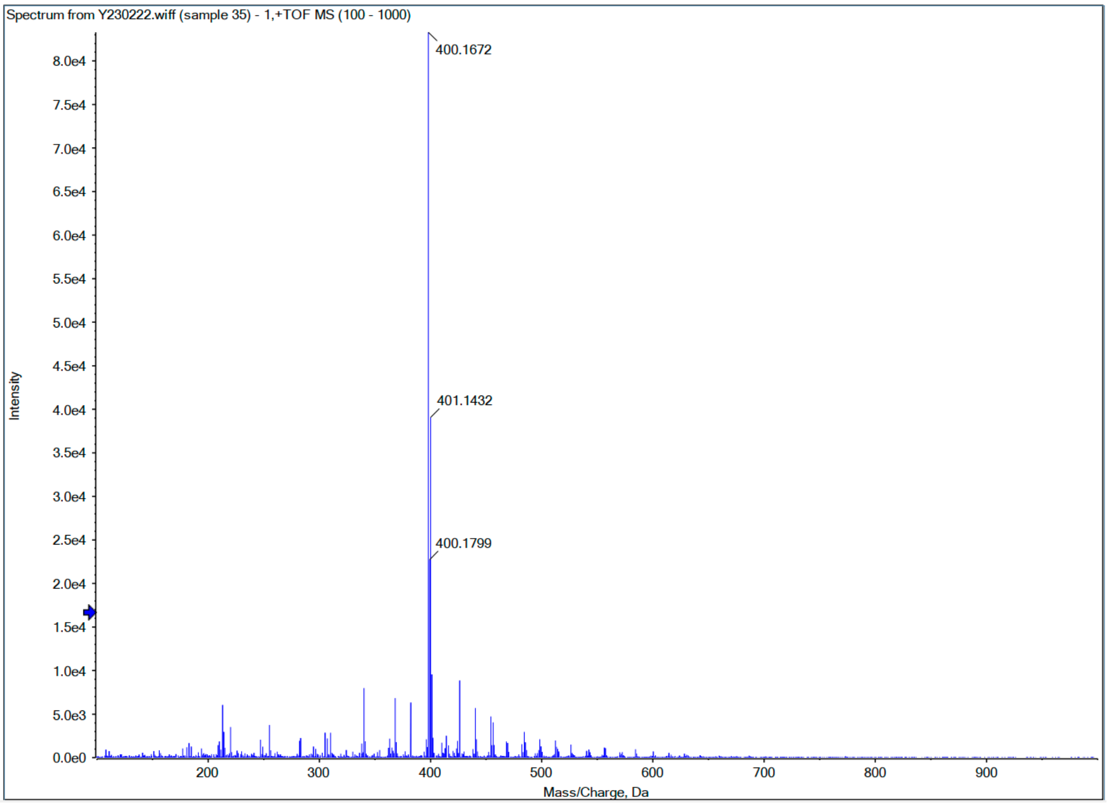

Figure S16. The HRMS spectra of Compound 1c

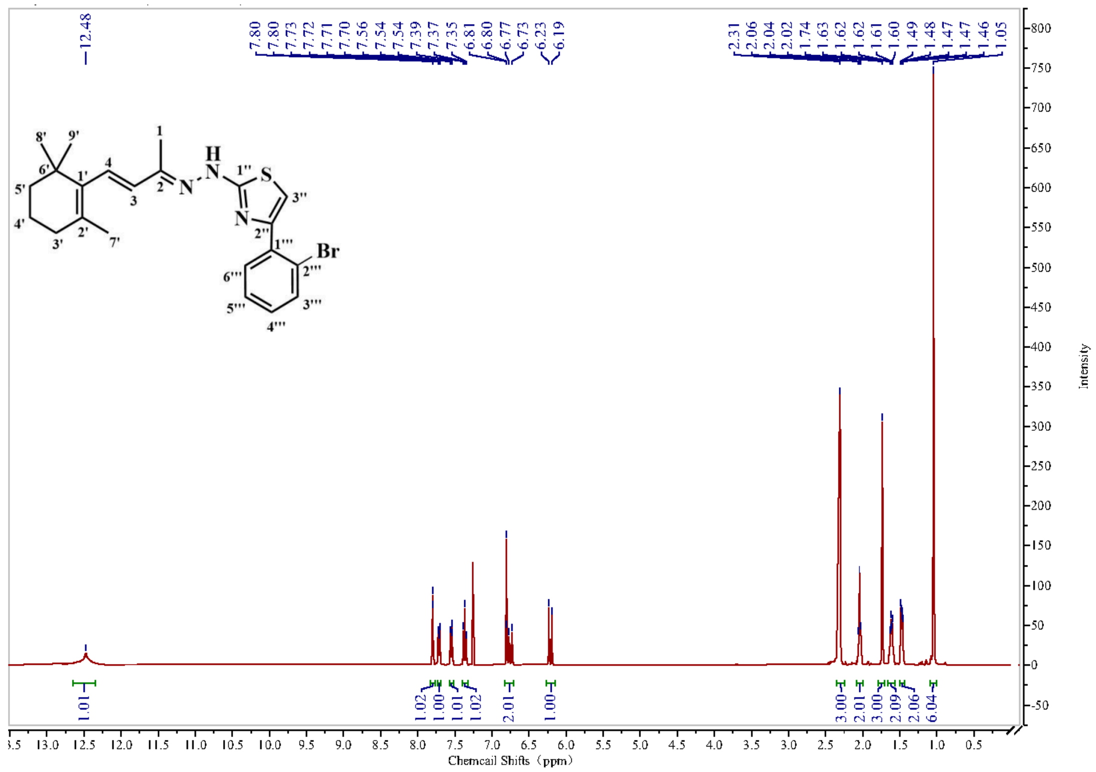

Figure S17. The ^{1}H NMR spectra Compound 1d

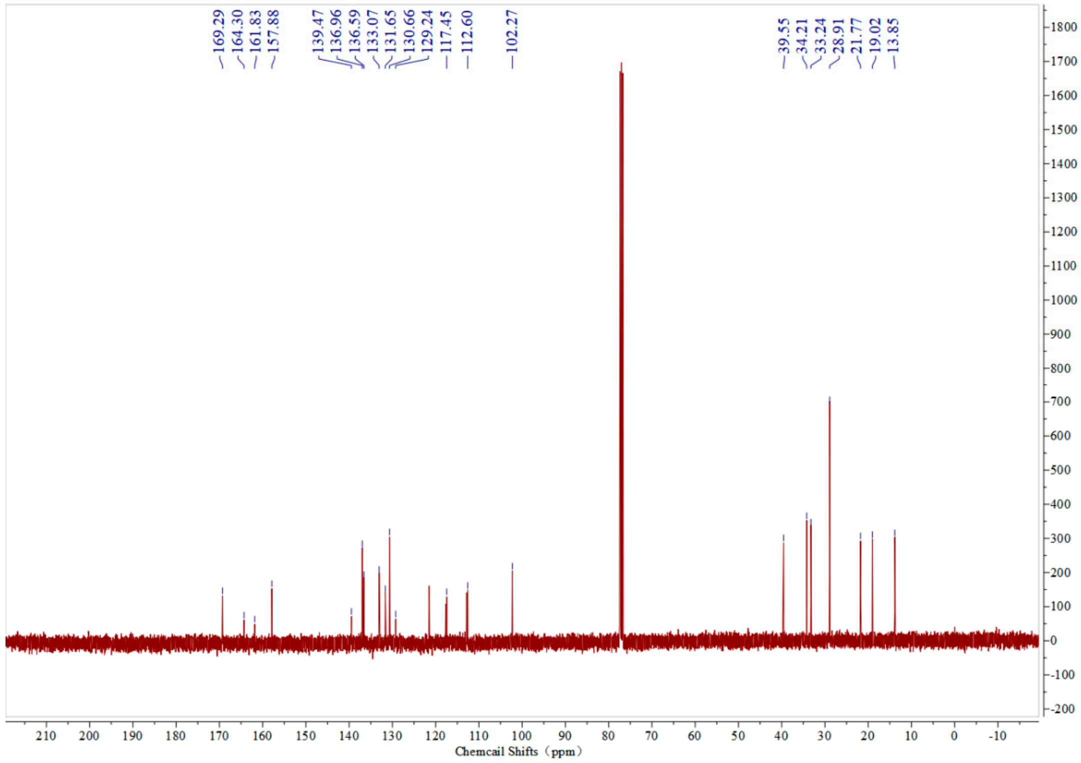

Figure S18. The ^{13}C NMR spectra of Compound 1d

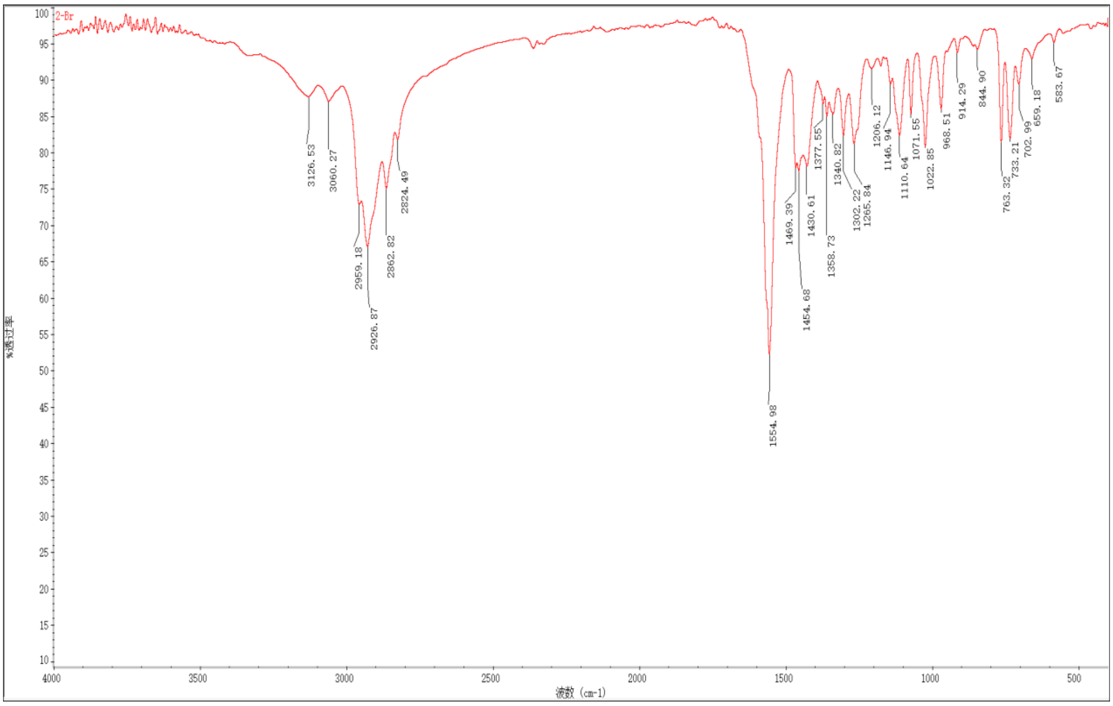

Figure S19. The FT-IR spectra of Compound 1d

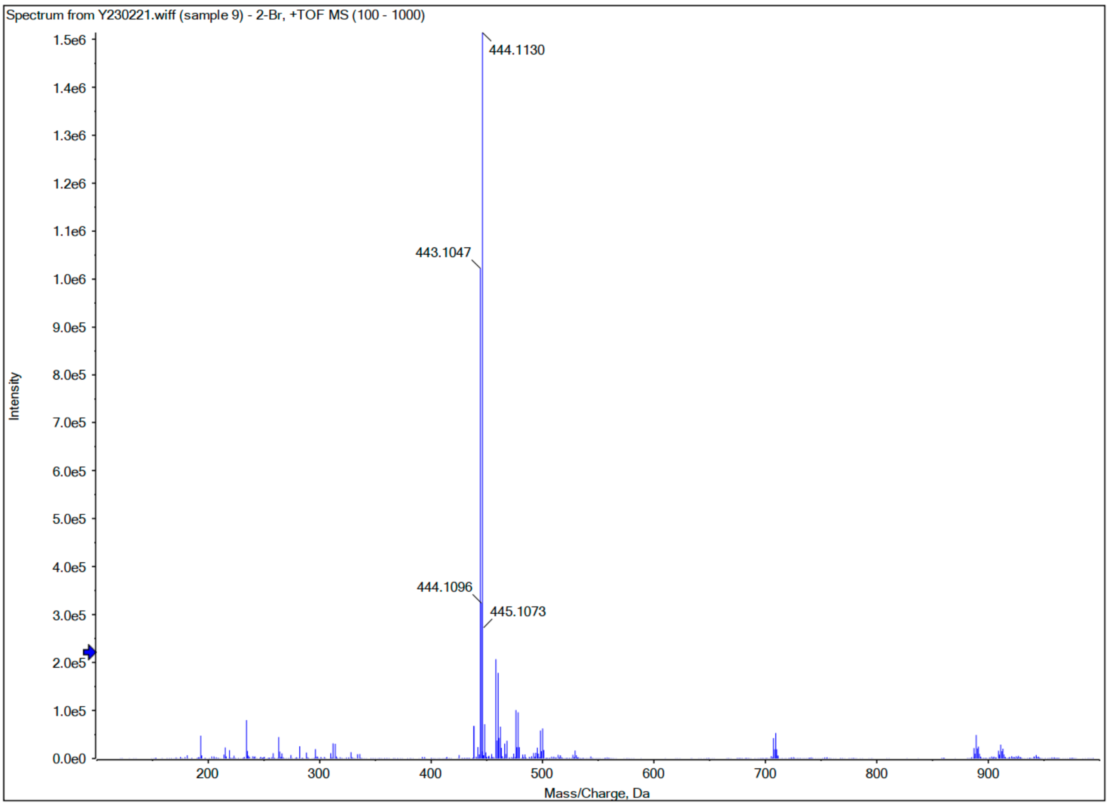

Figure S20. The HRMS spectra of Compound 1c

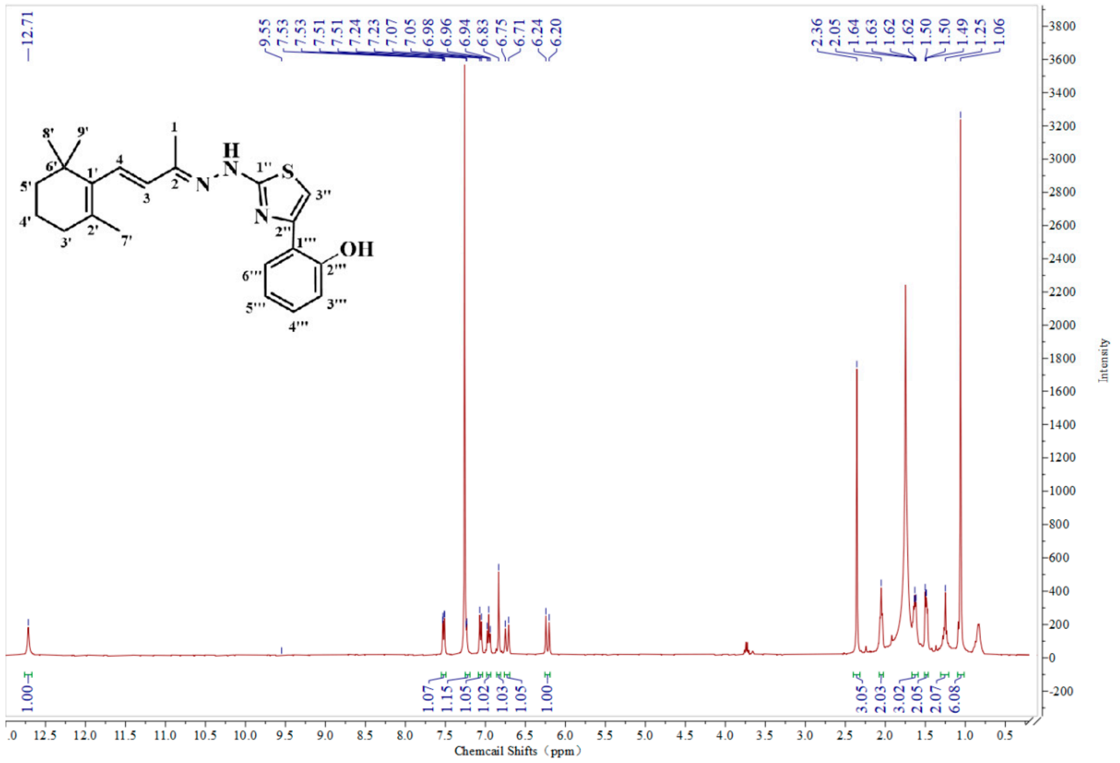

Figure S21.The ^{1}H NMR spectra Compound 1e

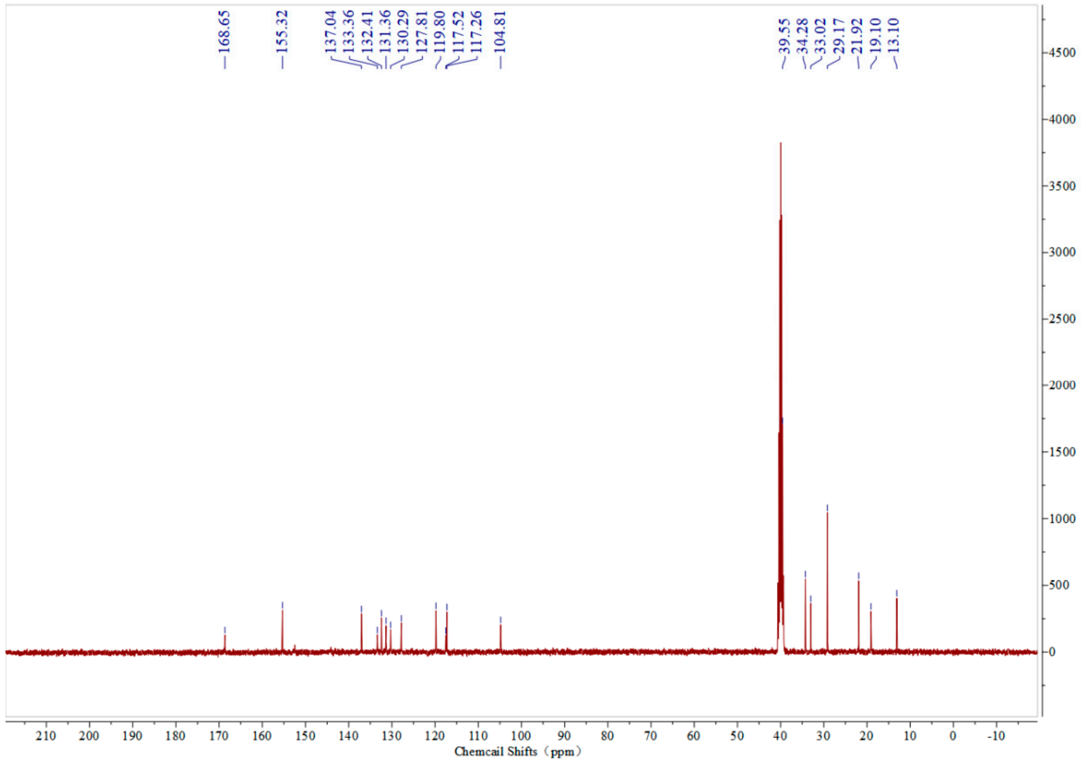

Figure S22.The ^{13}C NMR spectra of Compound 1e

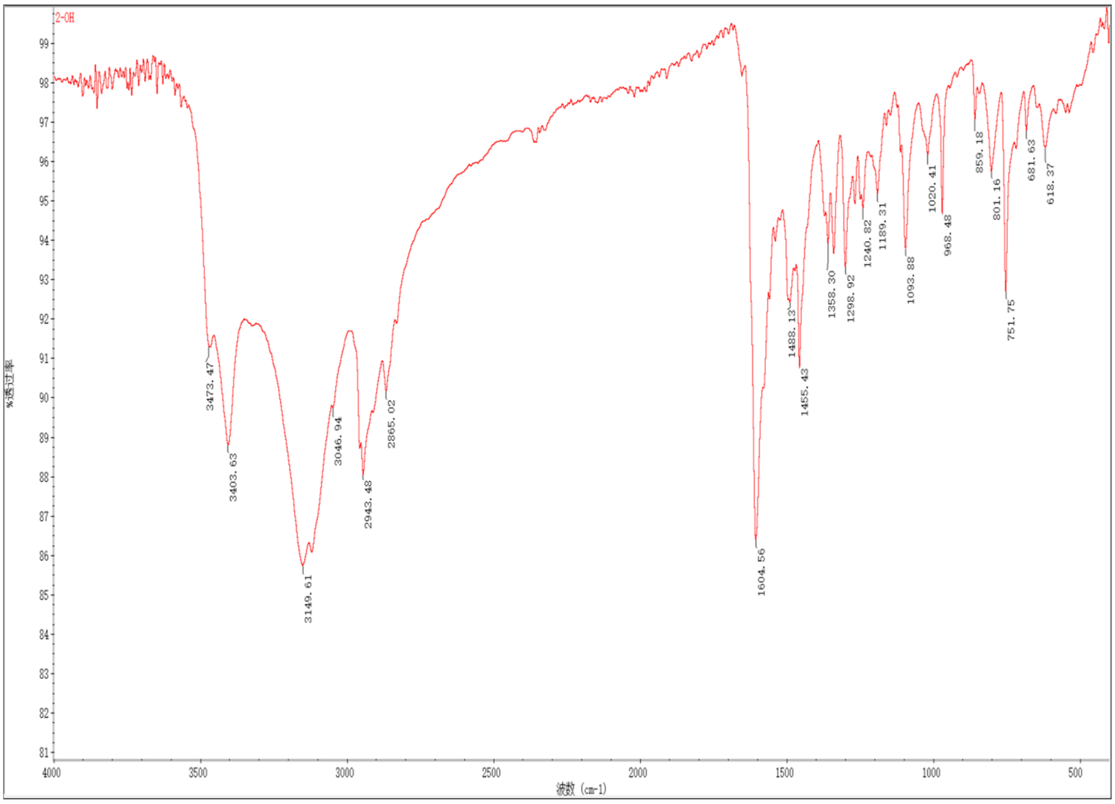

Figure S23.The FT-IR spectra of Compound 1e

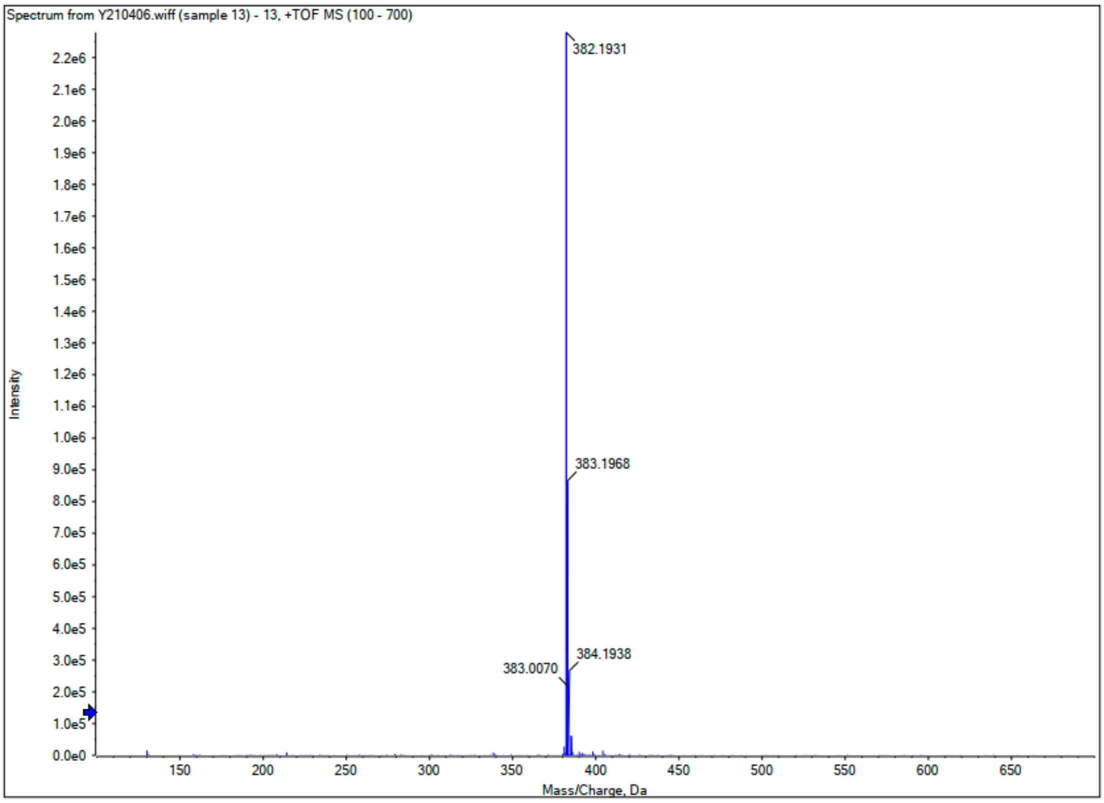

Figure S24.The HRMS spectra of Compound 1e

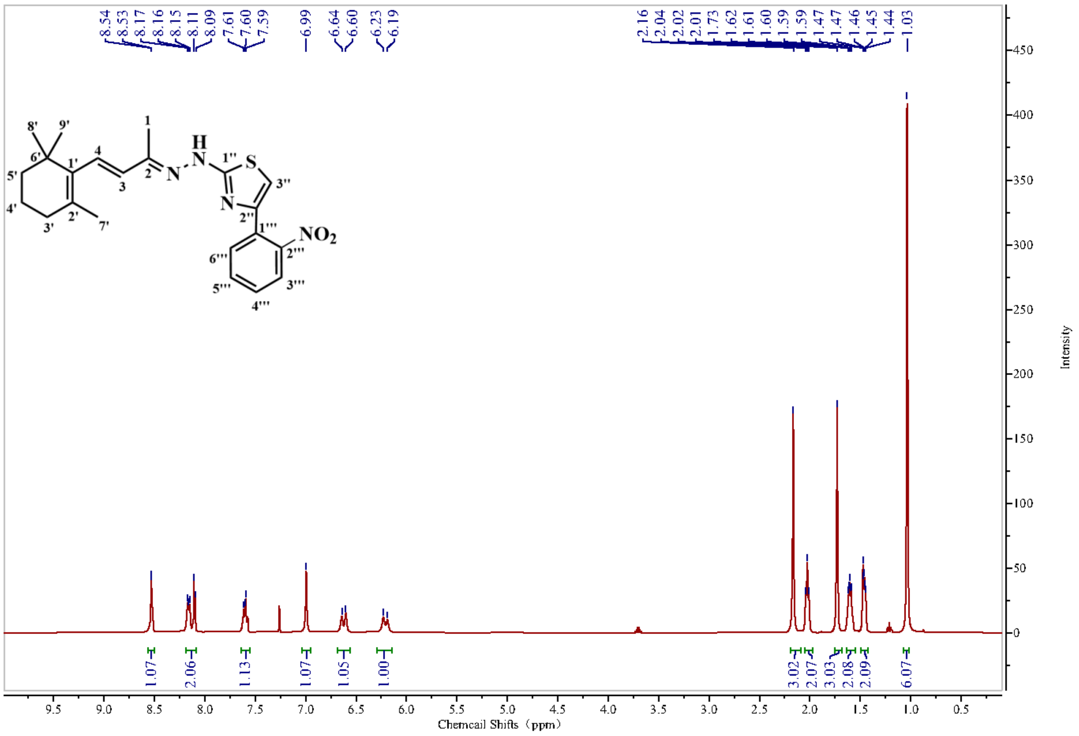

Figure S25.The ^{1}H NMR spectra Compound 1f

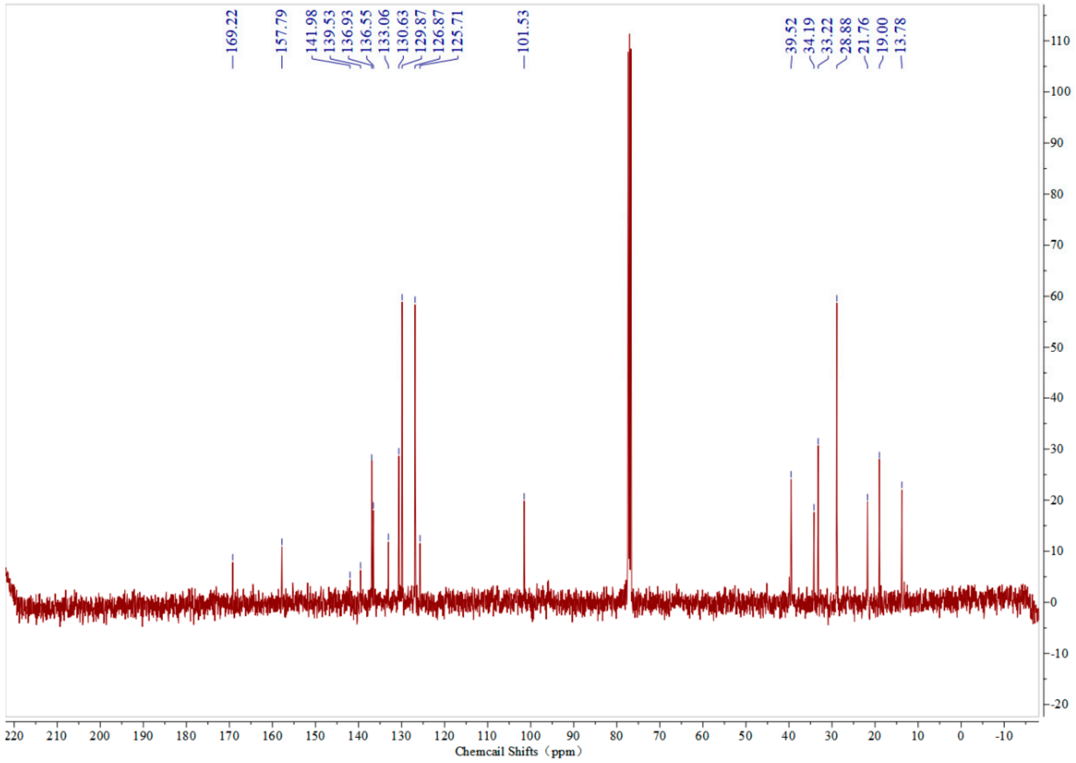

Figure S26.The ^{13}C NMR spectra of Compound 1f

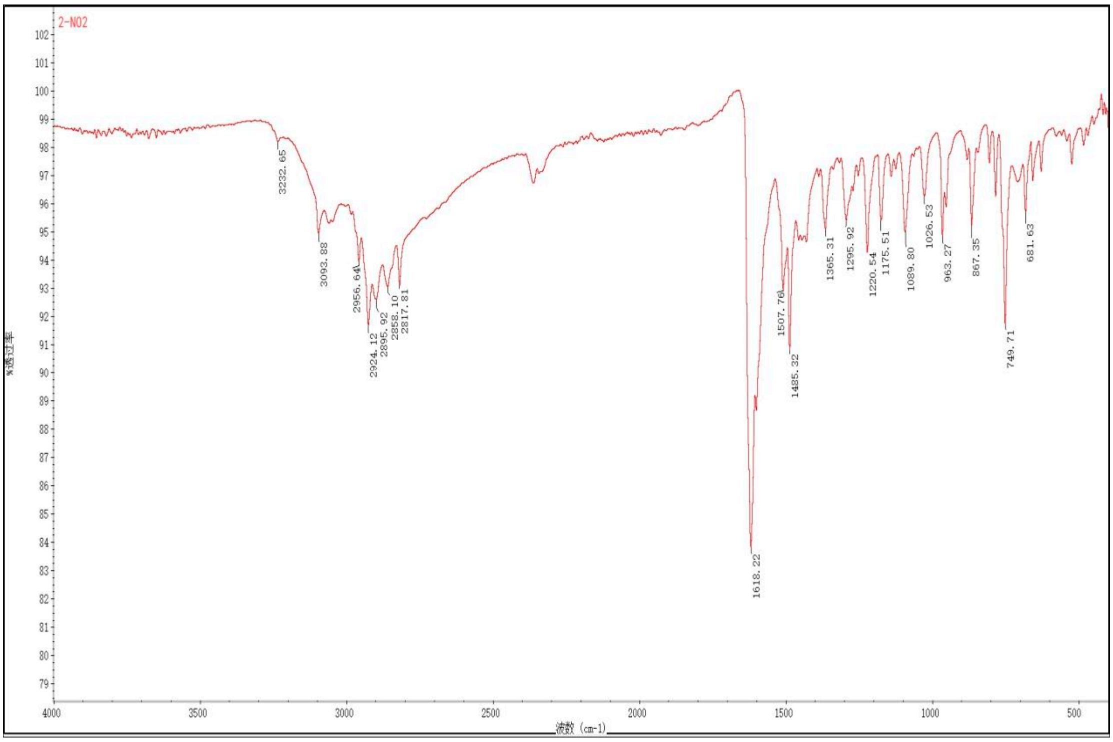

Figure S27.The FT-IR spectra of Compound 1f

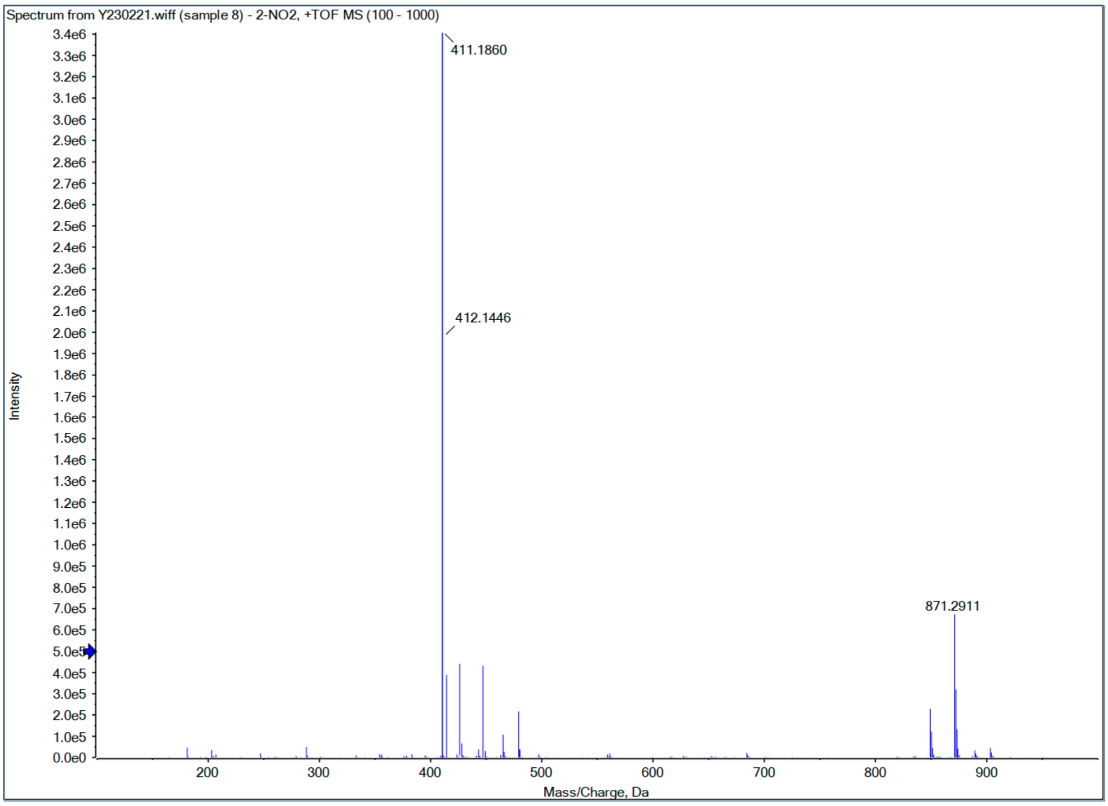

Figure S28.The HRMS spectra of Compound 1f

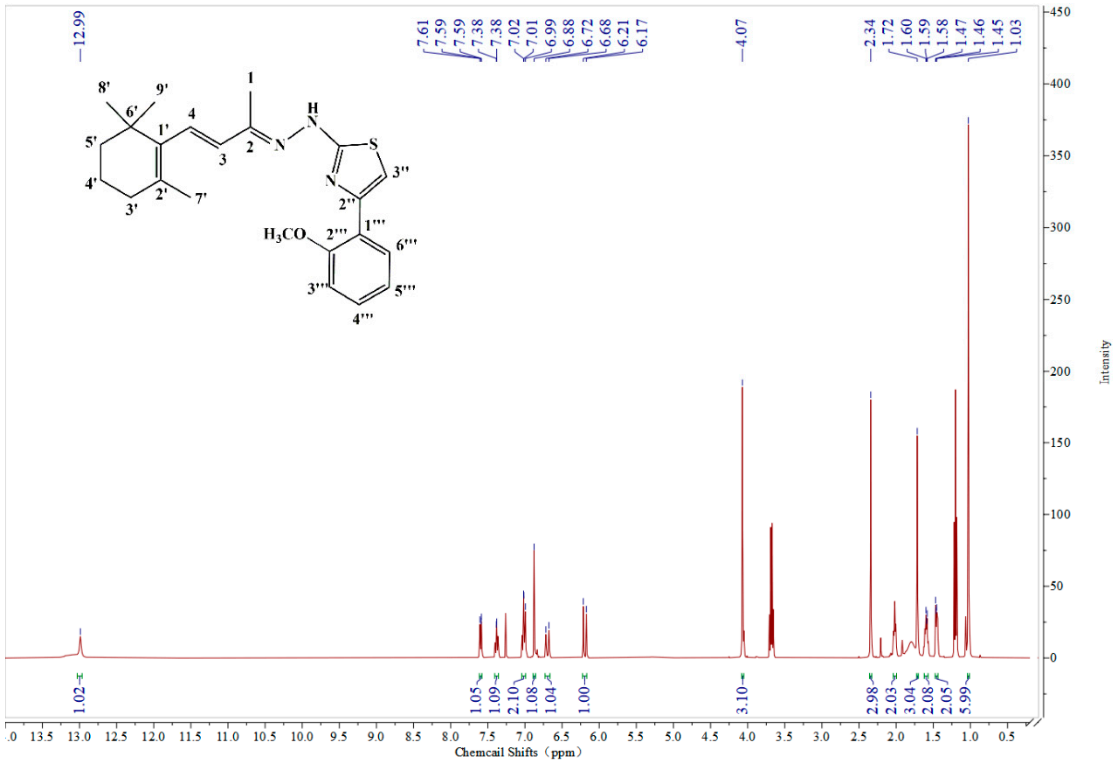

Figure S29.The ^{1}H NMR spectra Compound 1g

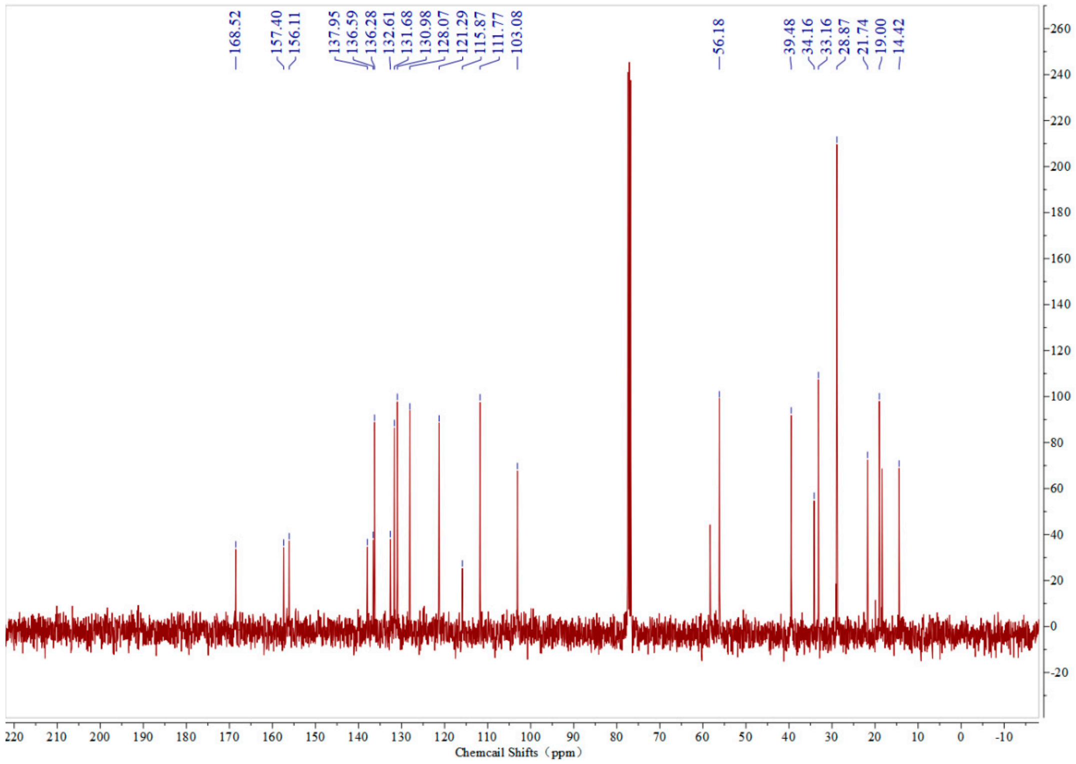

Figure S30. The ^{13}C NMR spectra of Compound 1g

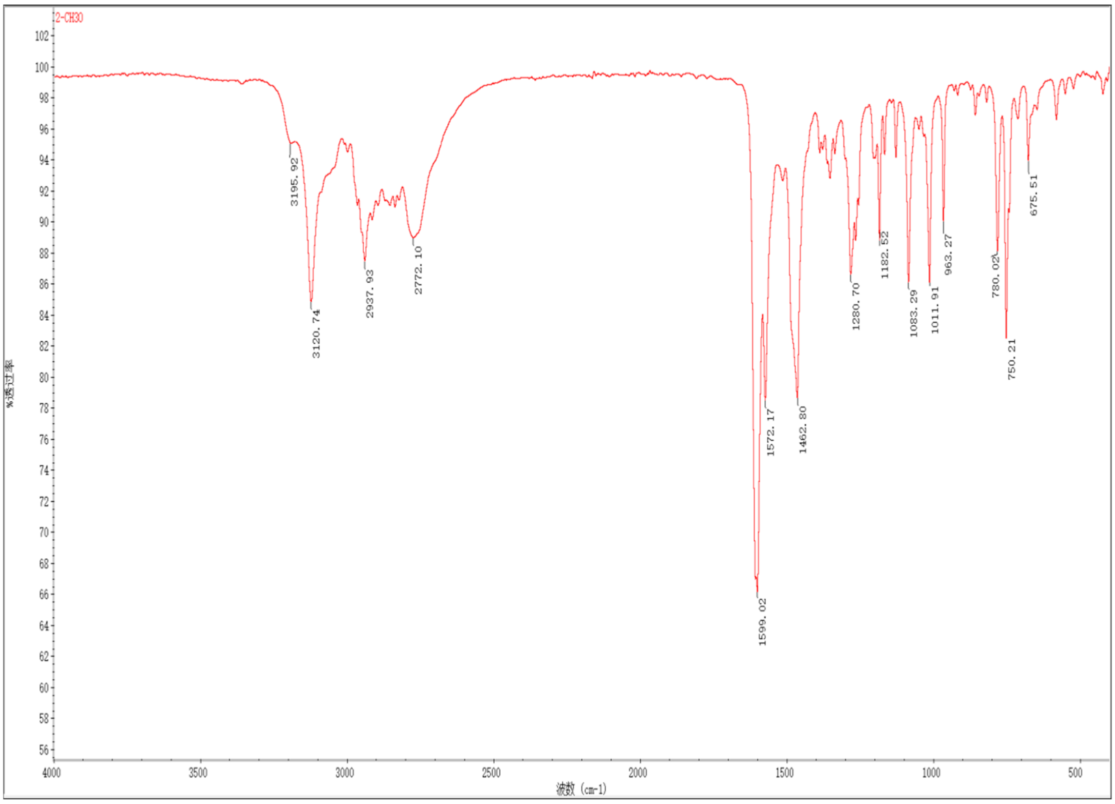

Figure S31. The FT-IR spectra of Compound 1g

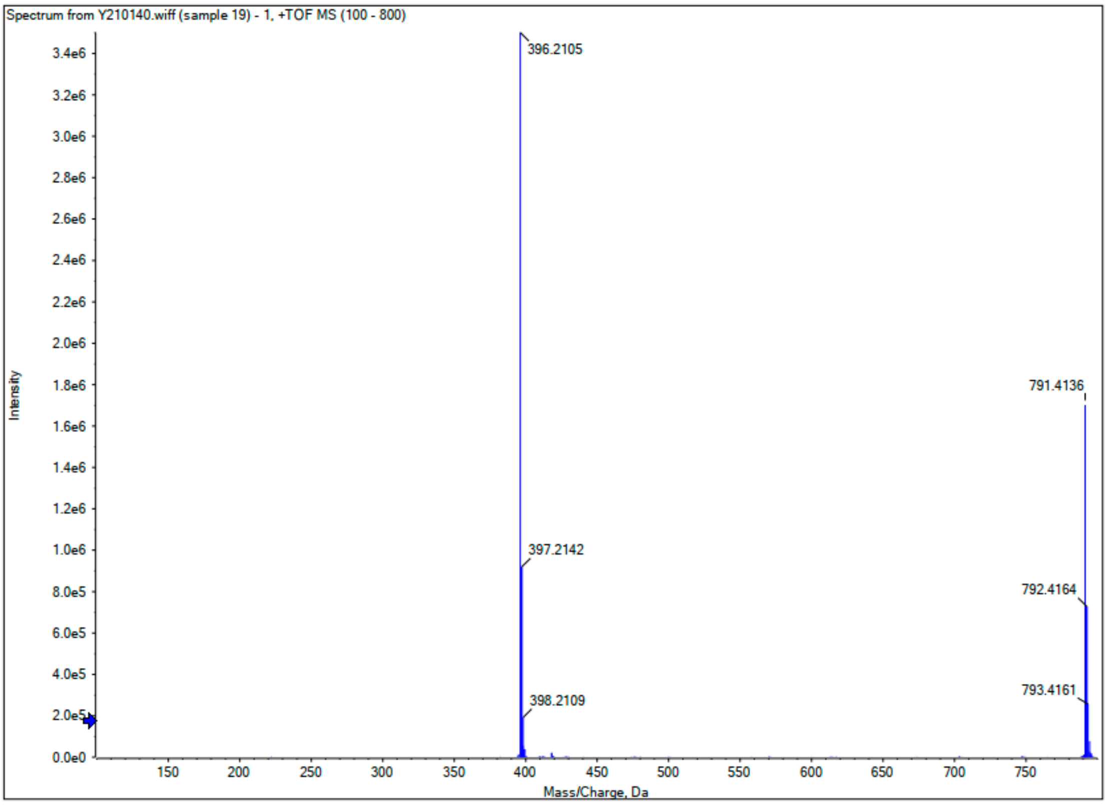

Figure S32. The HRMS spectra of Compound 1g

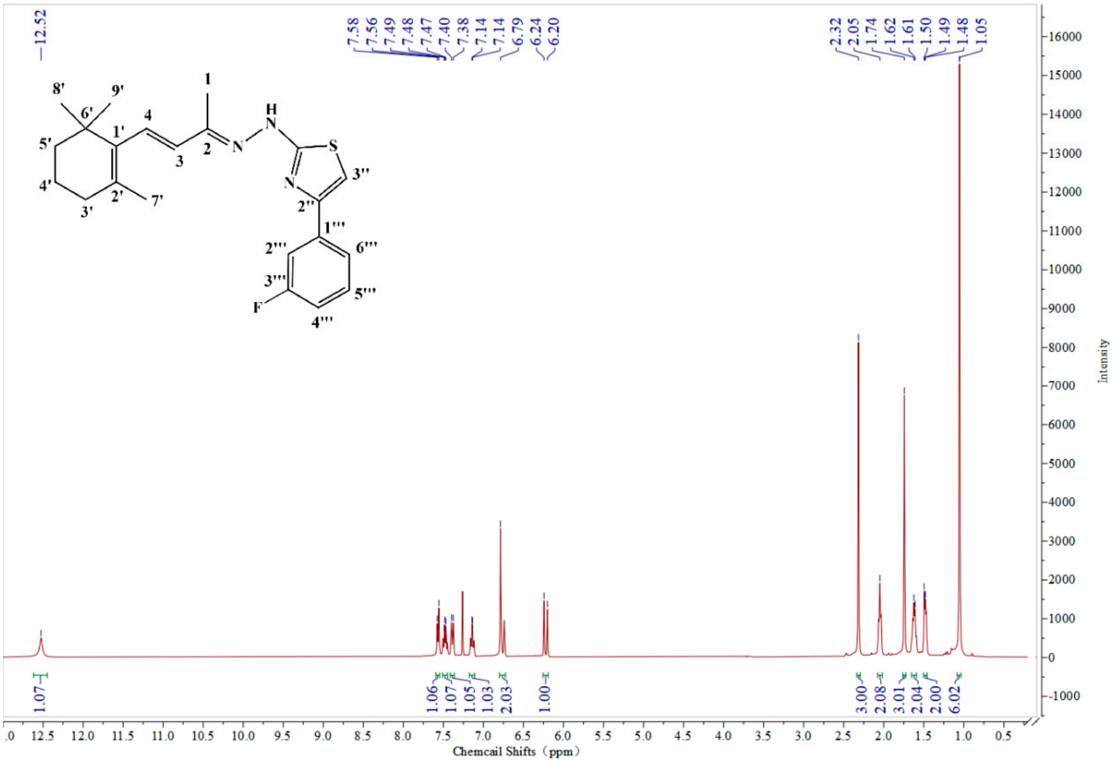

Figure S33. The ^{1}H NMR spectra Compound 1h

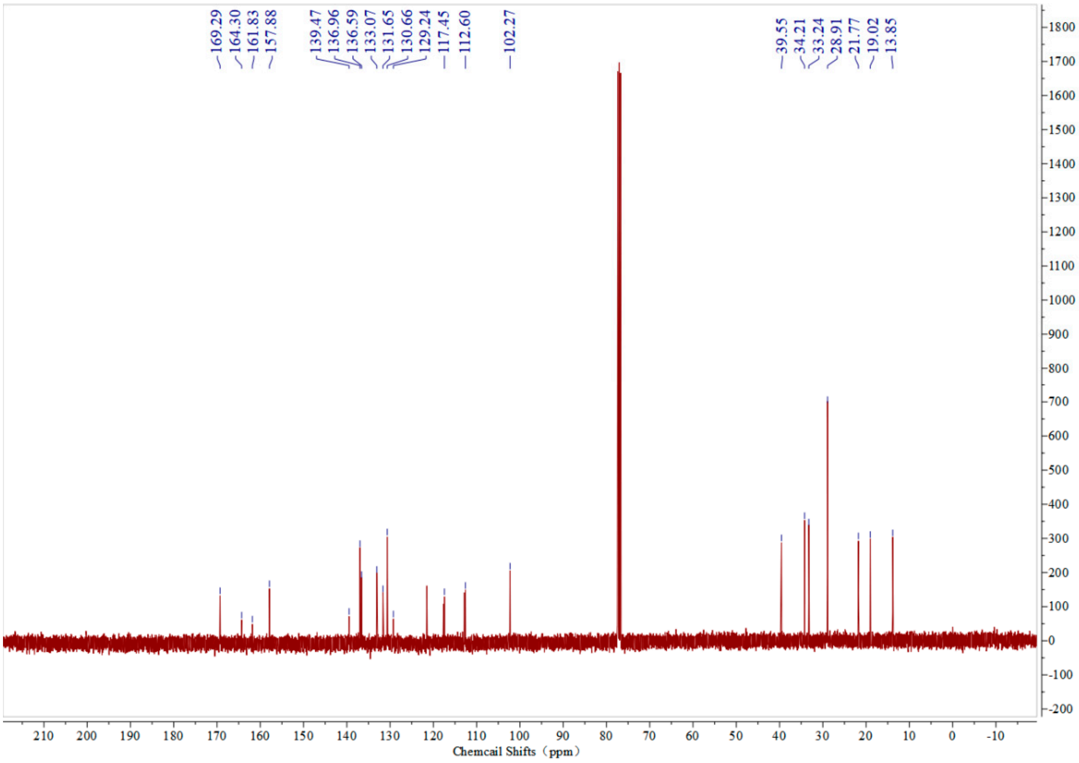

Figure S34. The ^{13}C NMR spectra of Compound 1h

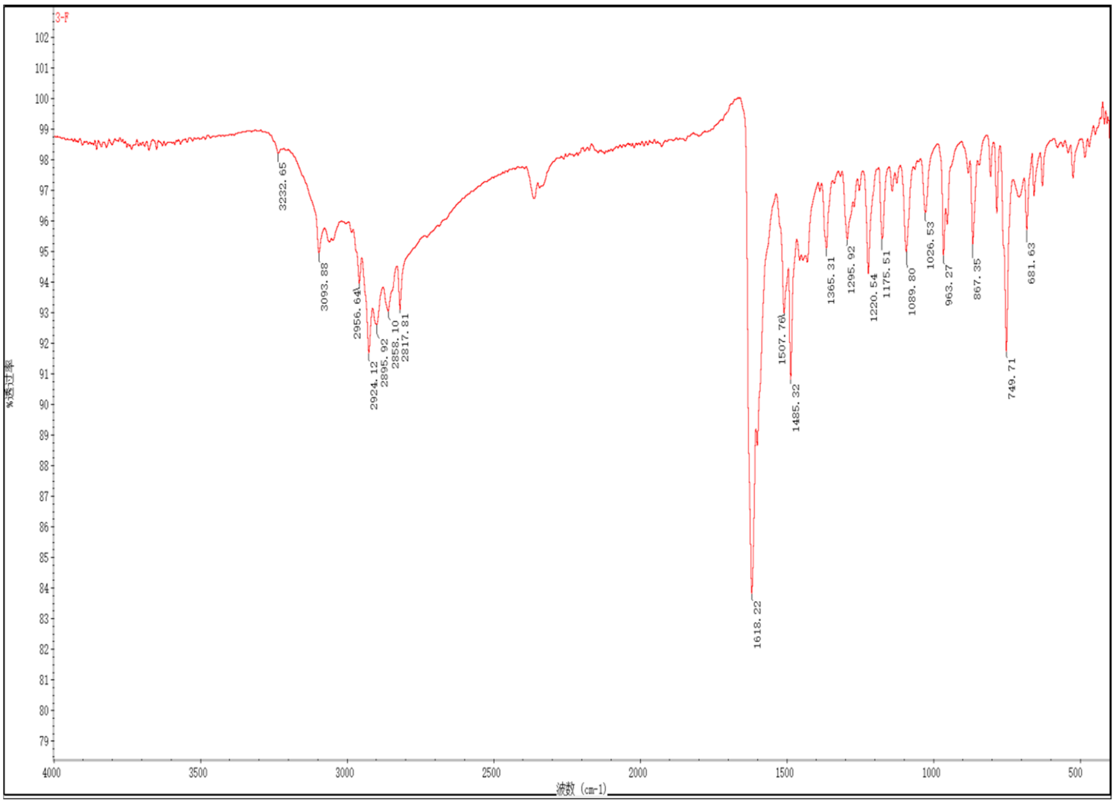

Figure S35. The FT-IR spectra of Compound 1h

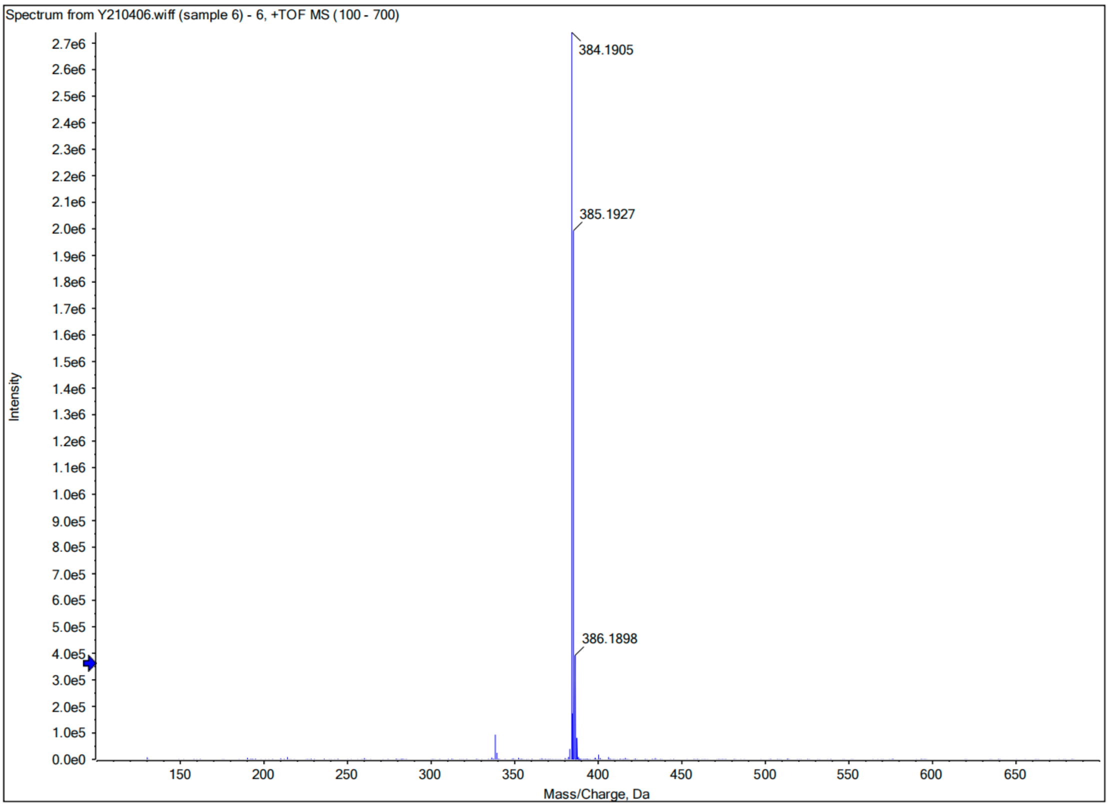

Figure S36. The HRMS spectra of Compound 1h

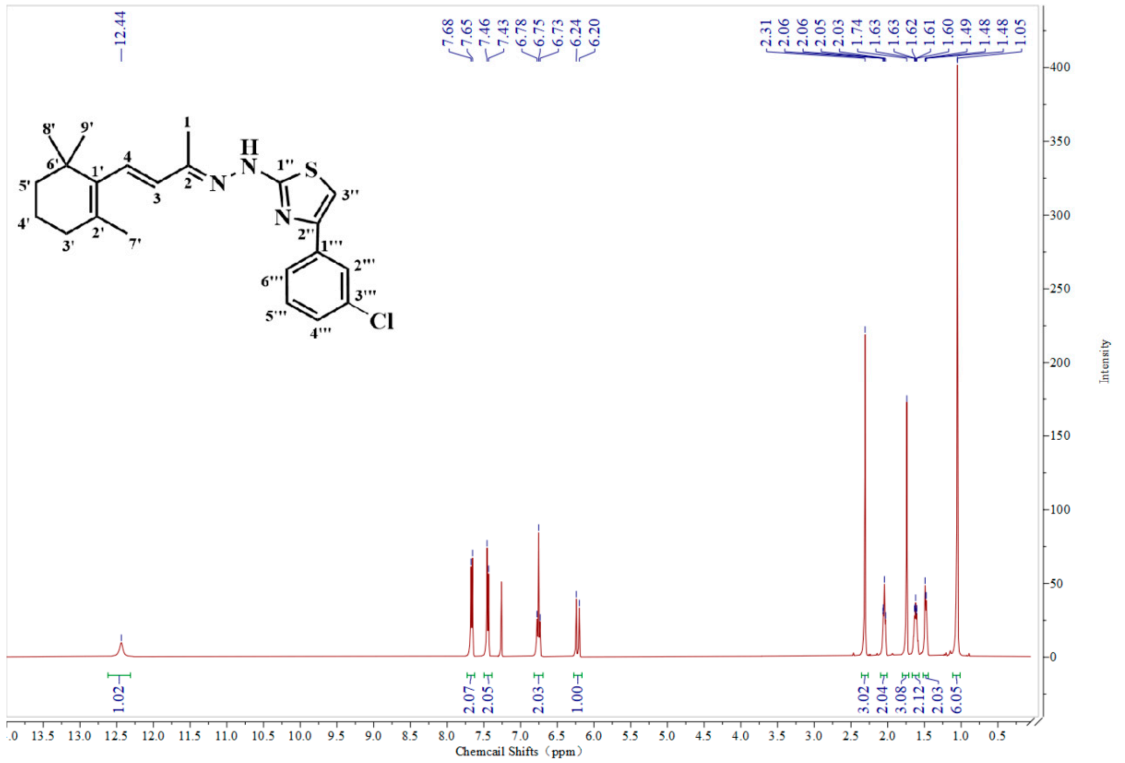

Figure S37. The ^{1}H NMR spectra Compound 1i

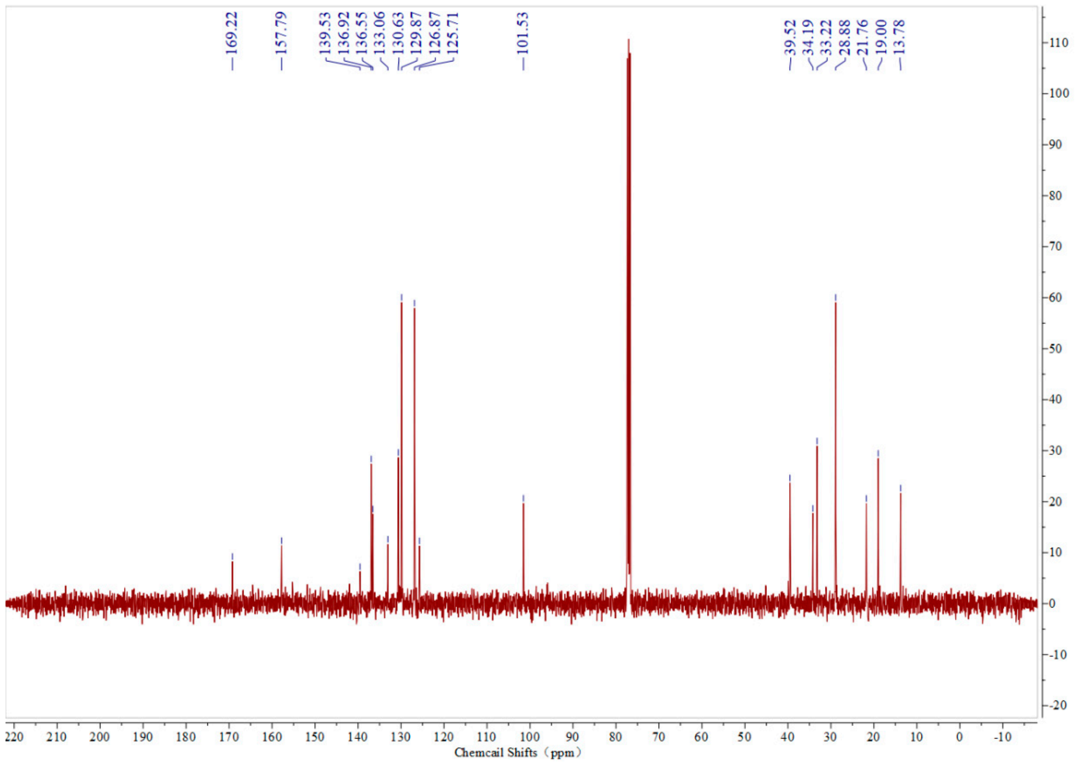

Figure S38. The ^{13}C NMR spectra of Compound 1i

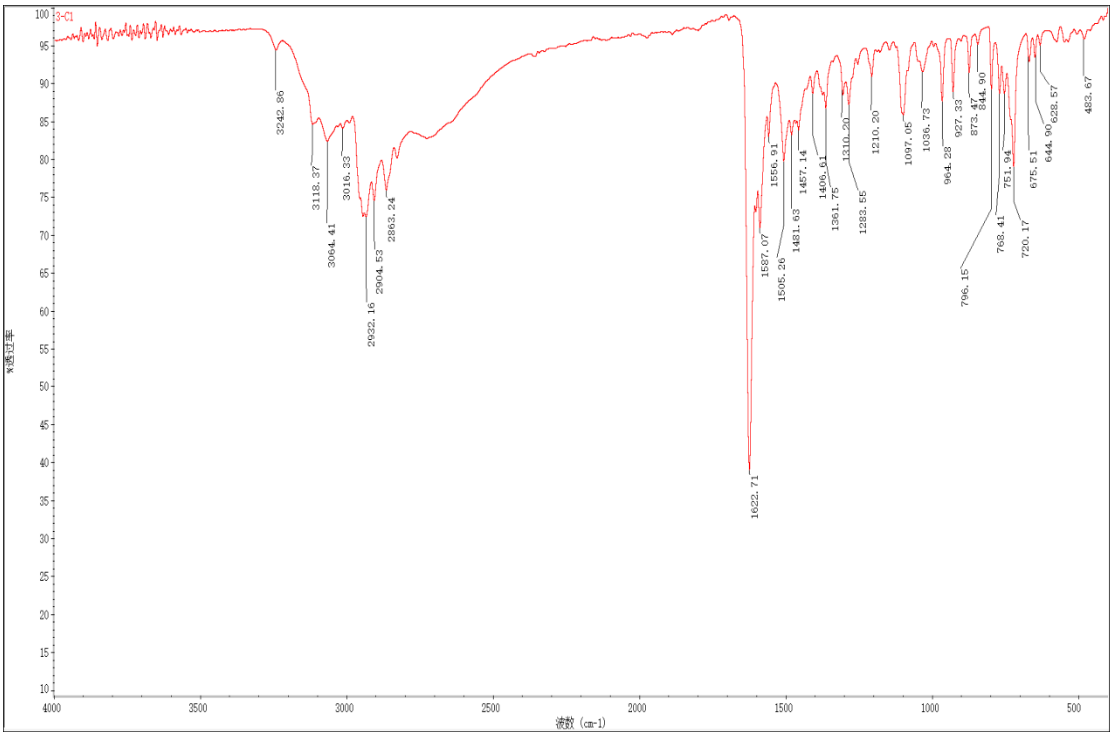

Figure S39. The FT-IR spectra of Compound 1i

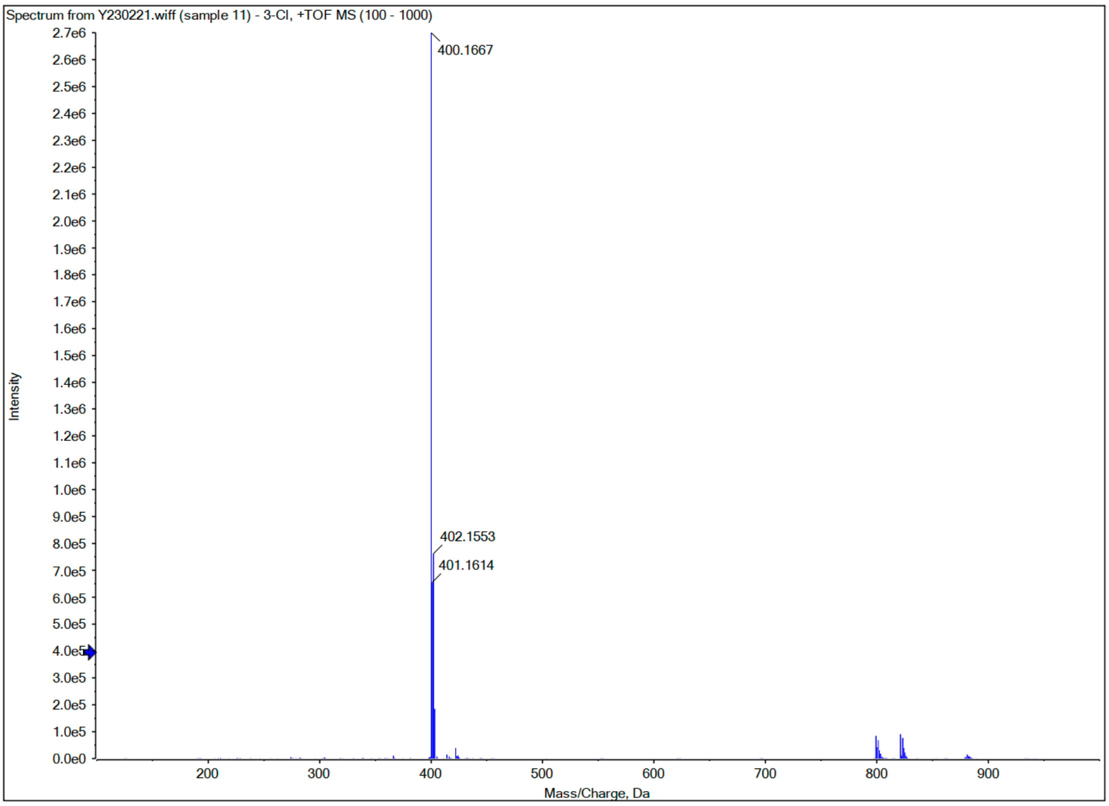

Figure S40. The HRMS spectra of Compound 1i

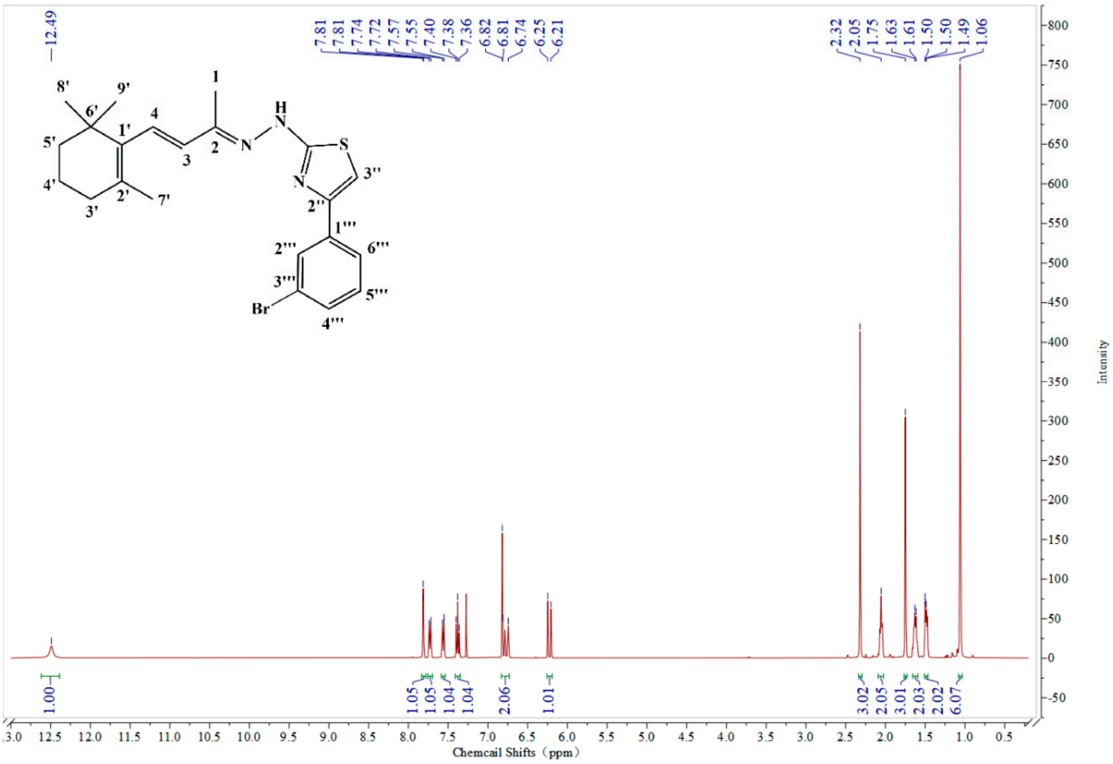

Figure S41. The ^{1}H NMR spectra Compound 1j

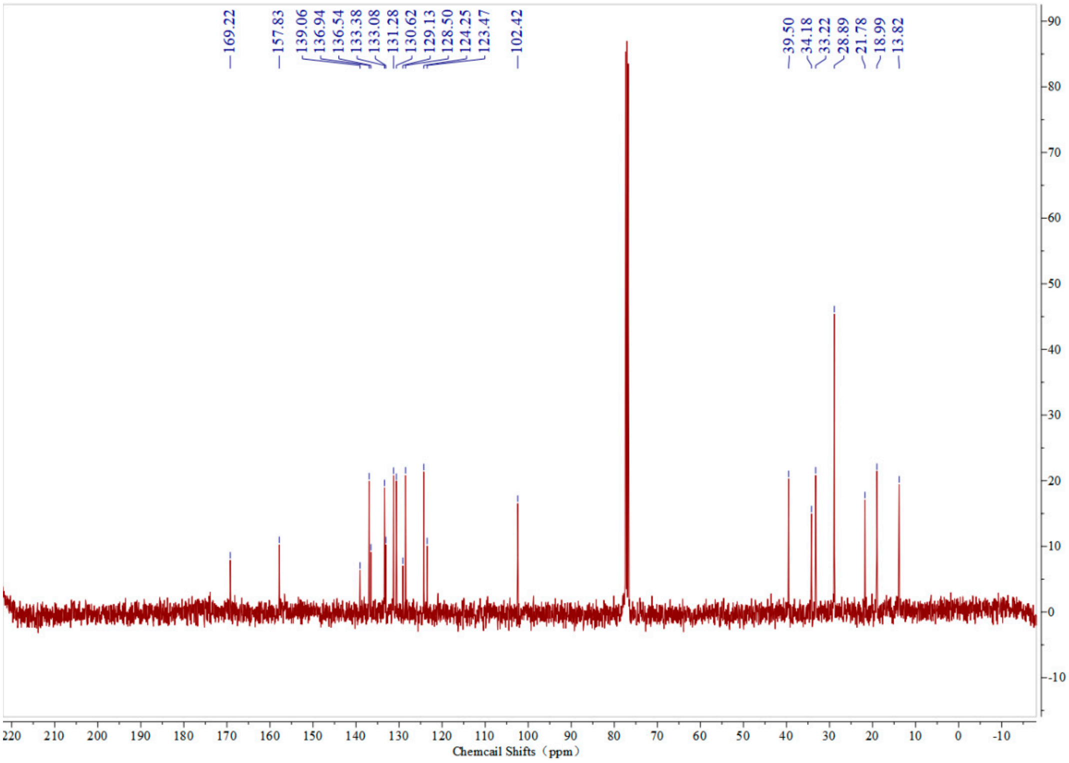

Figure S42. The ^{13}C NMR spectra of Compound 1j

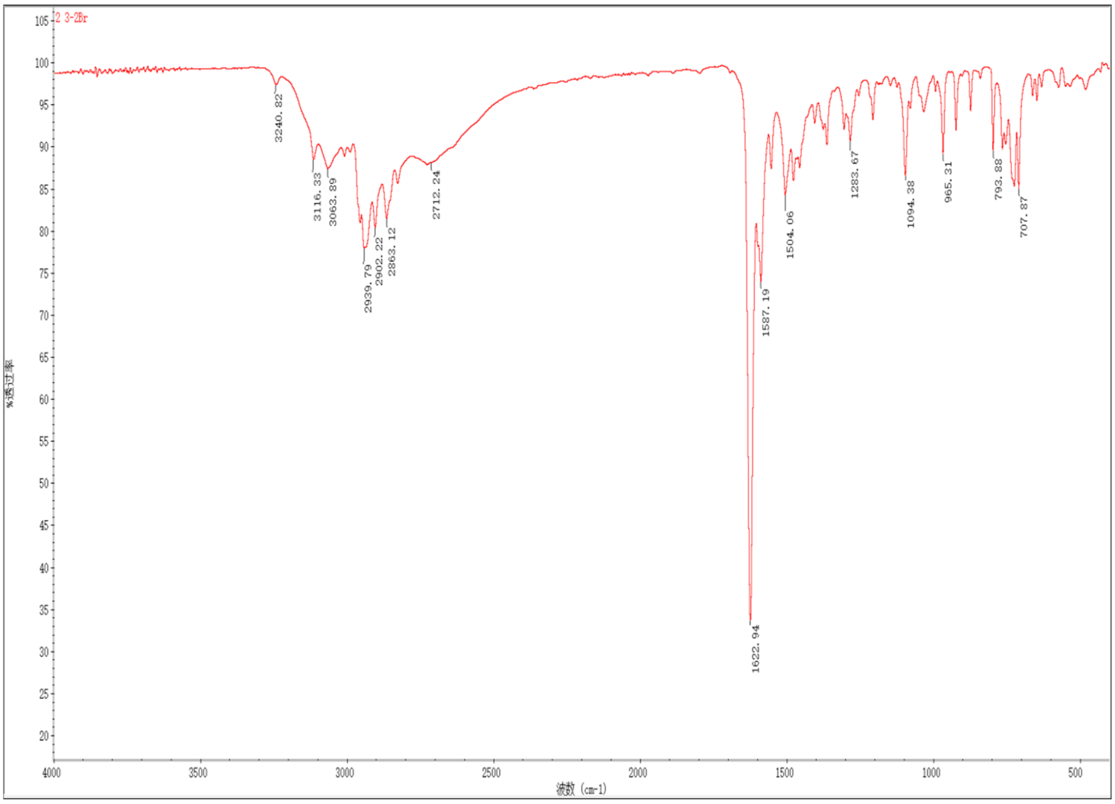

Figure S43. The FT-IR spectra of Compound 1j

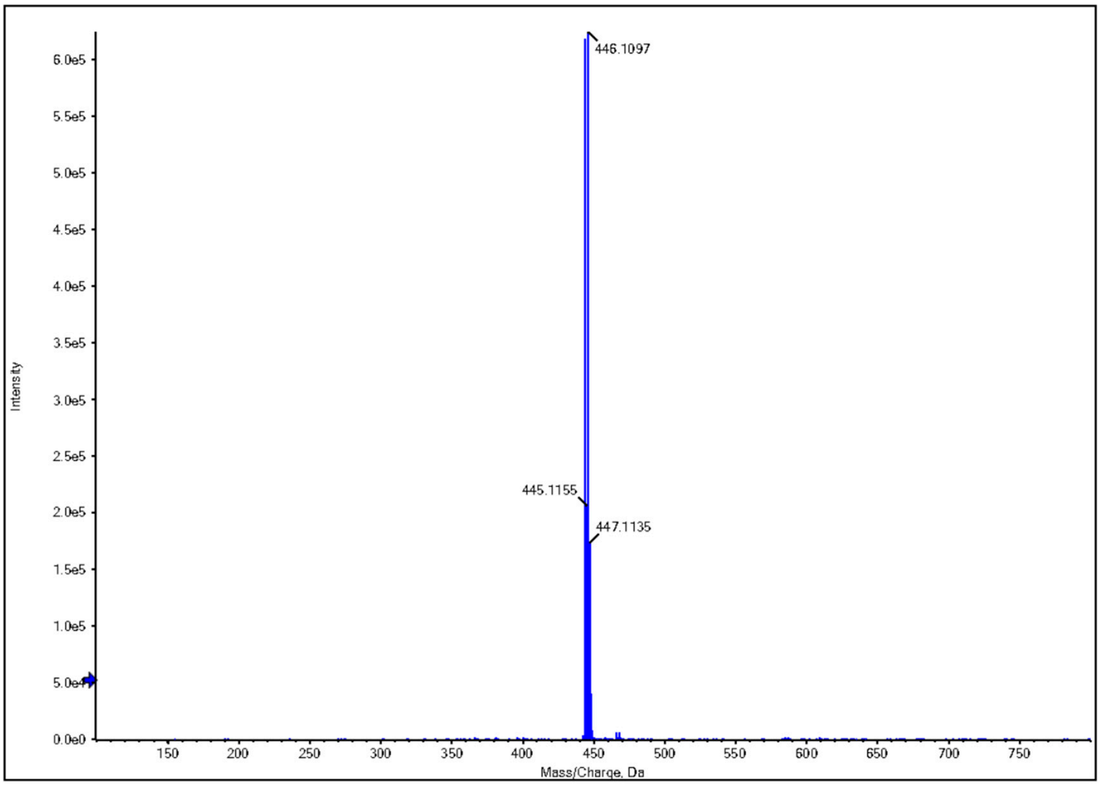

Figure S44. The HRMS spectra of Compound 1j

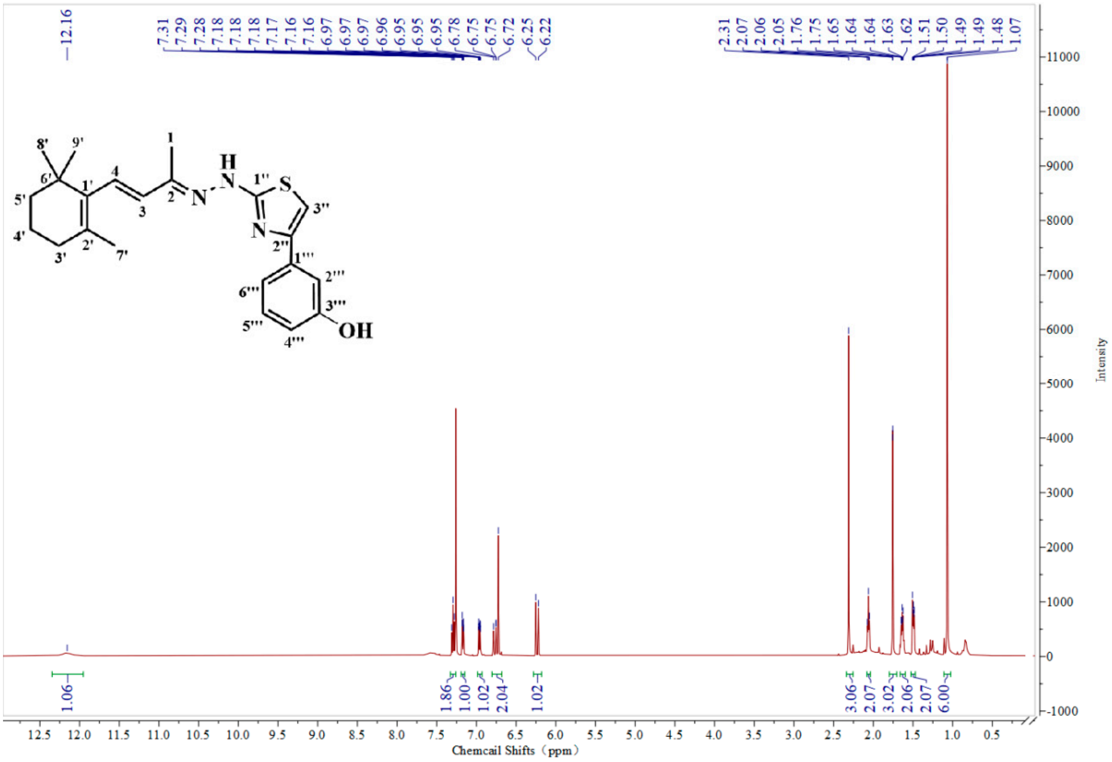

Figure S45. The ^{1}H NMR spectra Compound 1k

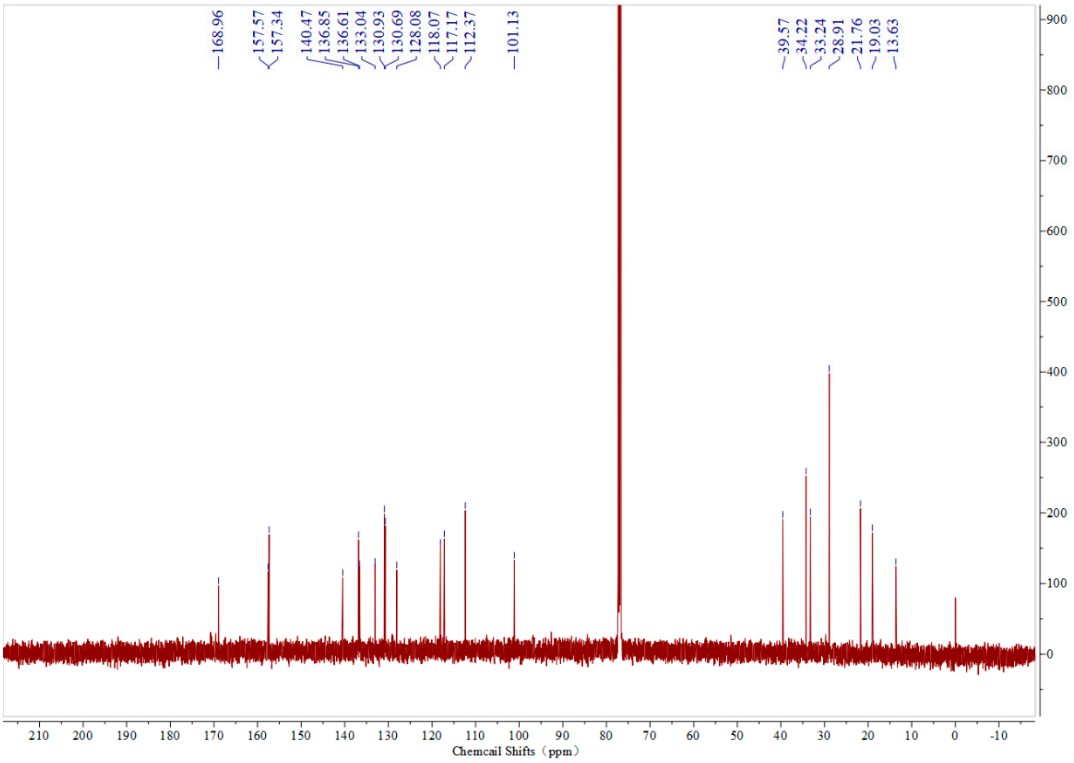

Figure S46. The ^{13}C NMR spectra of Compound 1k

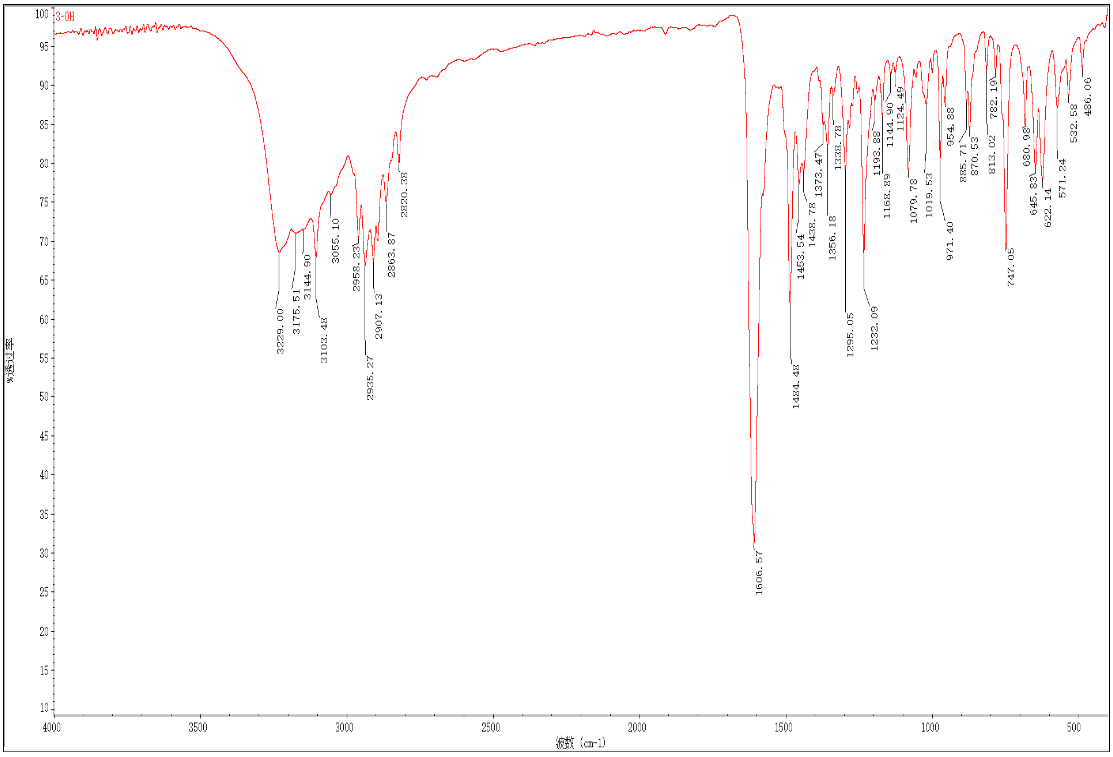

Figure S47. The FT-IR spectra of Compound 1k

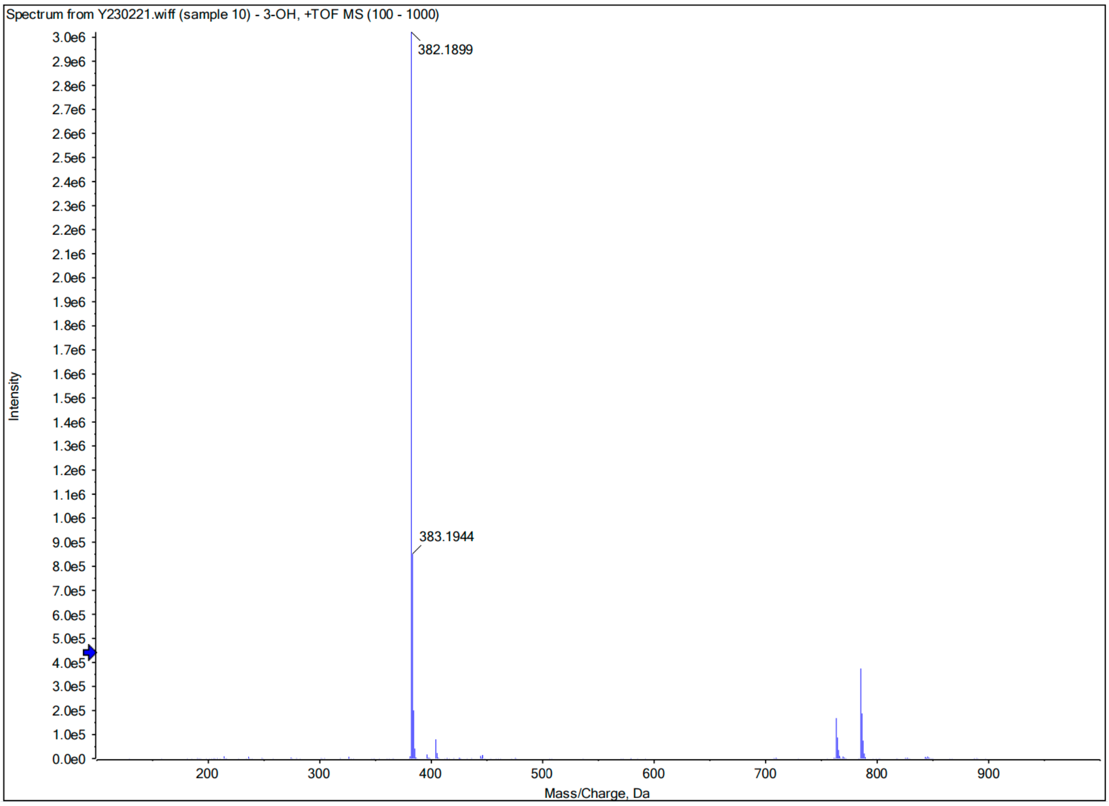

Figure S48. The HRMS spectra of Compound 1k

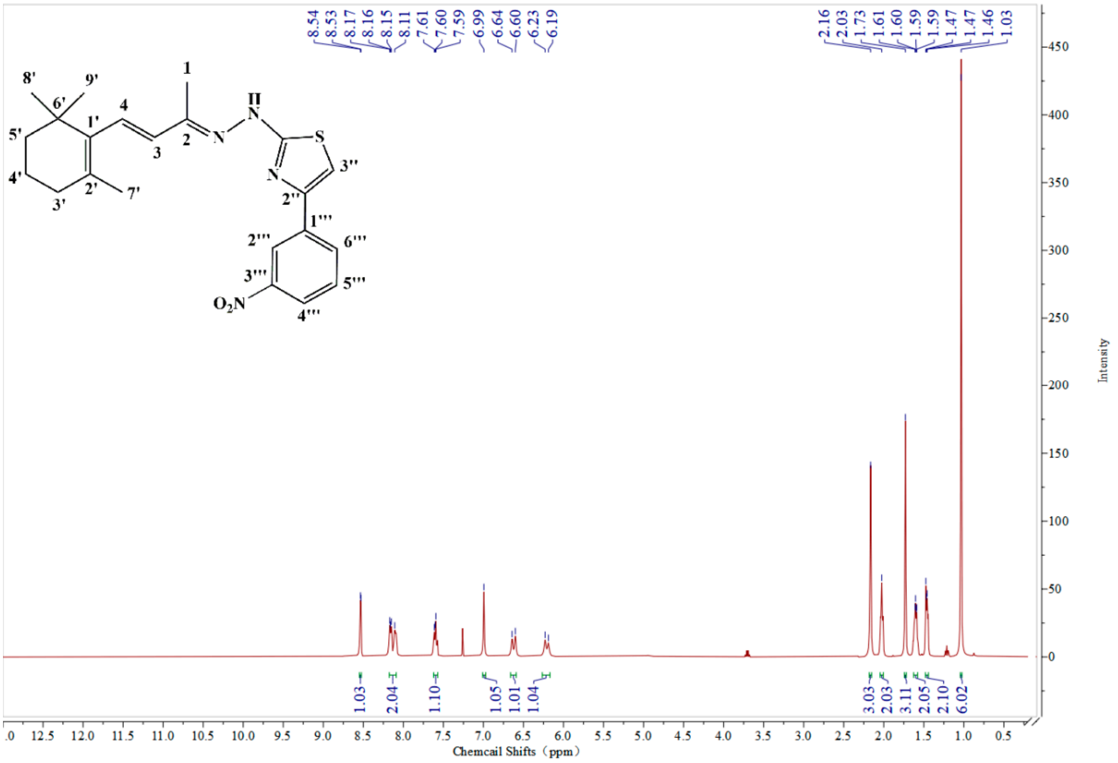

Figure S49. The ^{1}H NMR spectra Compound 1l

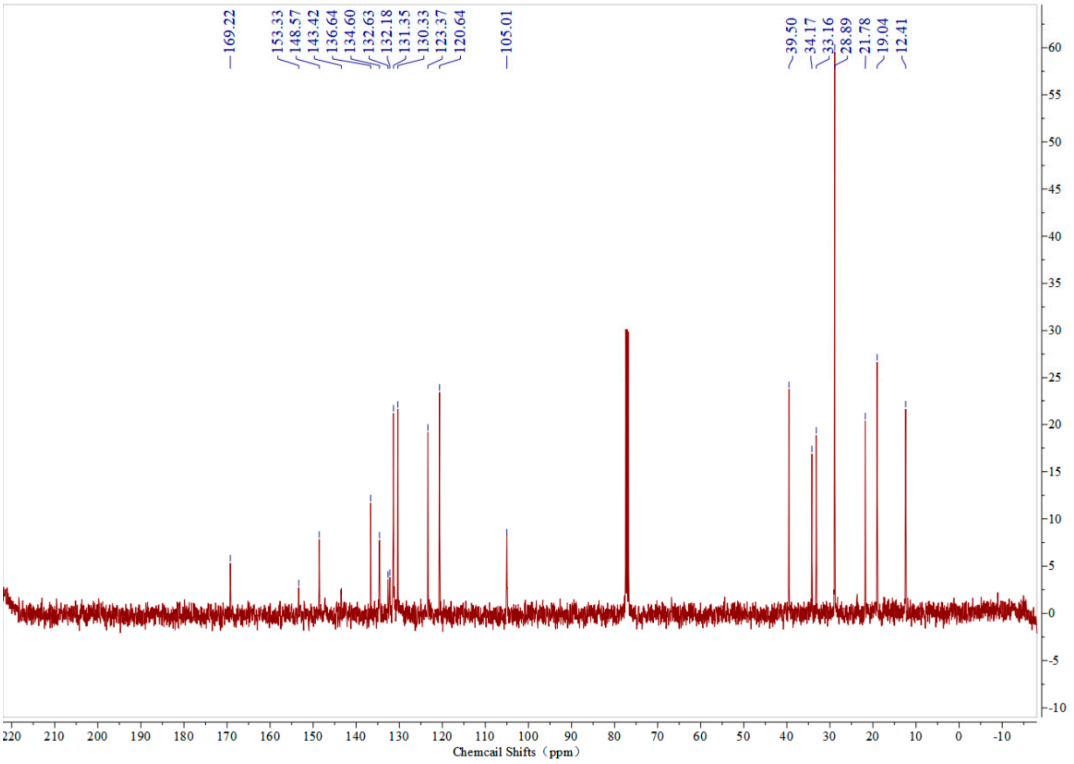

Figure S50. The ^{13}C NMR spectra of Compound 1l

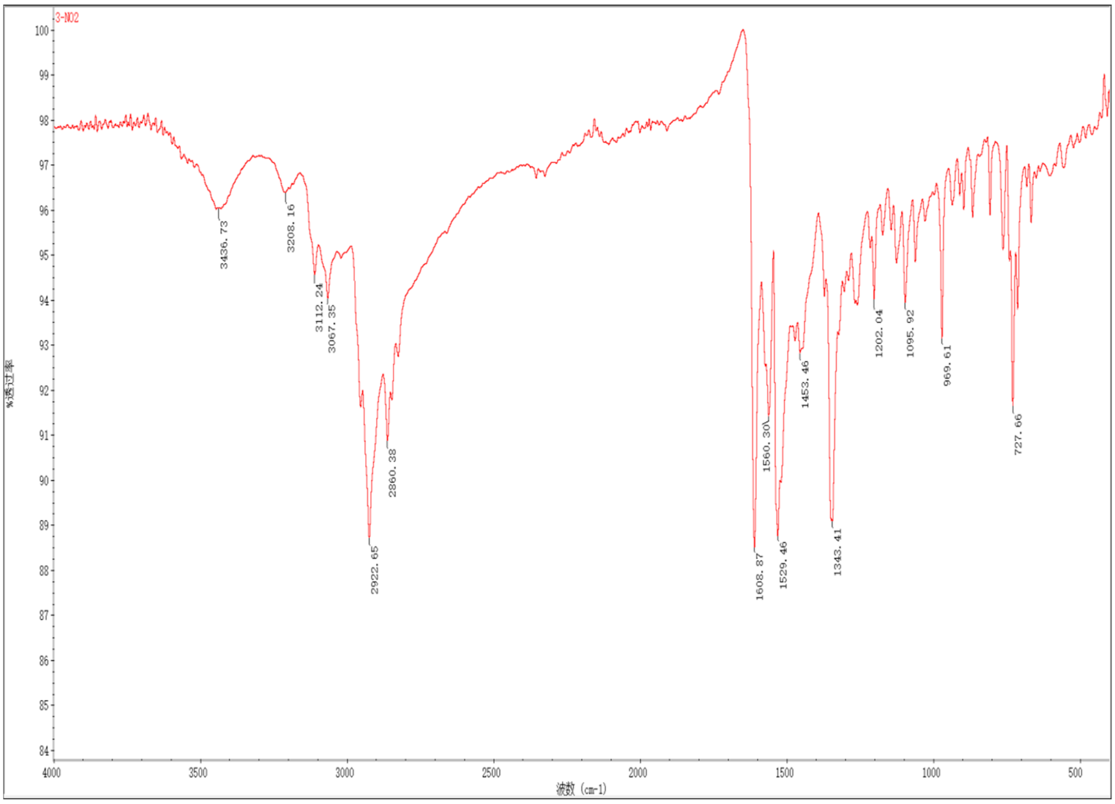

Figure S51. The FT-IR spectra of Compound 1l

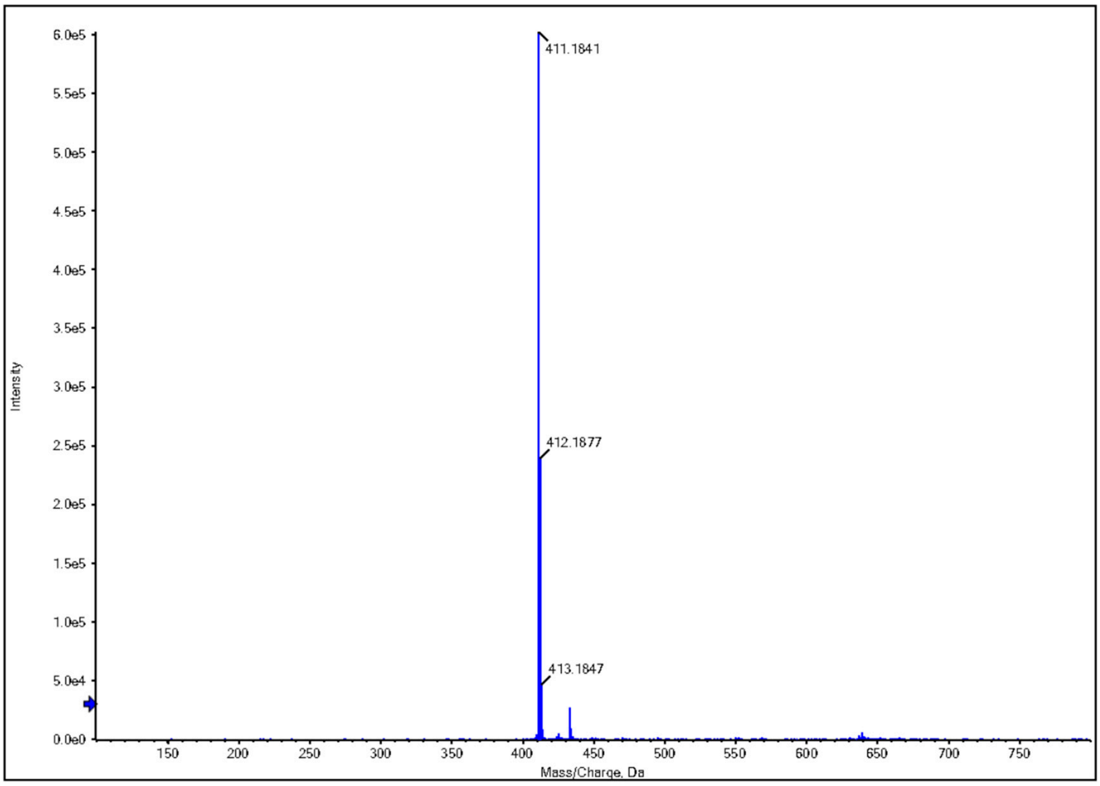

Figure S52. The HRMS spectra of Compound 1l

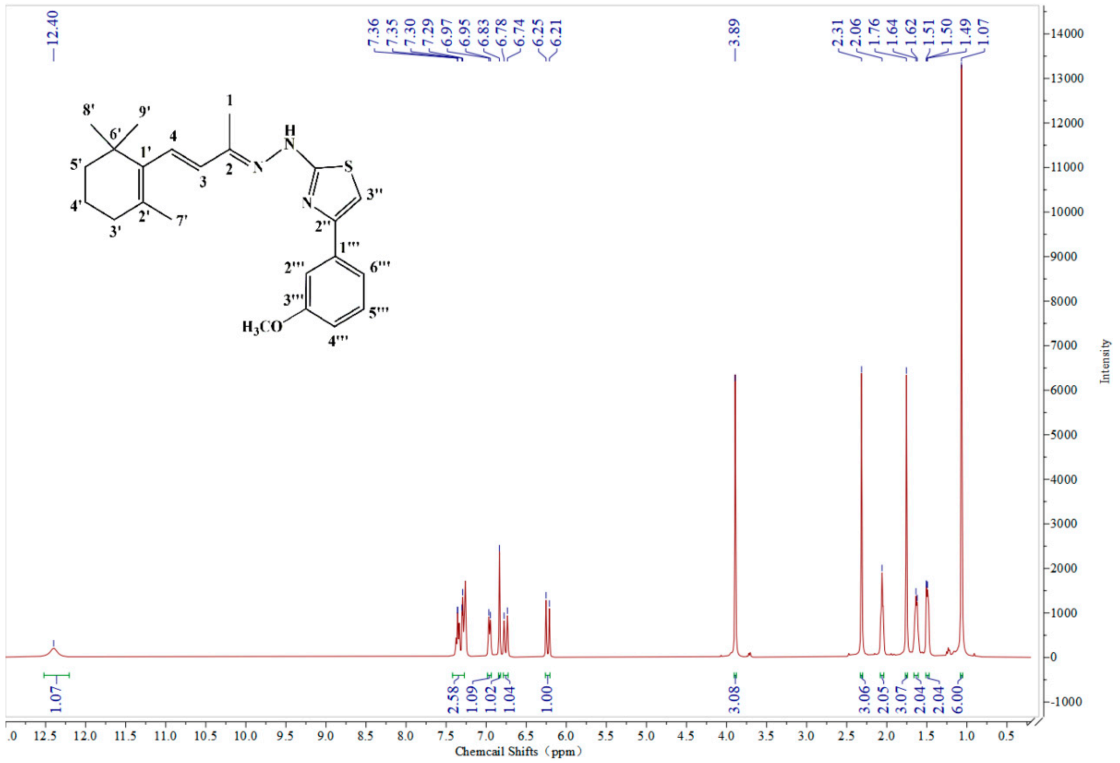

Figure S53. The ^{1}H NMR spectra Compound 1m

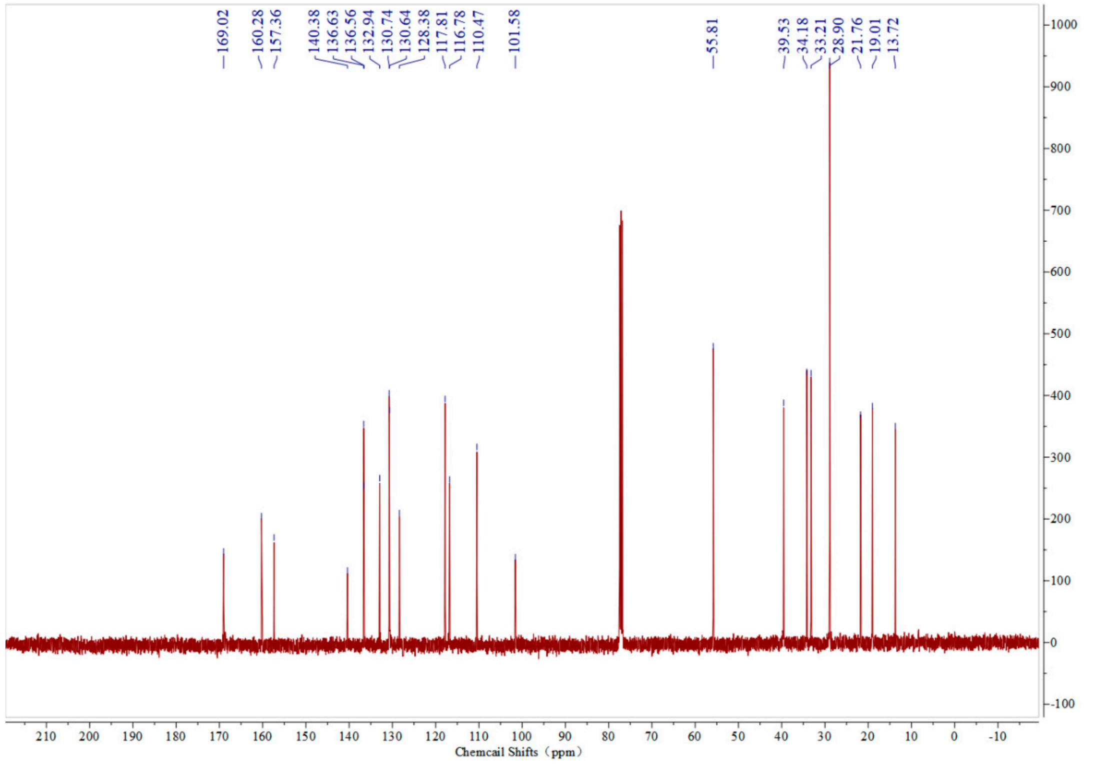

Figure S54. The ^{13}C NMR spectra of Compound 1m

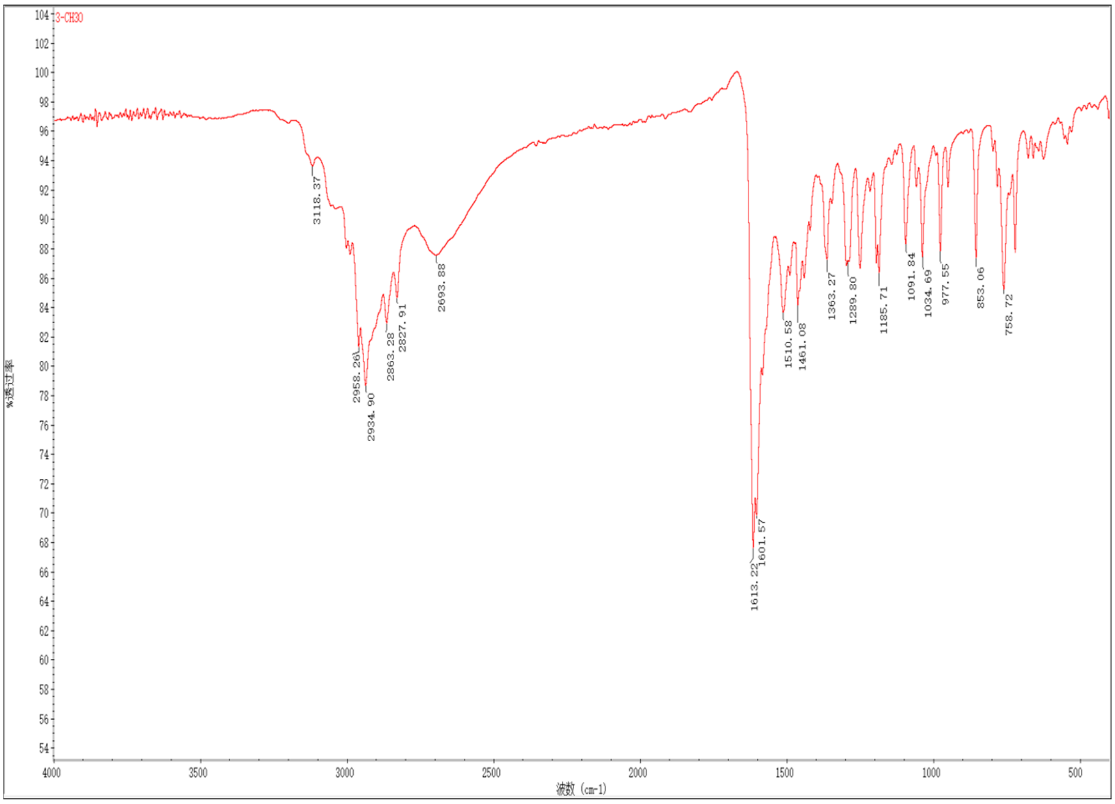

Figure S55. The FT-IR spectra of Compound 1m

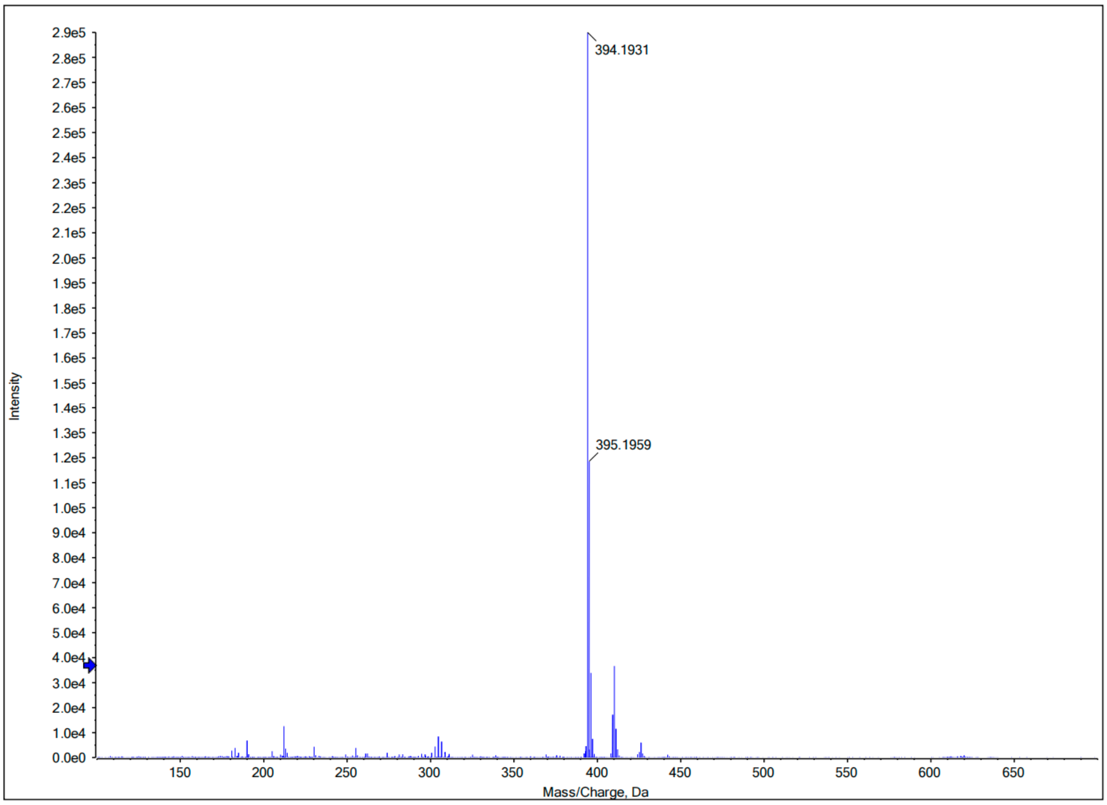

Figure S56. The HRMS spectra of Compound 1m

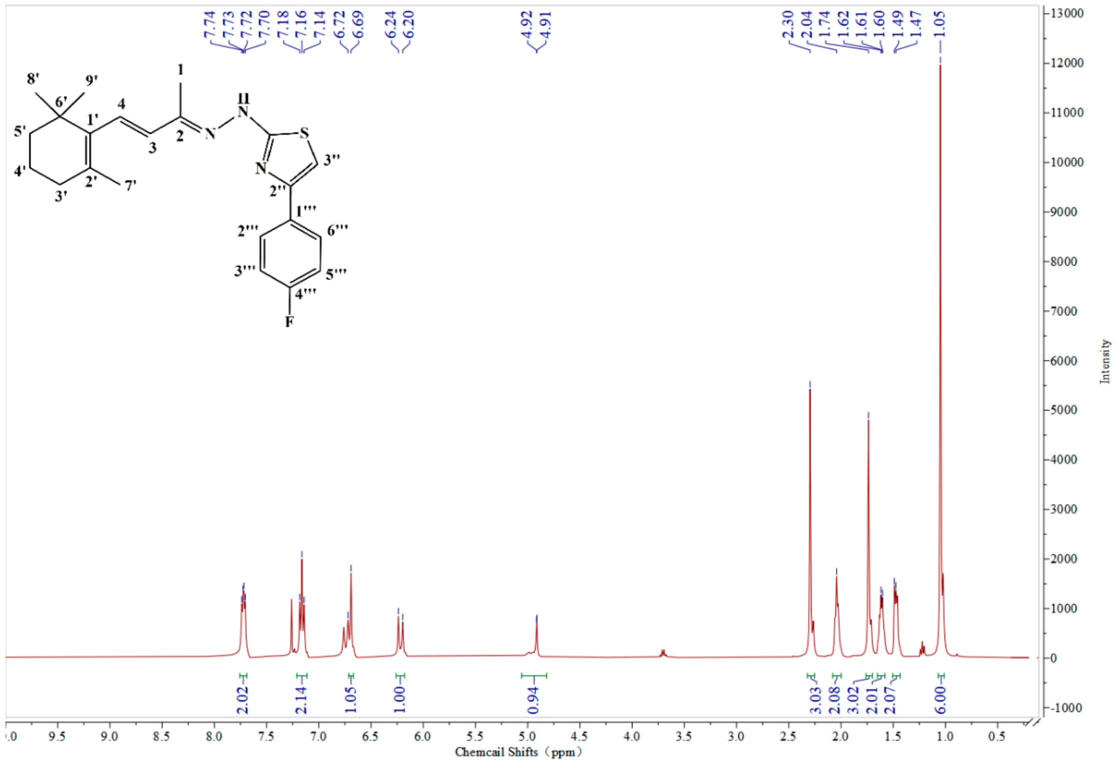

Figure S57. The ^{1}H NMR spectra Compound 1n

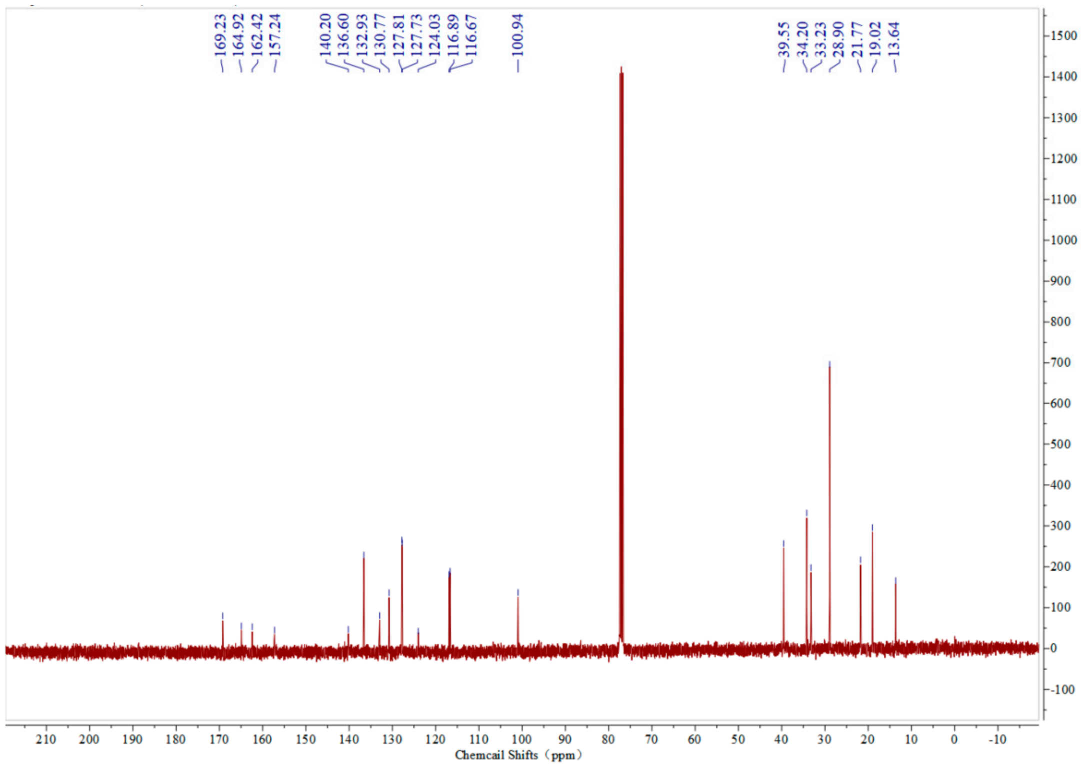

Figure S58. The ^{13}C NMR spectra of Compound 1n

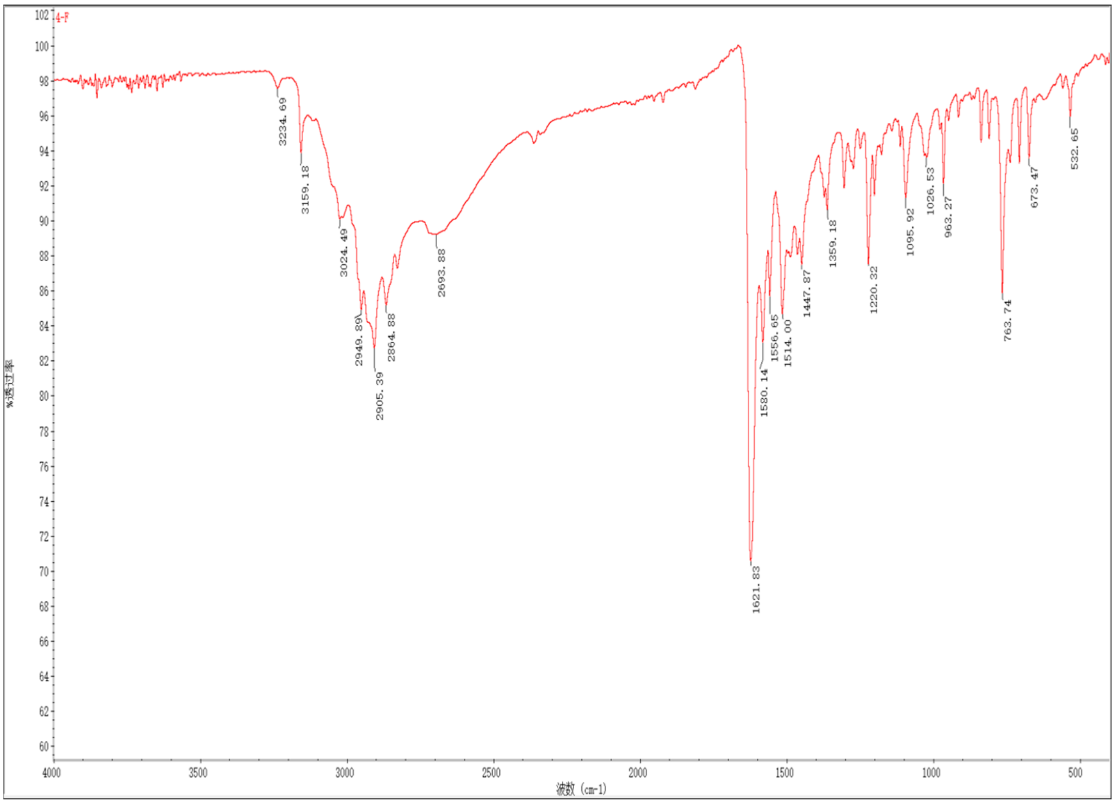

Figure S59. The FT-IR spectra of Compound 1n

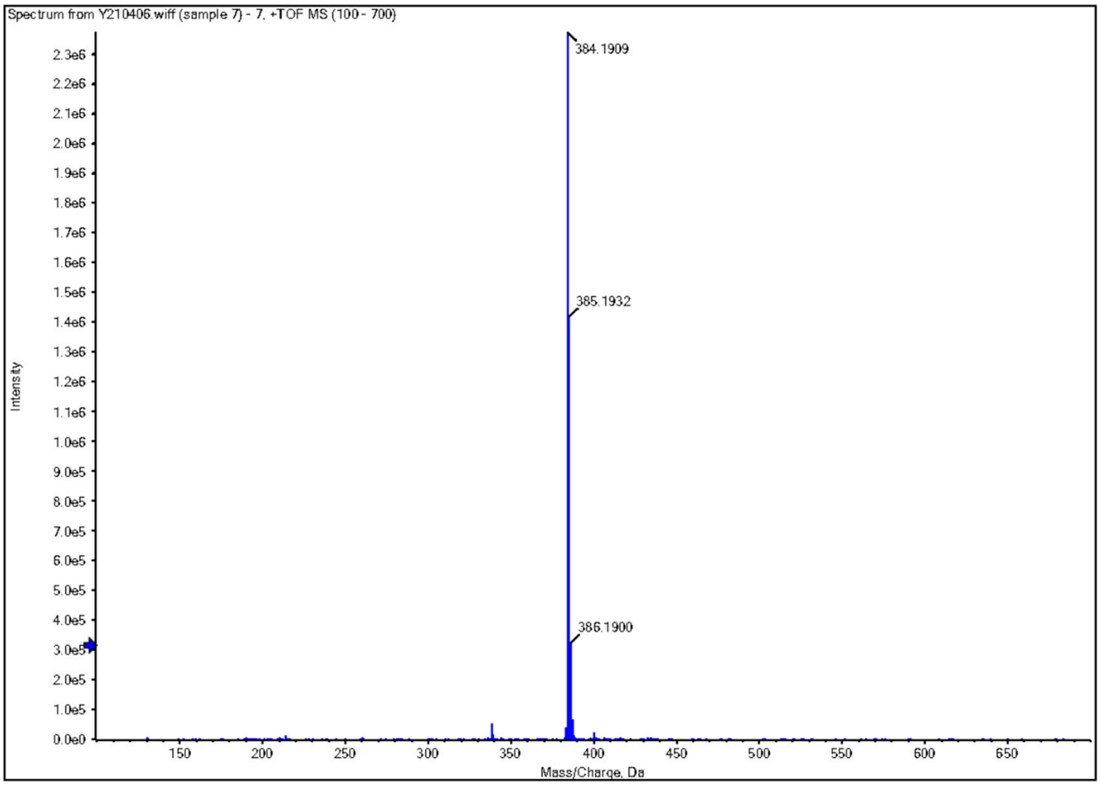

Figure S60. The HRMS spectra of Compound 1n

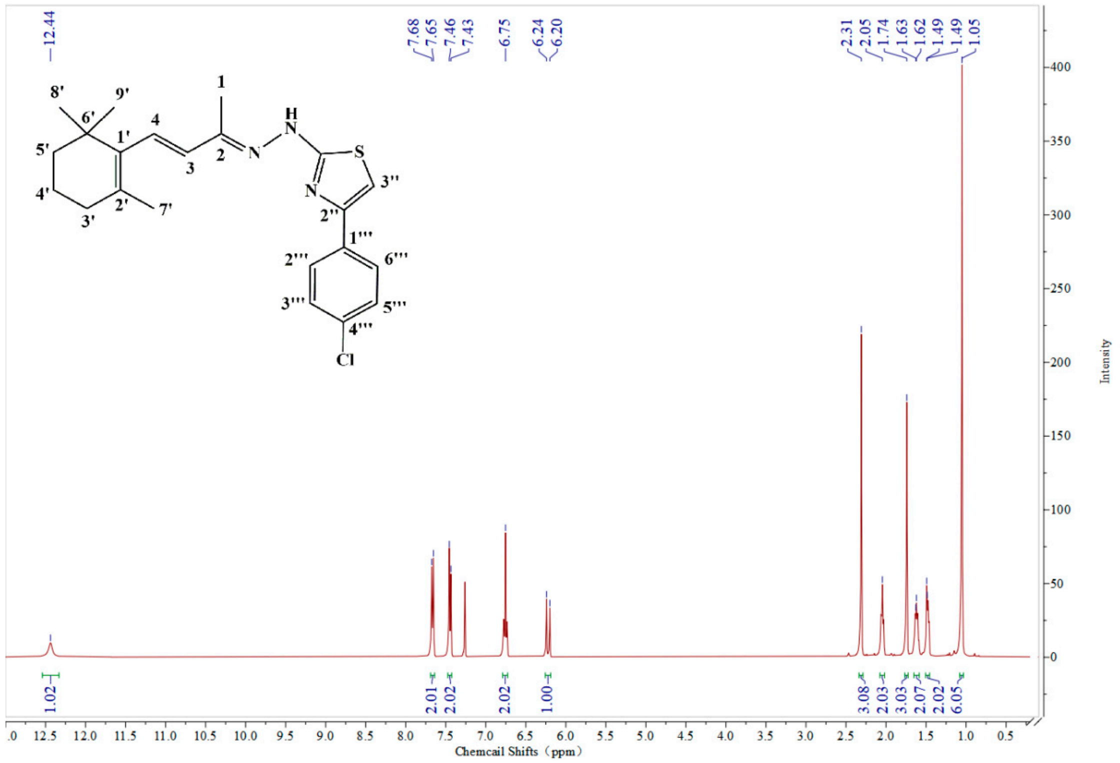

Figure S61. The ^{1}H NMR spectra Compound 1o

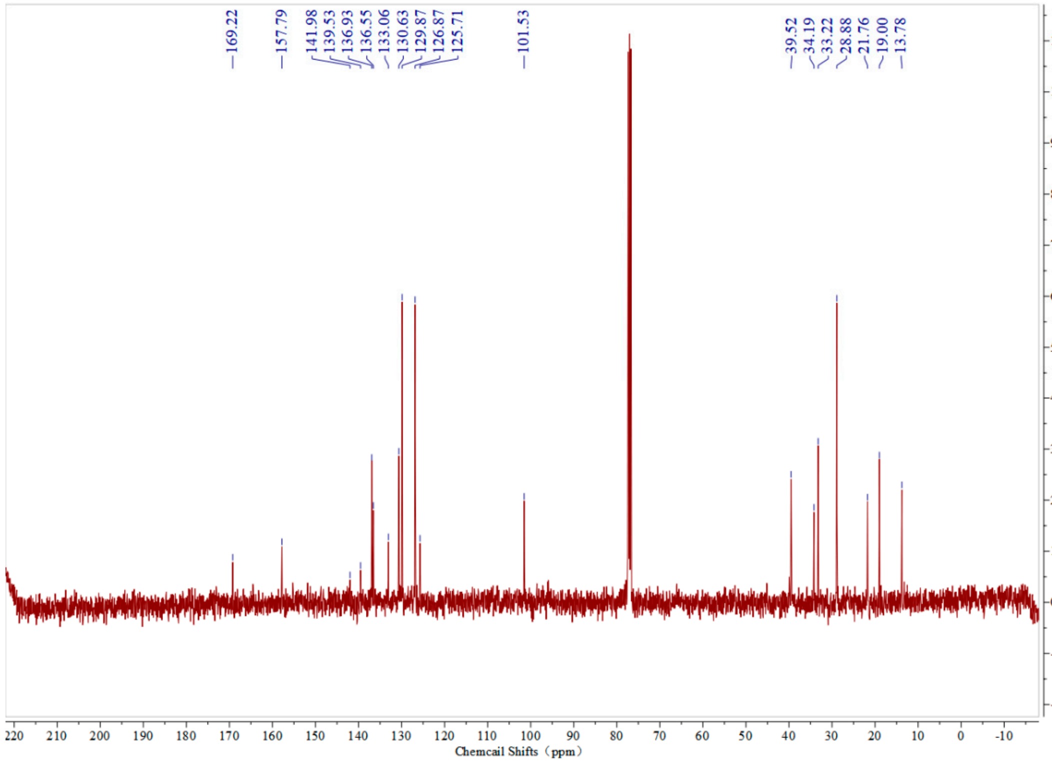

Figure S62. The ^{13}C NMR spectra of Compound 1o

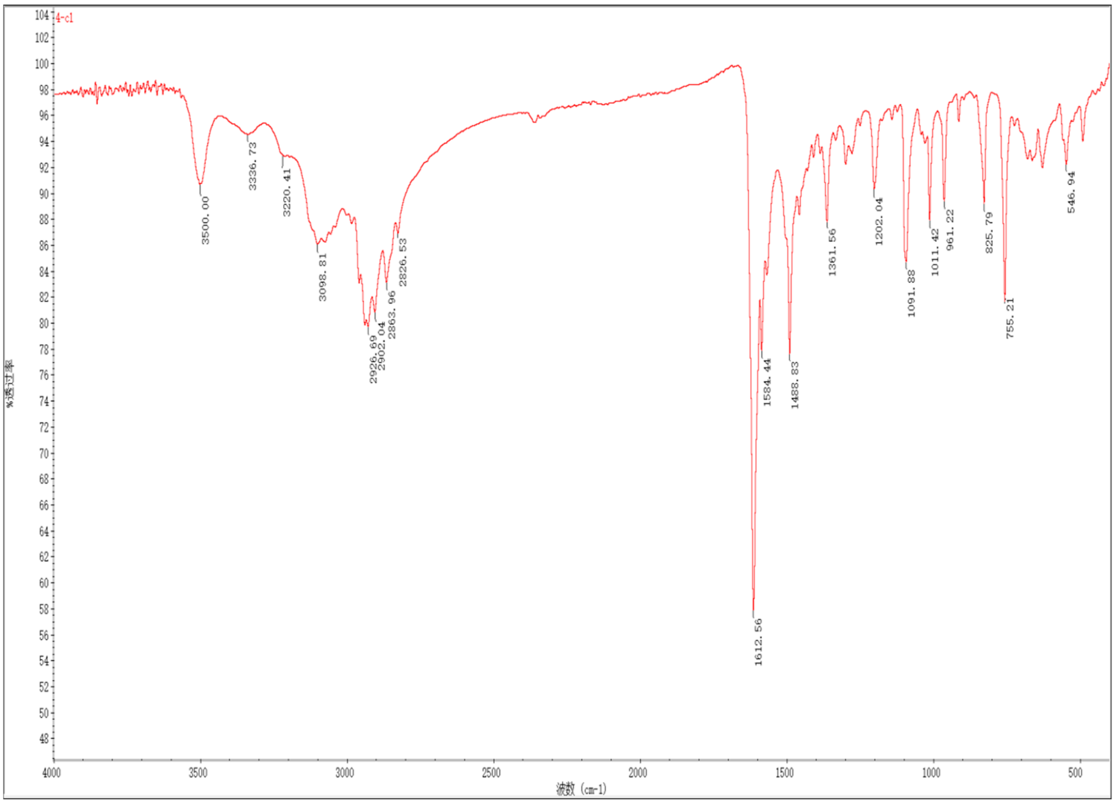

Figure S63. The FT-IR spectra of Compound 1o

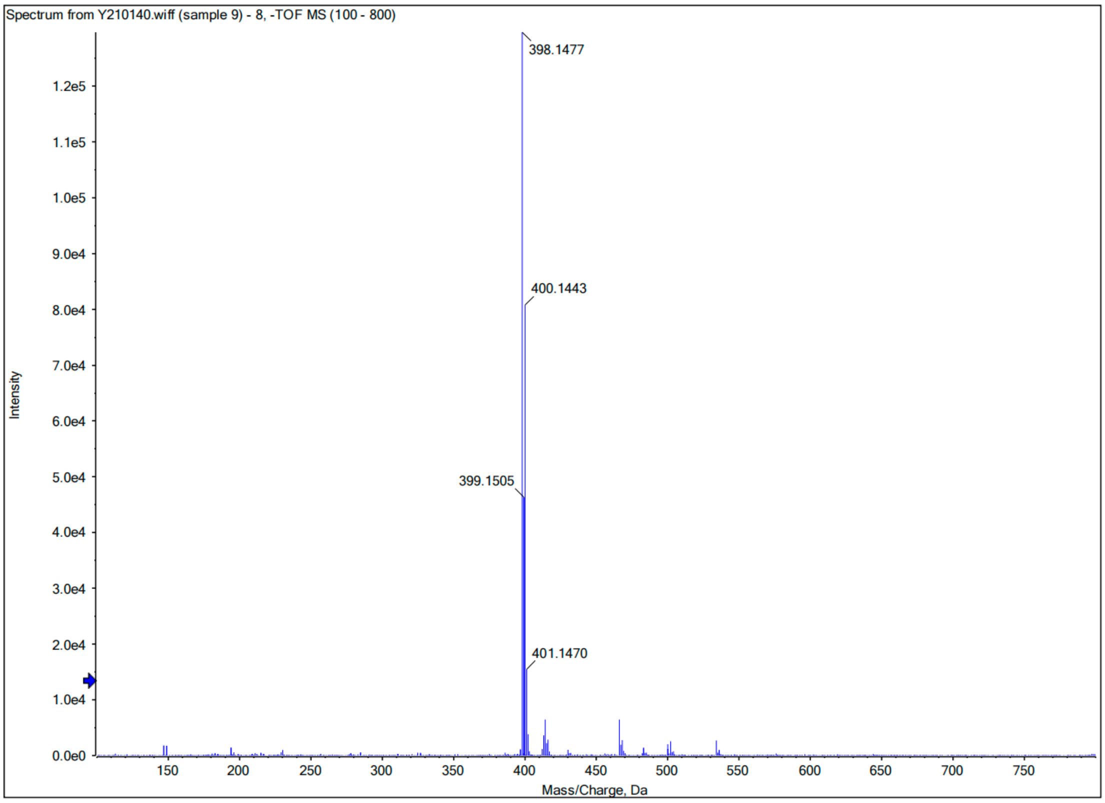

Figure S64. The HRMS spectra of Compound 1o

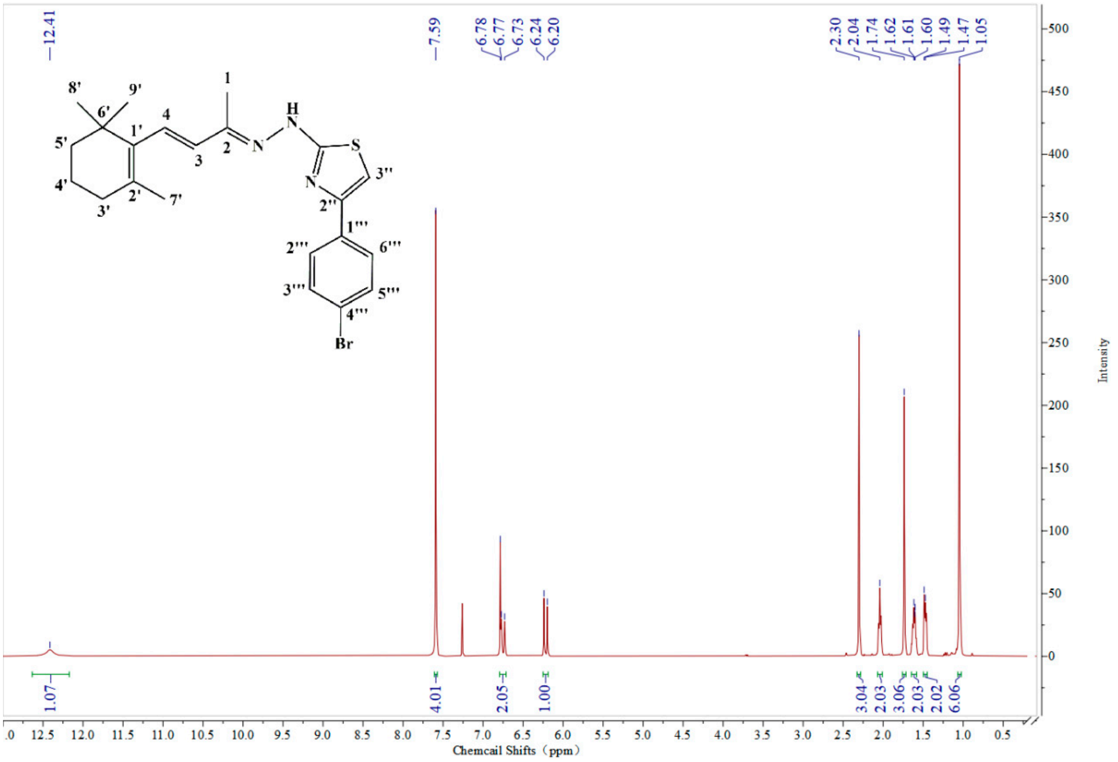

Figure S65. The ^{1}H NMR spectra Compound 1p

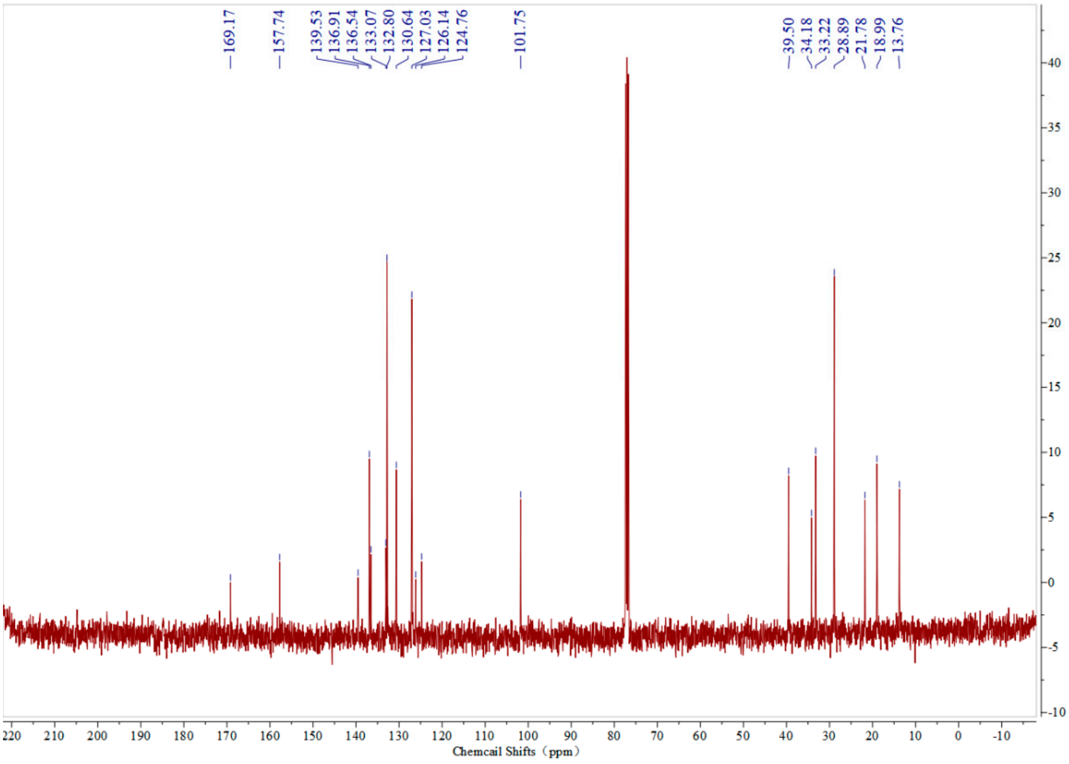

Figure S66. The ^{13}C NMR spectra of Compound 1p

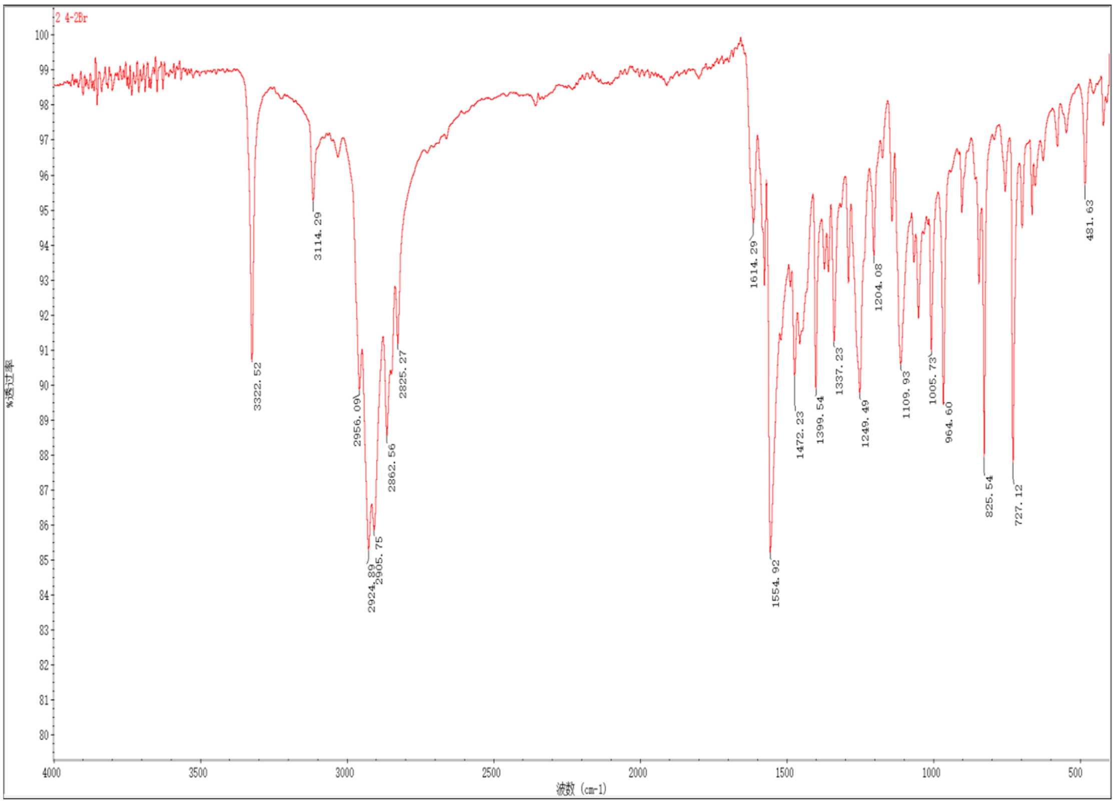

Figure S67. The FT-IR spectra of Compound 1p

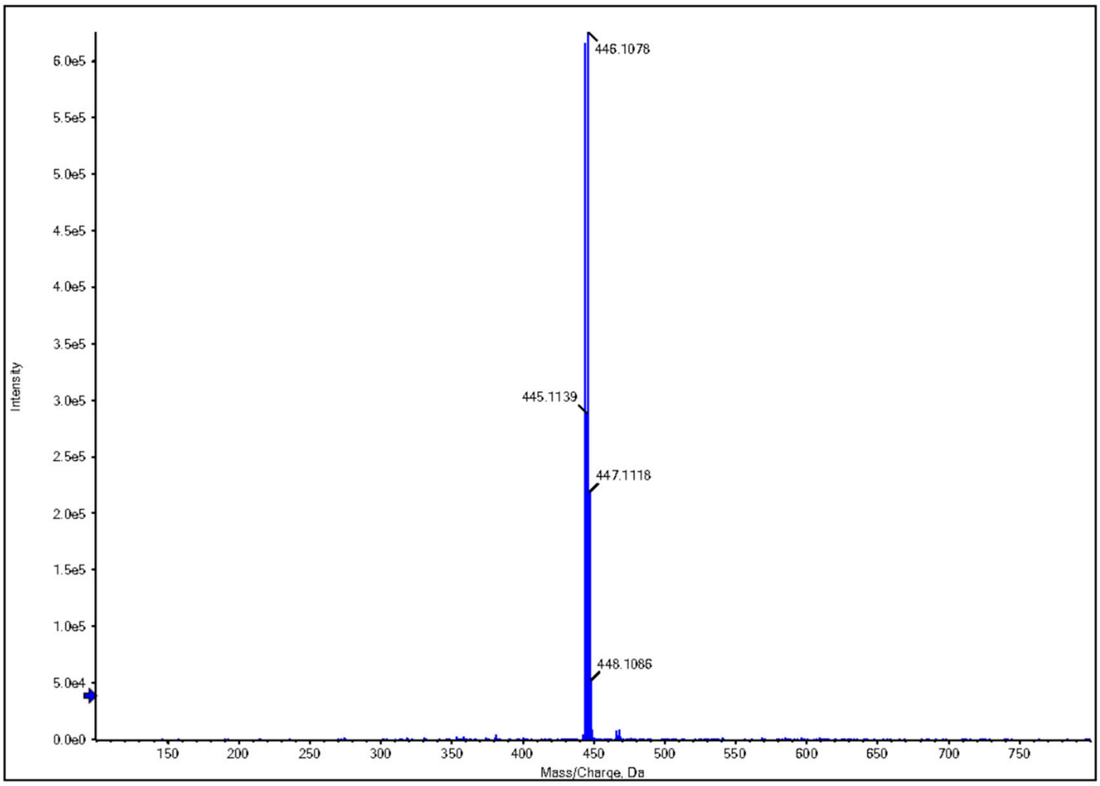

Figure S68. The HRMS spectra of Compound 1p

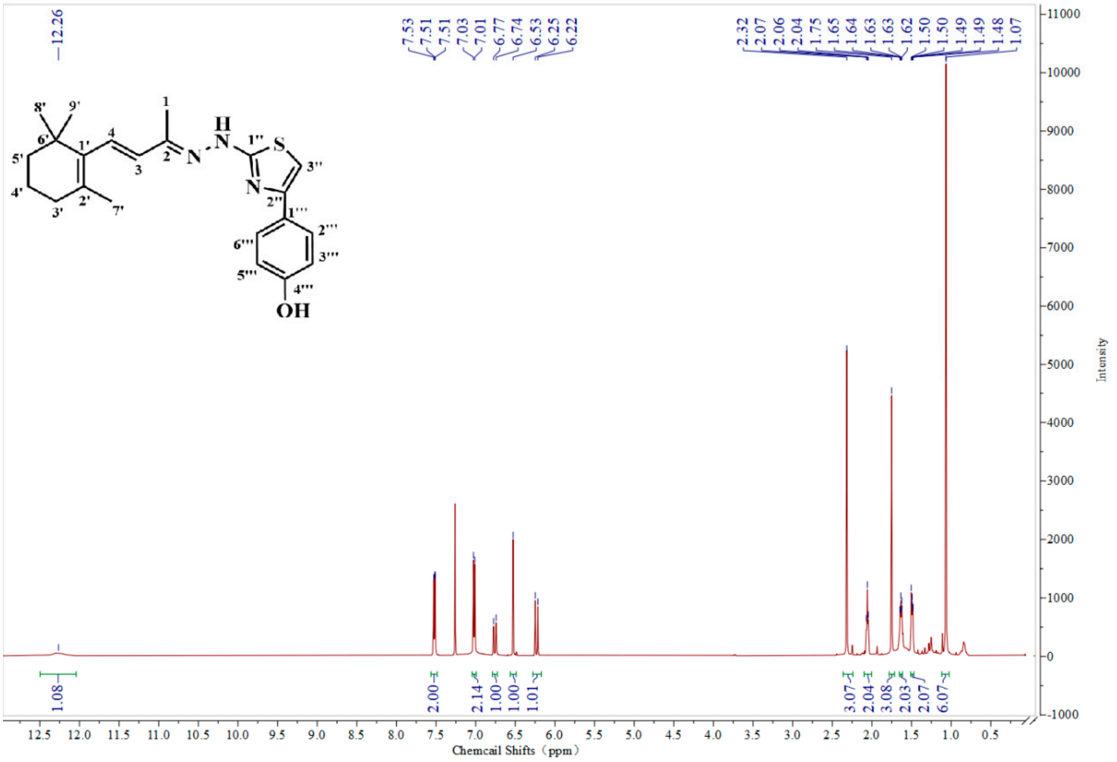

Figure S69. The ^{1}H NMR spectra Compound 1q

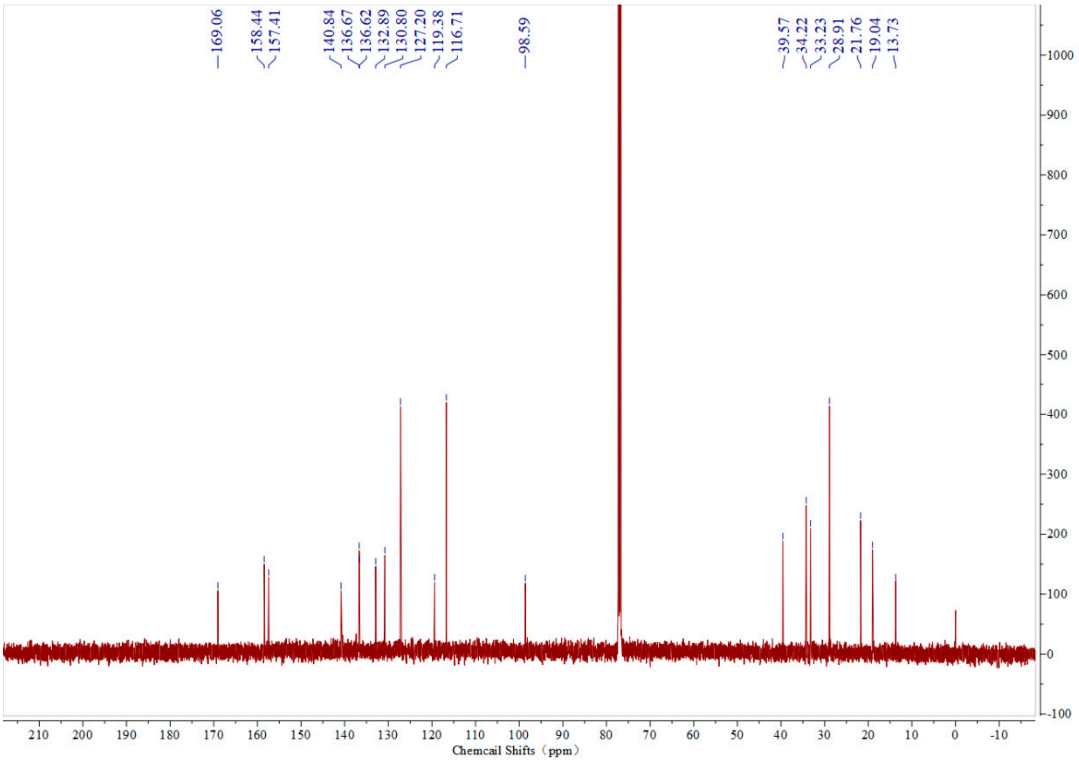

Figure S70. The ^{13}C NMR spectra of Compound 1q

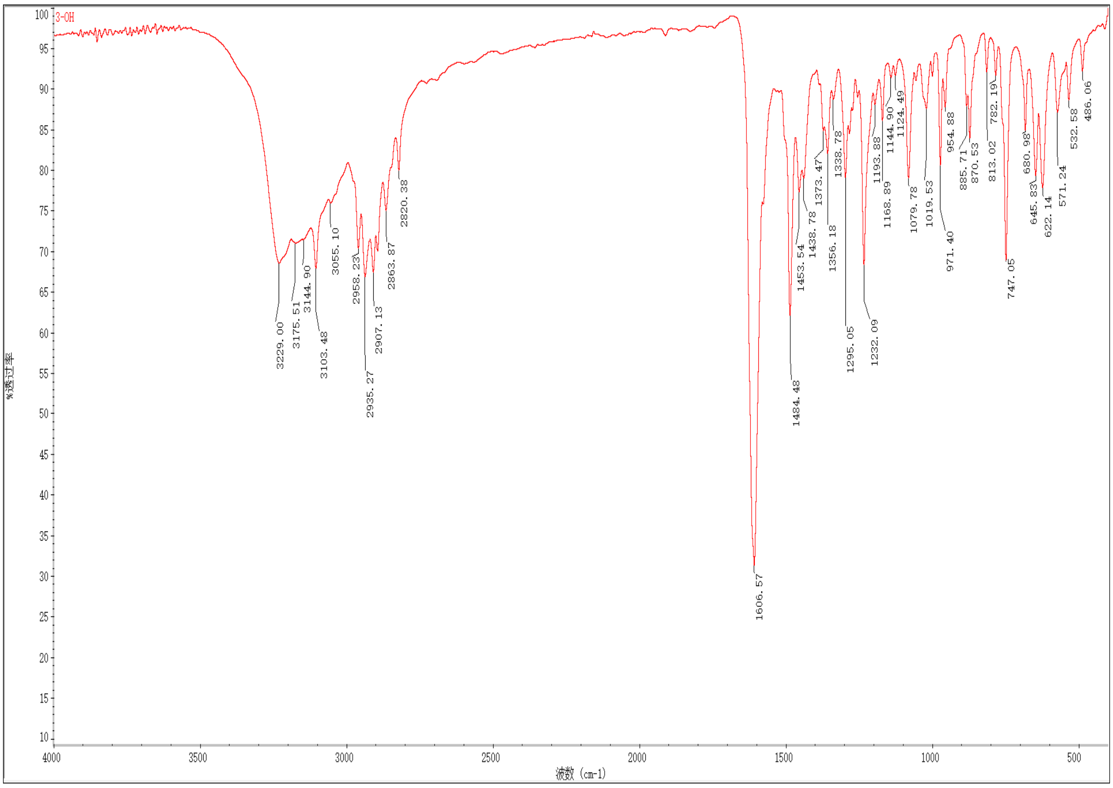

Figure S71. The FT-IR spectra of Compound 1q

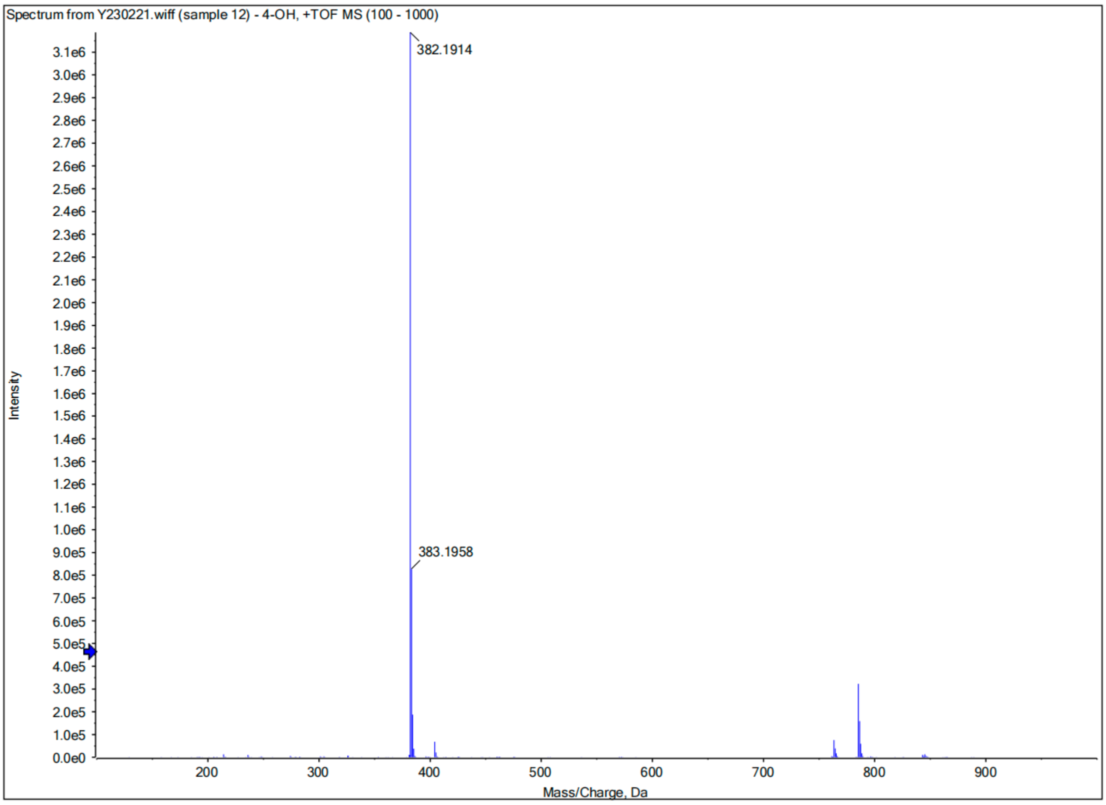

Figure S72. The HRMS spectra of Compound 1q

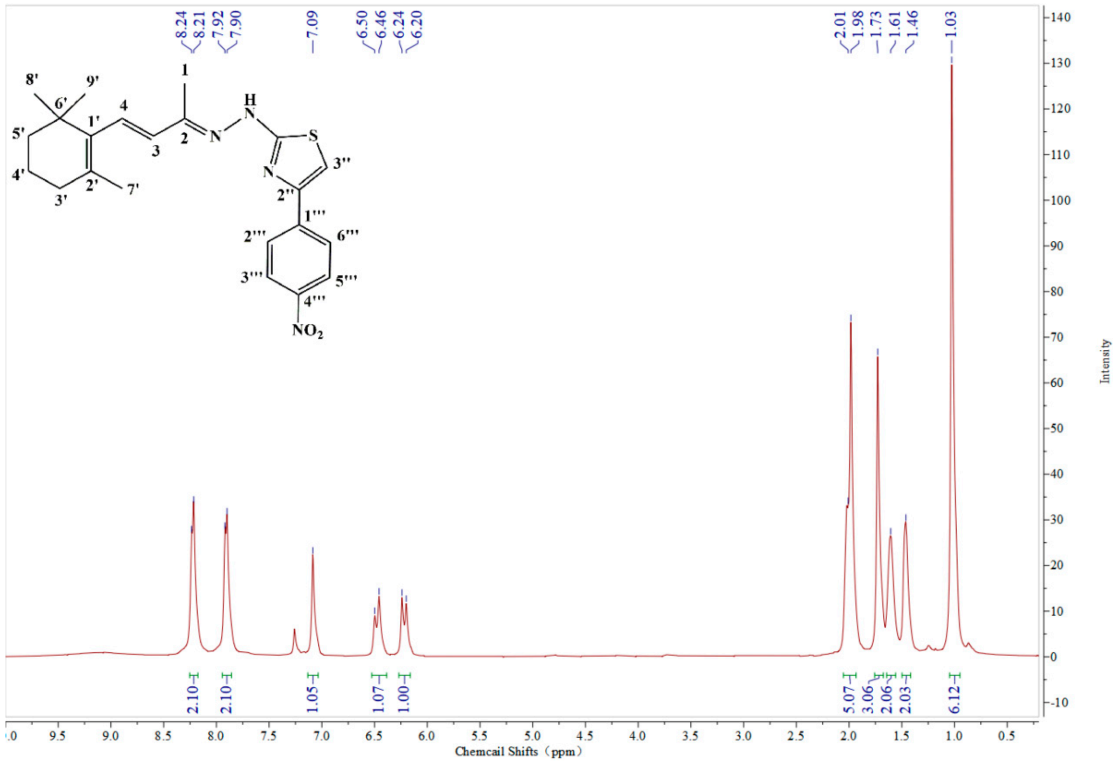

Figure S73. The ^{1}H NMR spectra Compound 1r

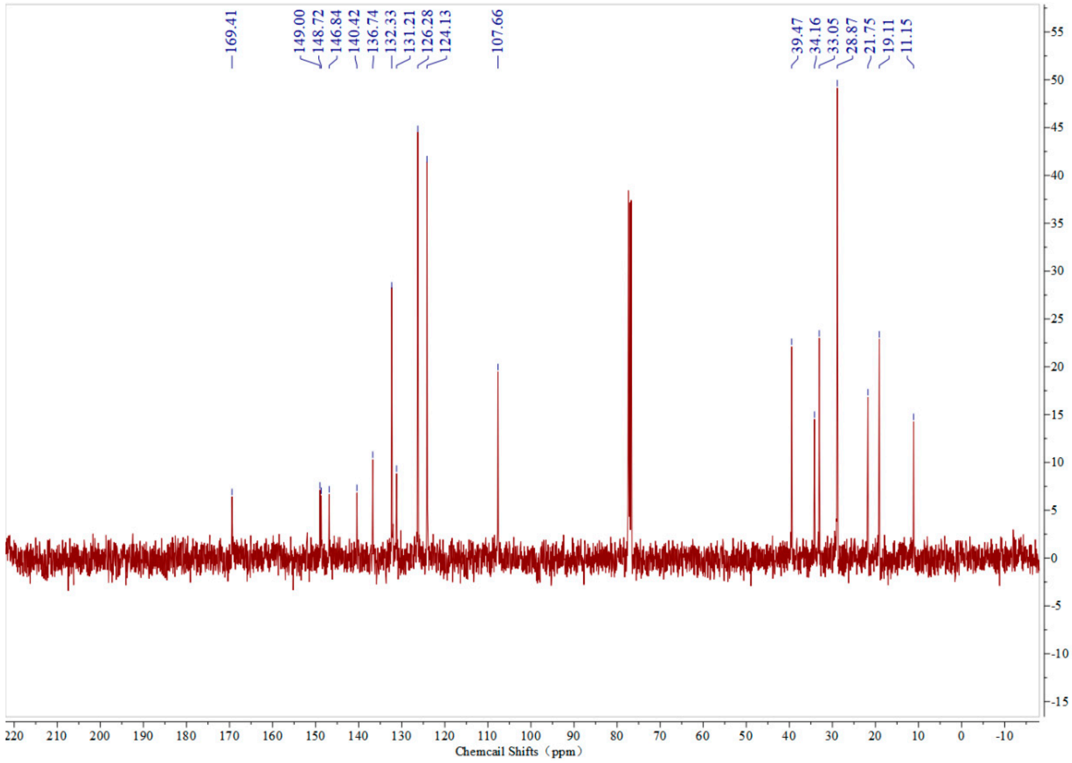

Figure S74. The ^{13}C NMR spectra of Compound 1r

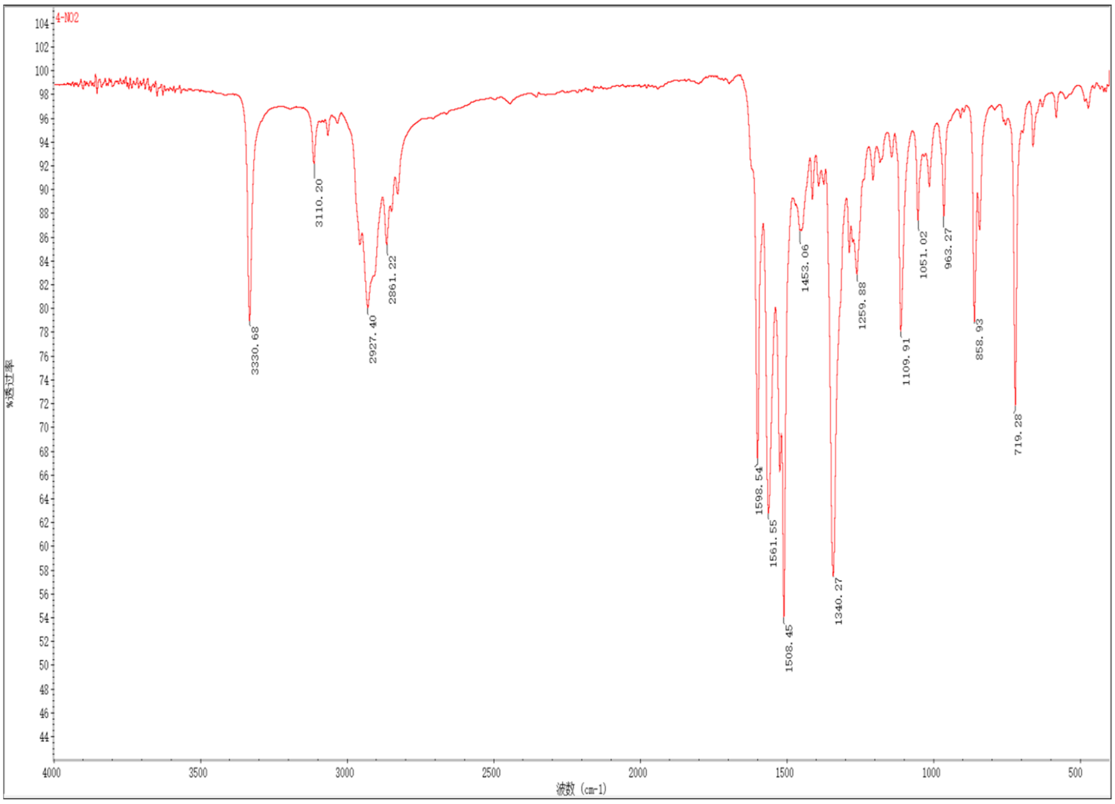

Figure S75. The FT-IR spectra of Compound 1r

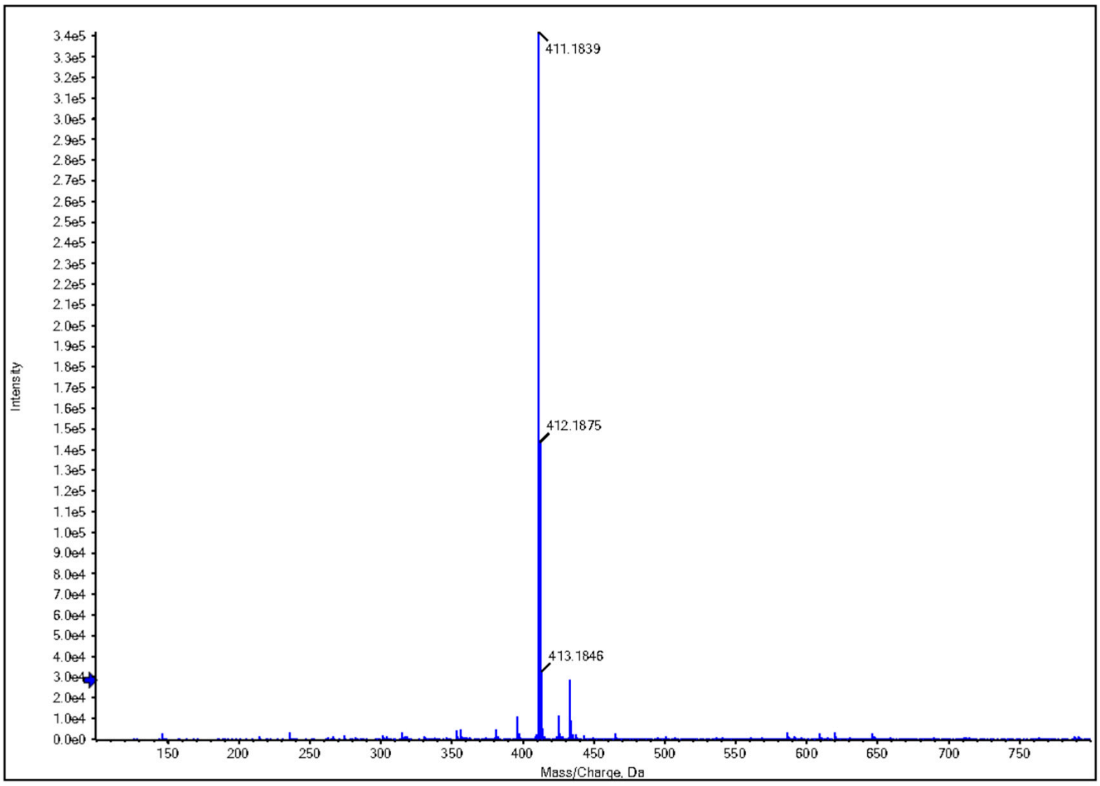

Figure S76. The HRMS spectra of Compound 1r

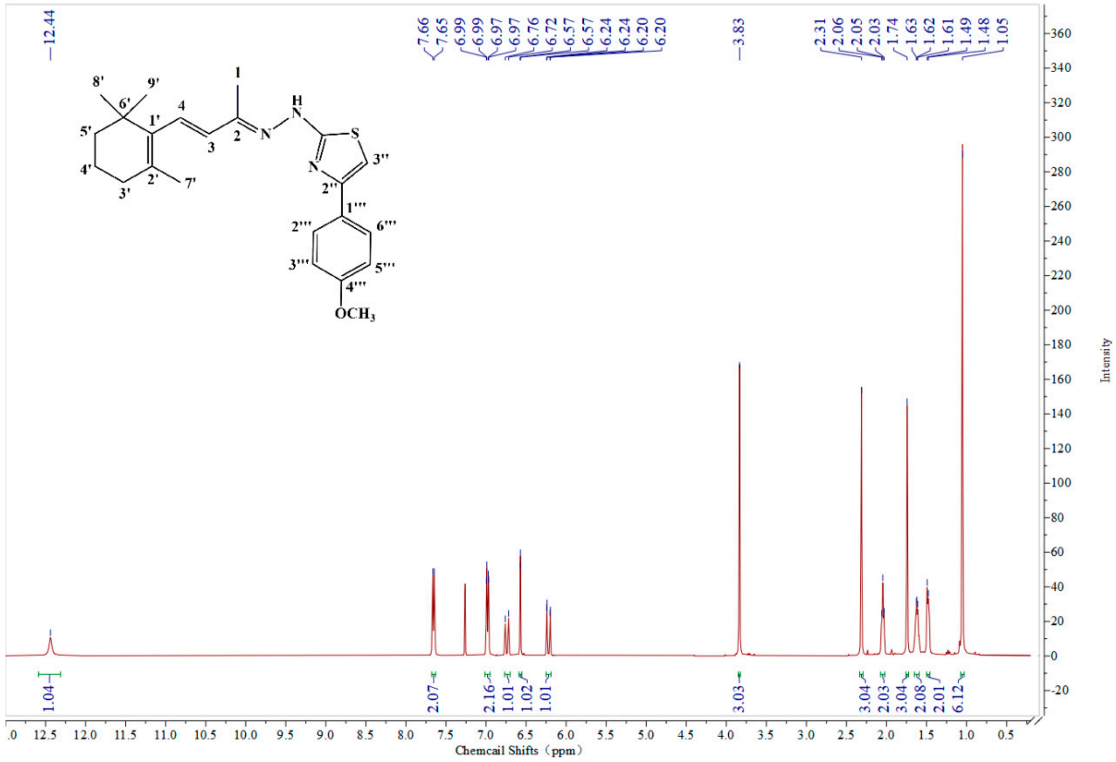

Figure S77. The ^{1}H NMR spectra Compound 1s

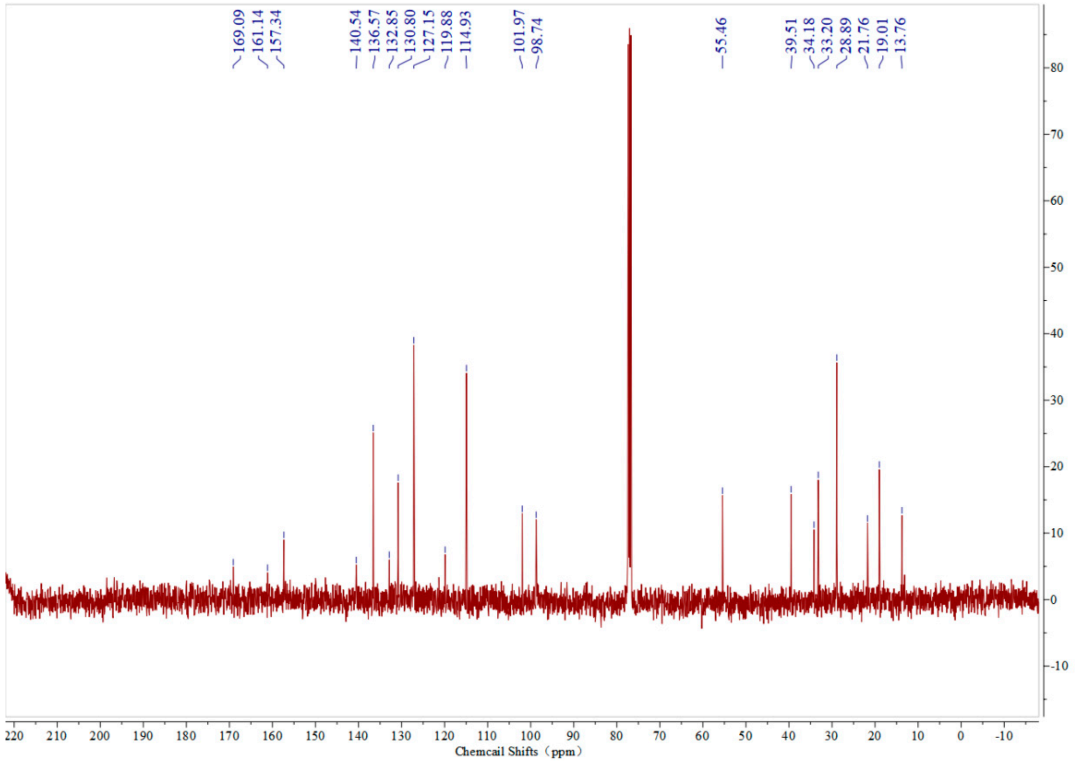

Figure S78. The ^{13}C NMR spectra of Compound 1s

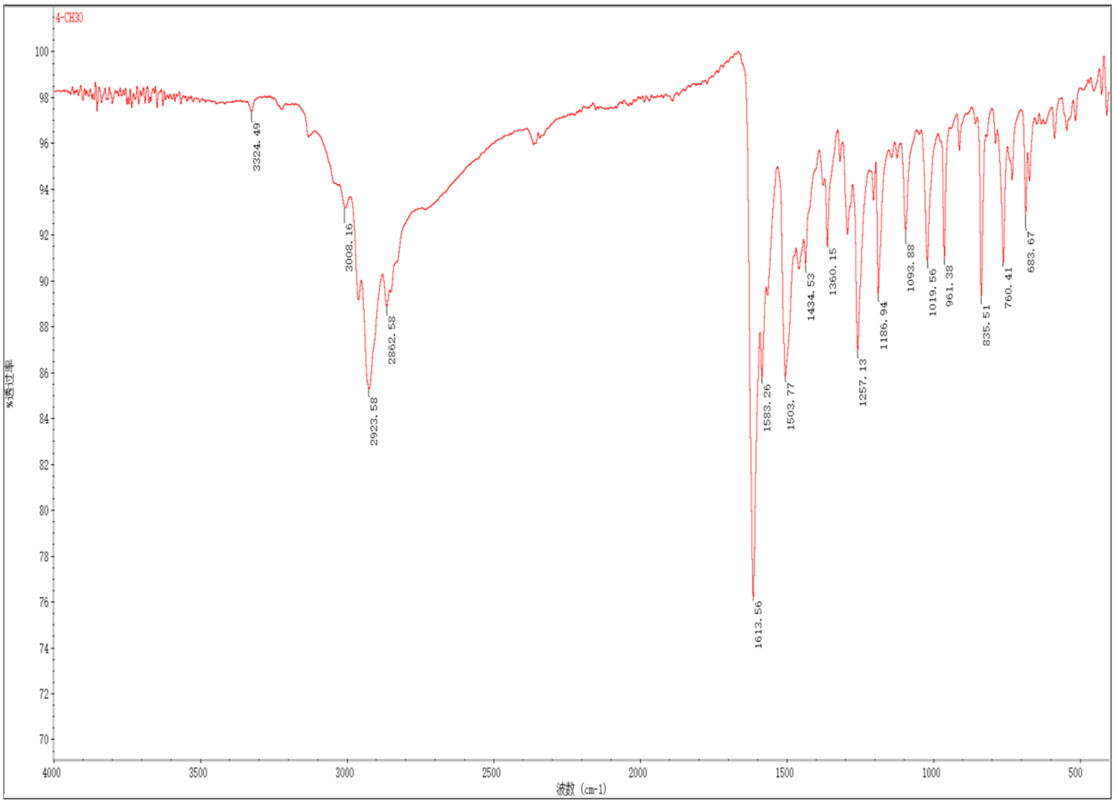

Figure S79. The FT-IR spectra of Compound 1s

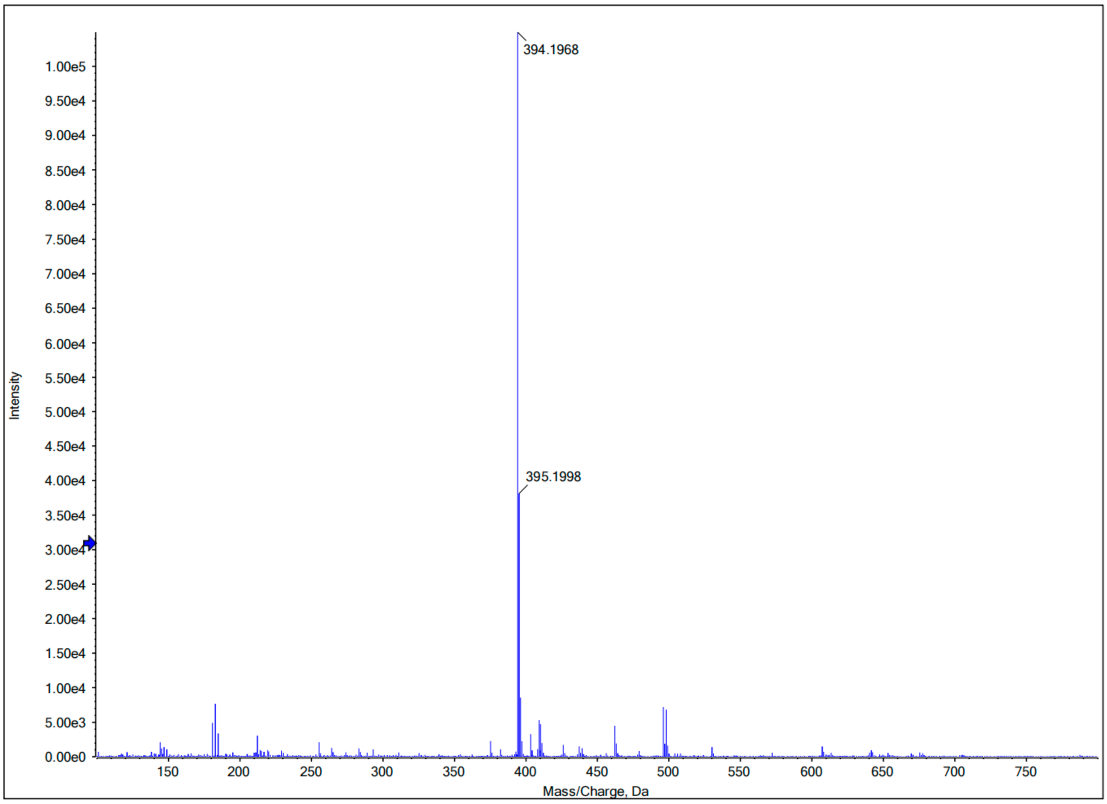

Figure S80. The HRMS spectra of Compound 1s

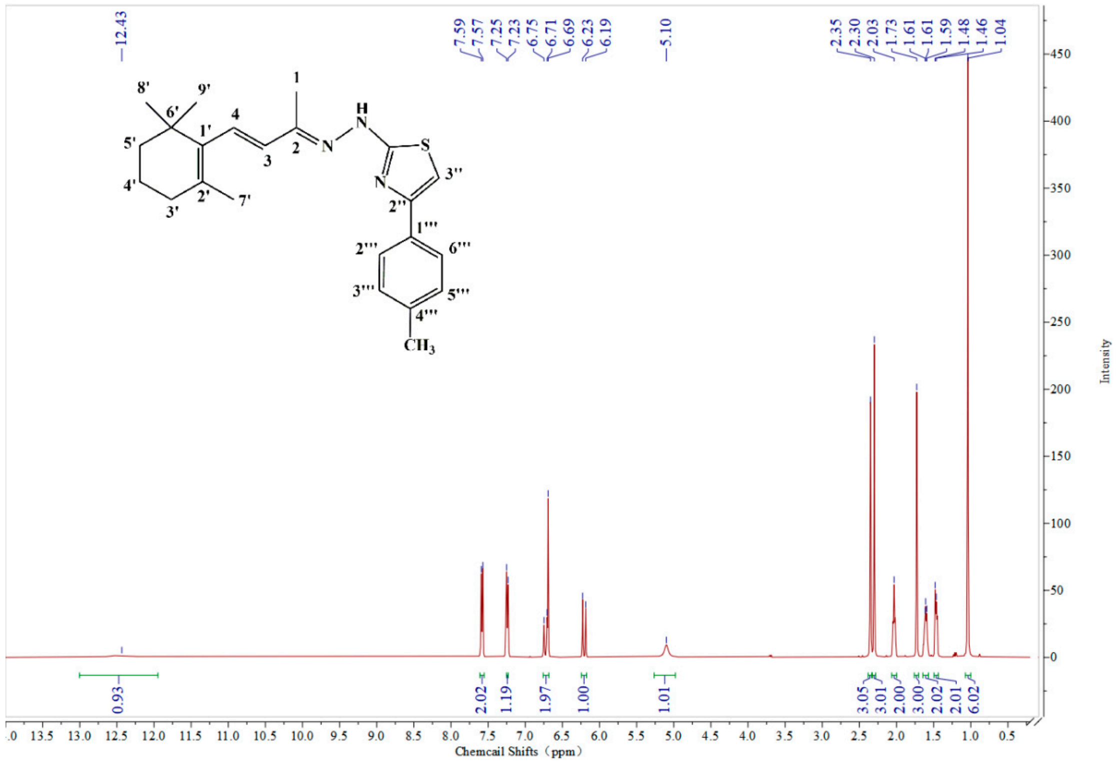

Figure S81. The ^{1}H NMR spectra Compound 1t

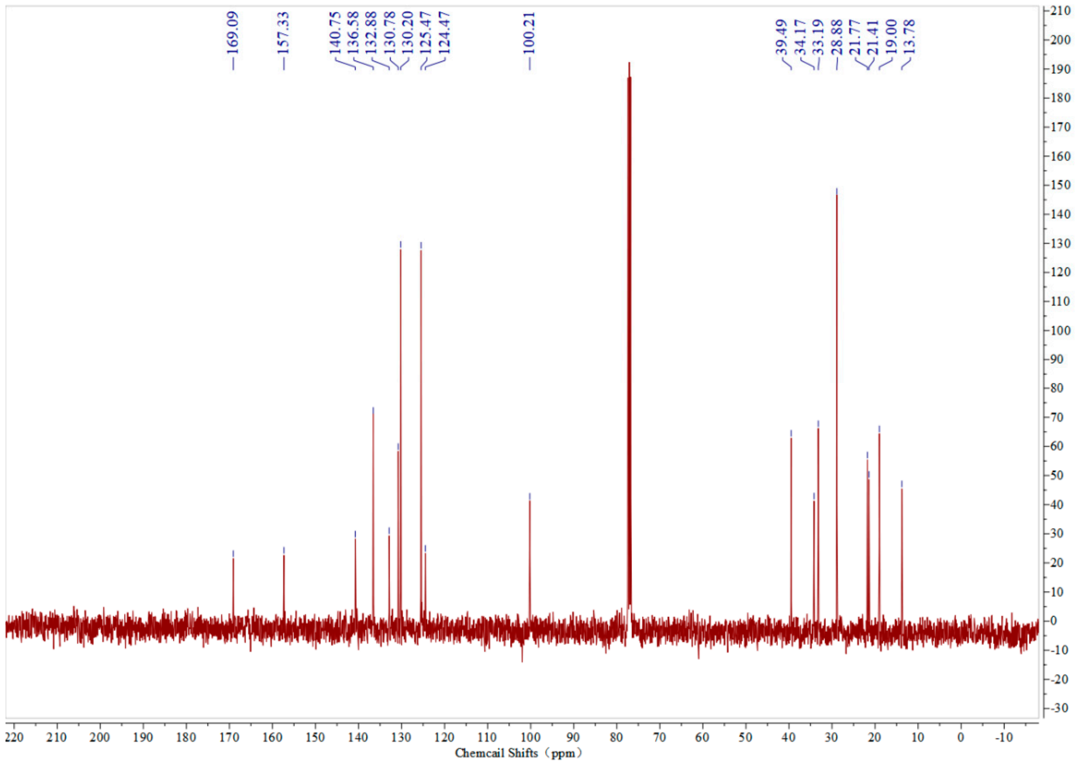

Figure S82. The ^{13}C NMR spectra of Compound 1t

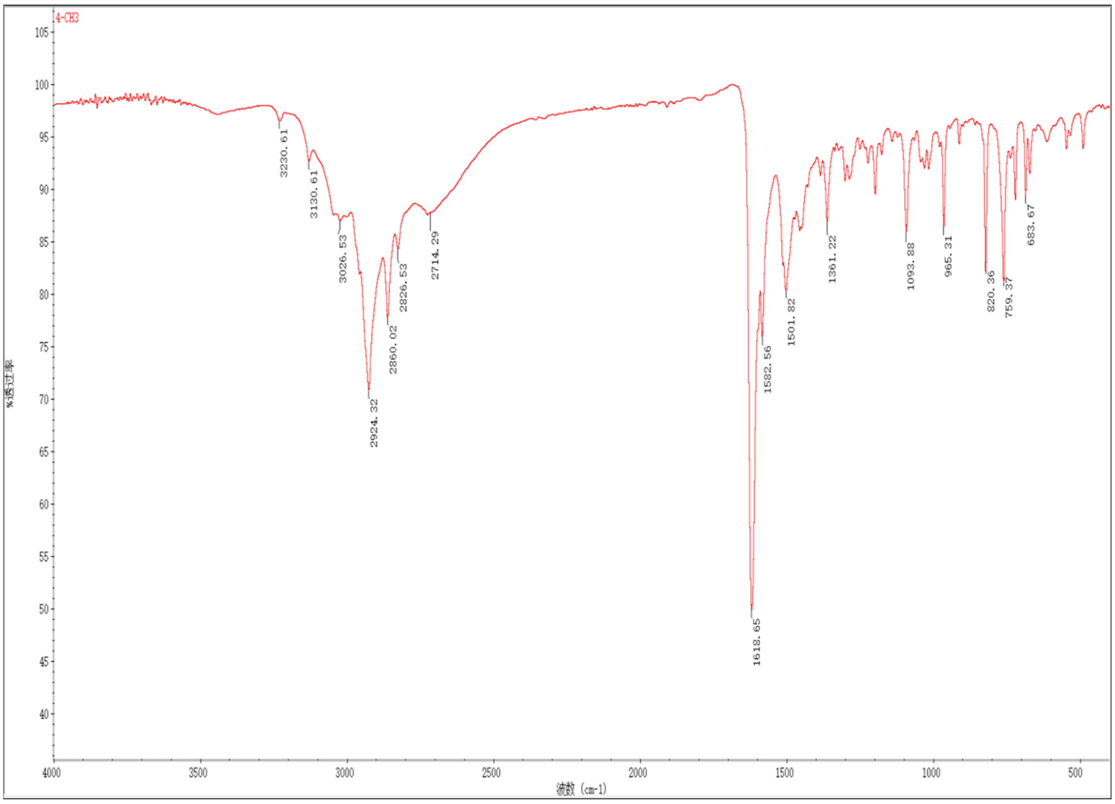

Figure S83. The FT-IR spectra of Compound 1t

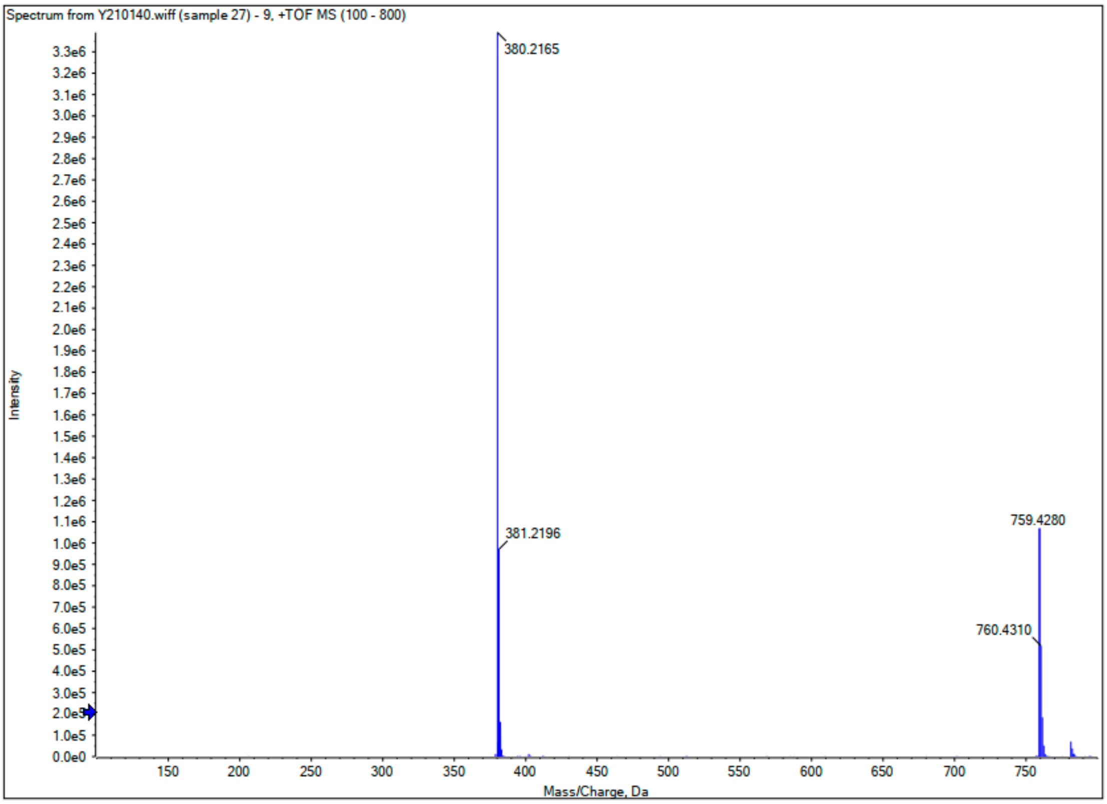

Figure S84. The HRMS spectra of Compound 1t

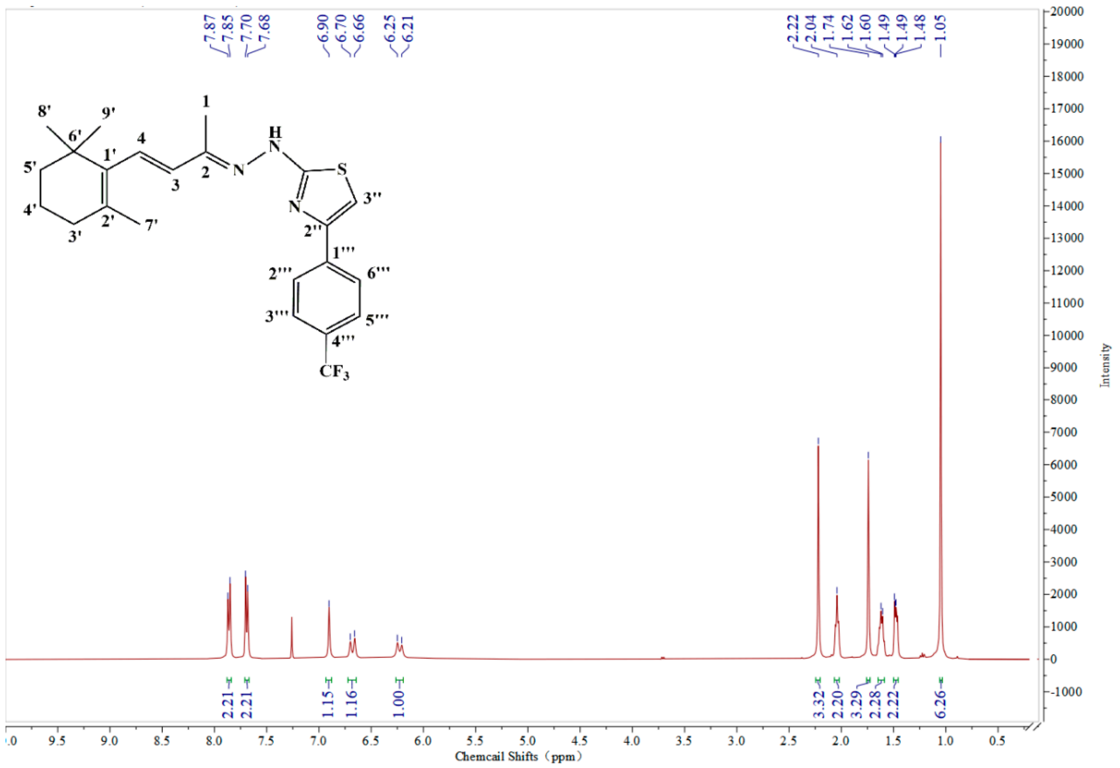

Figure S85. The ^{1}H NMR spectra Compound 1u

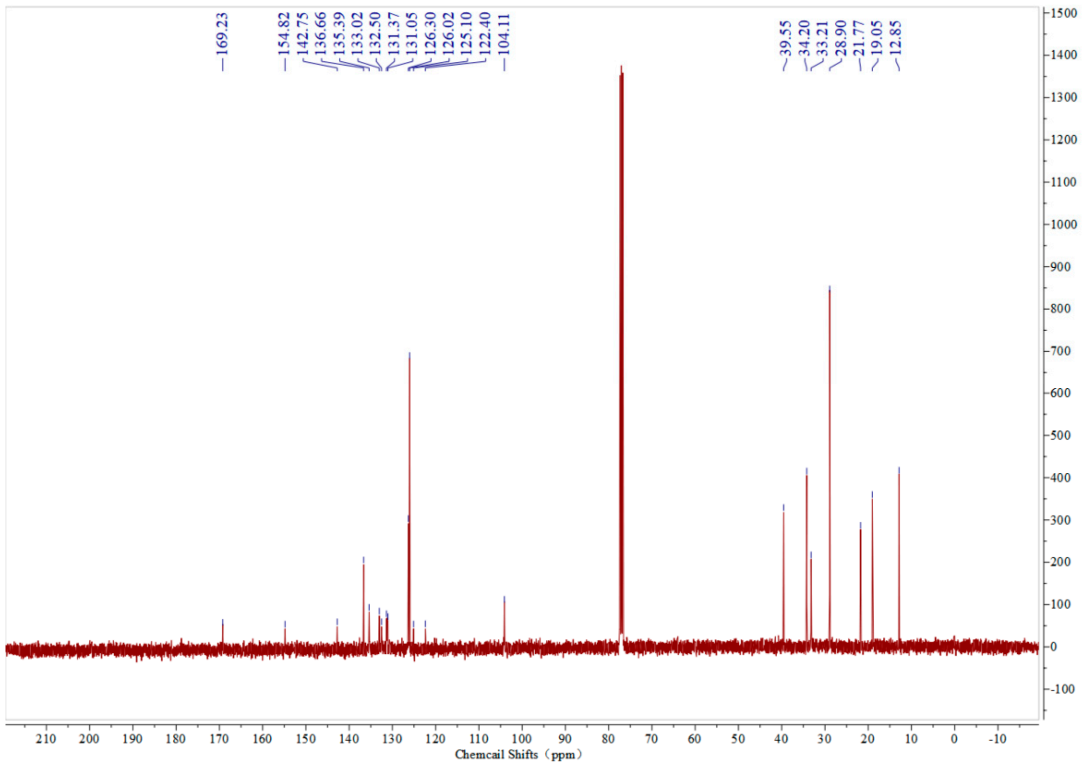

Figure S86. The ^{13}C NMR spectra of Compound 1u

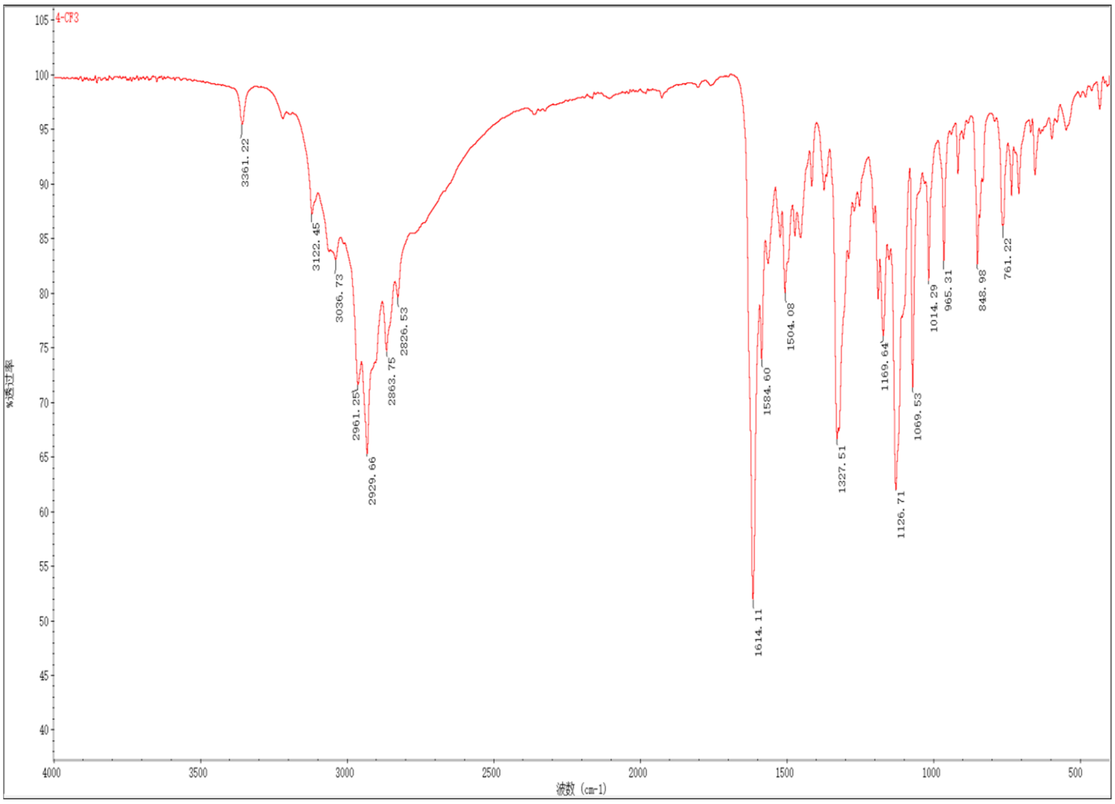

Figure S87. The FT-IR spectra of Compound 1u

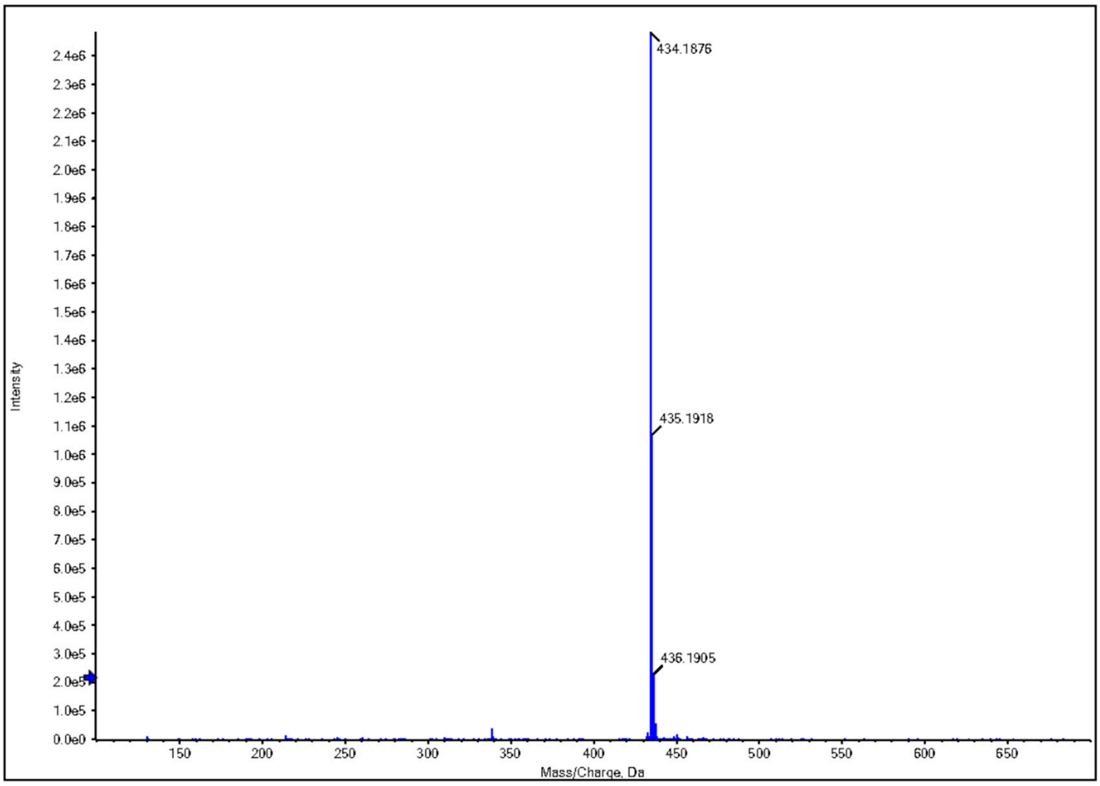

Figure S88. The HRMS spectra of Compound 1u

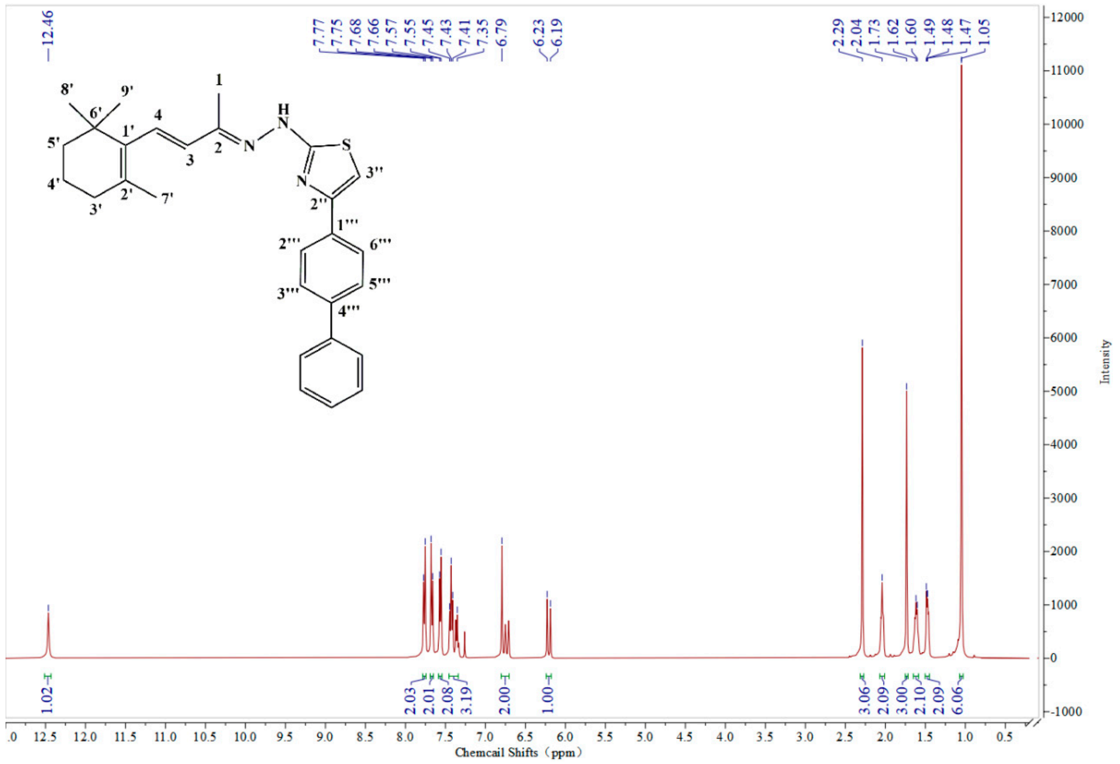

Figure S89. The ^{1}H NMR spectra Compound 1v

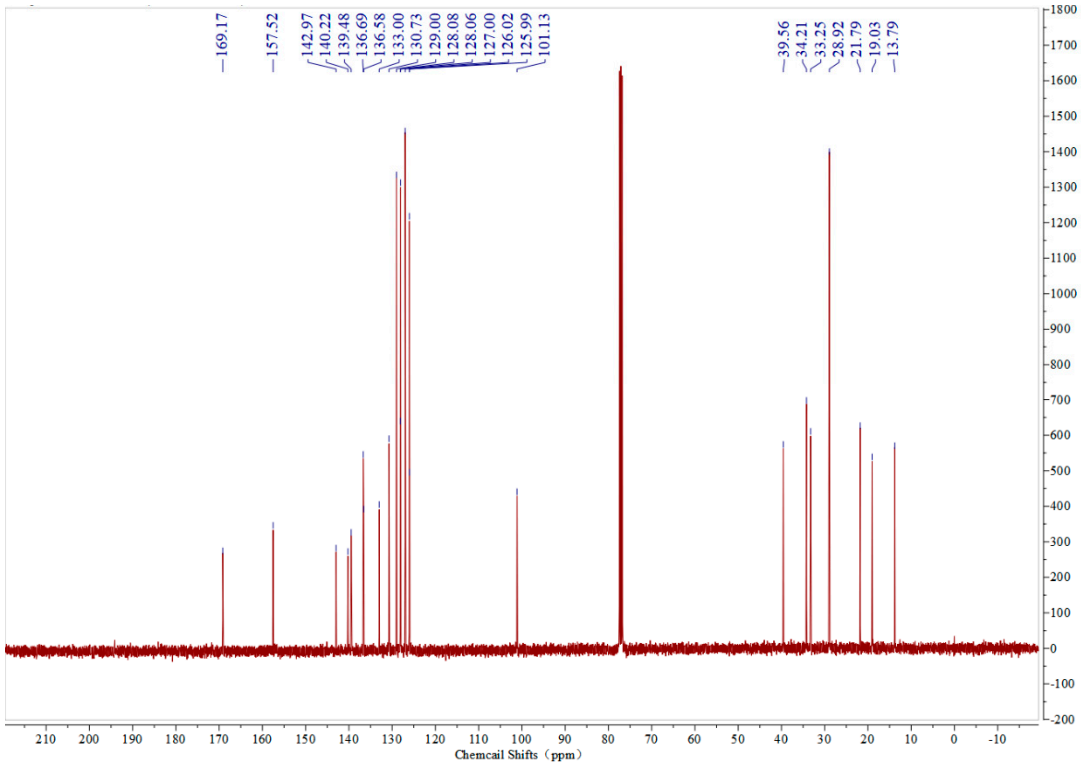

Figure S90. The ^{13}C NMR spectra of Compound 1v

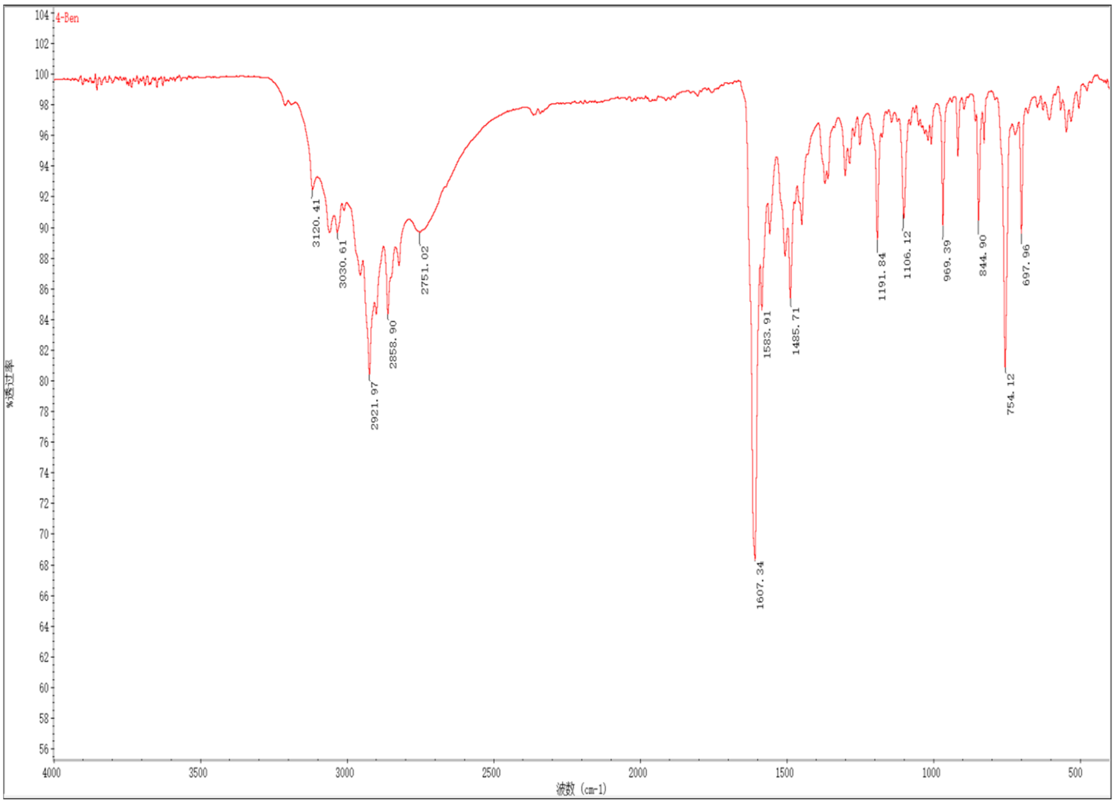

Figure S91. The FT-IR spectra of Compound 1v

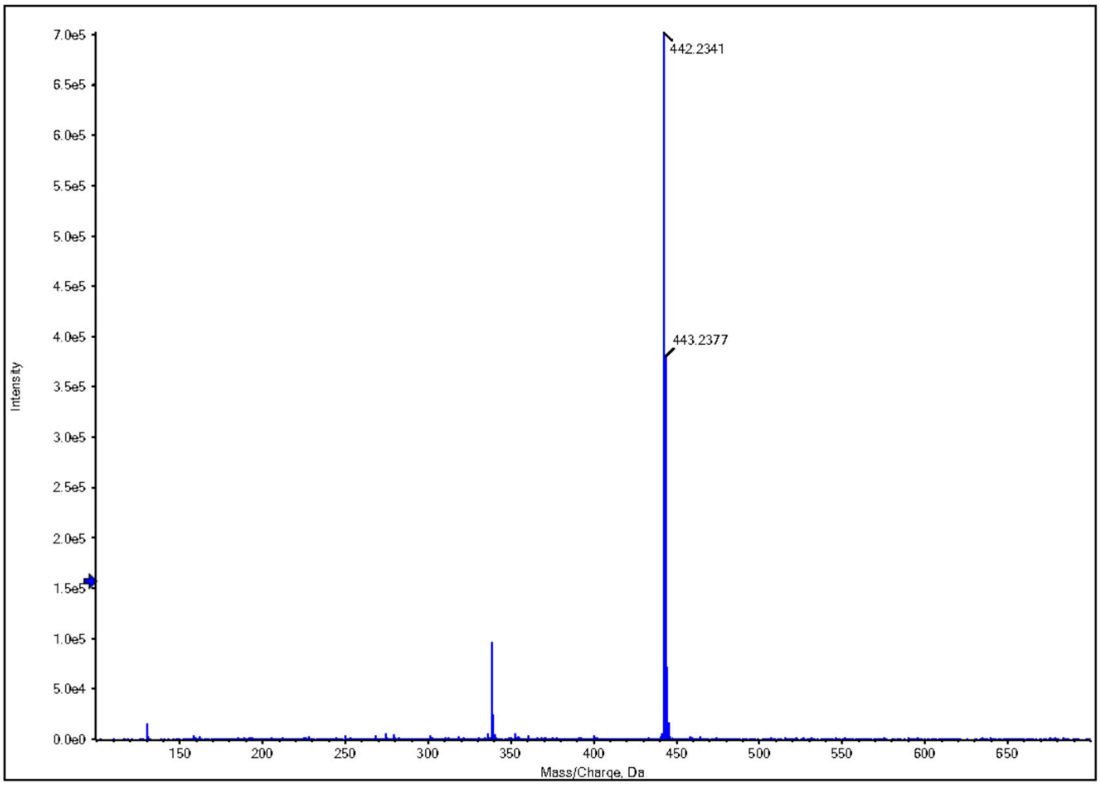

Figure S92. The HRMS spectra of Compound 1v

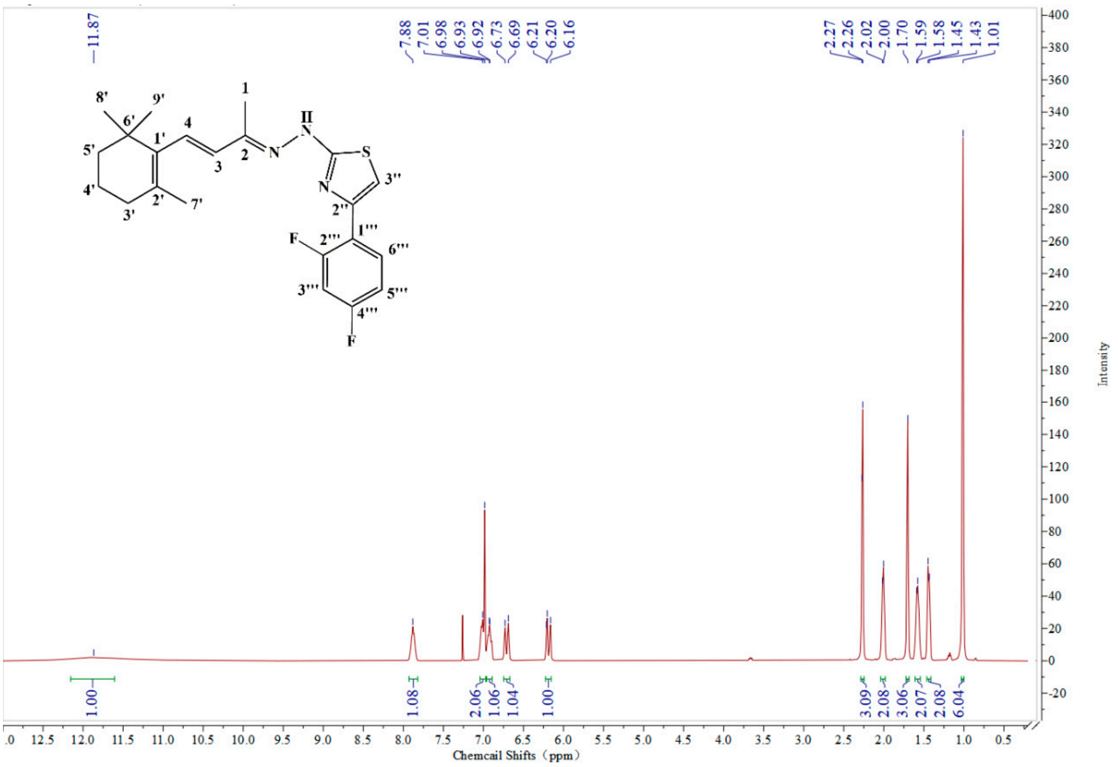

Figure S93. The ^{1}H NMR spectra Compound 1w

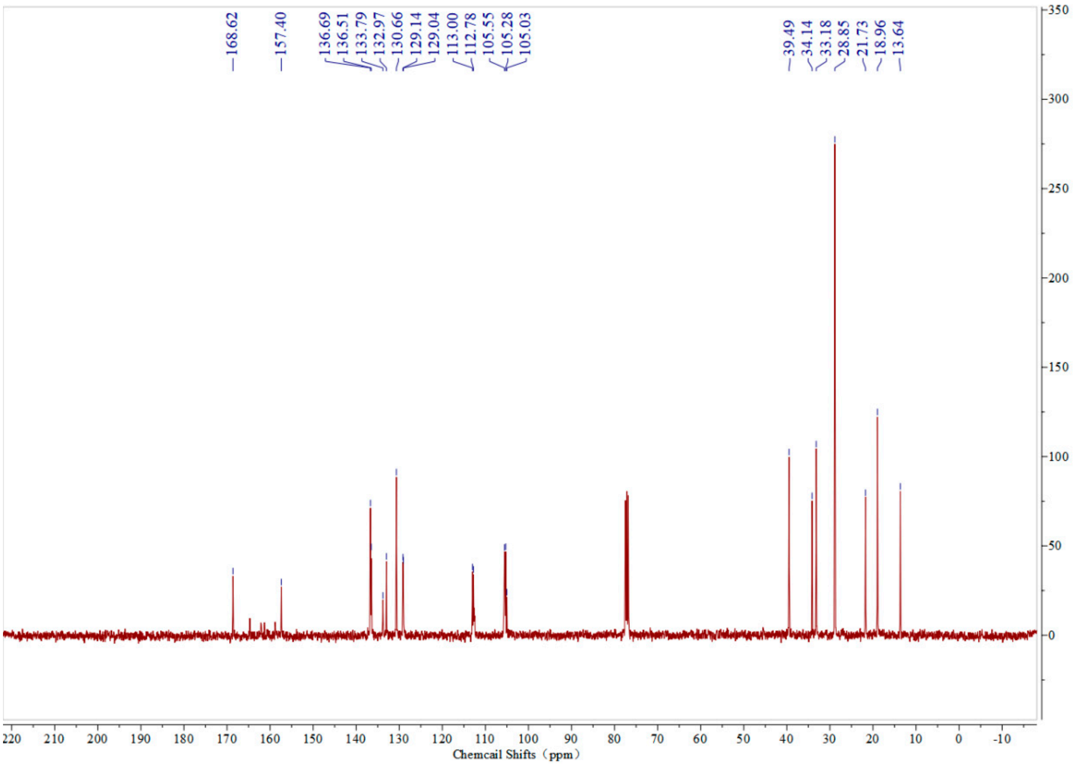

Figure S94. The ^{13}C NMR spectra of Compound 1w

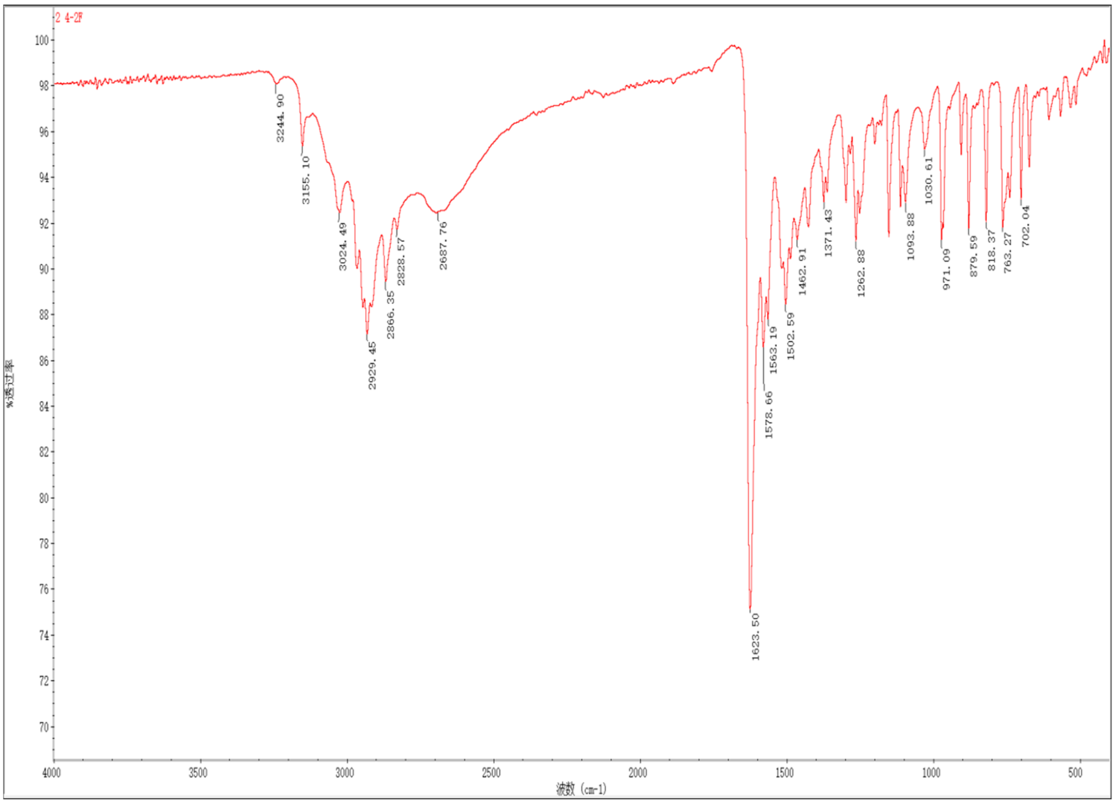

Figure S95. The FT-IR spectra of Compound 1w

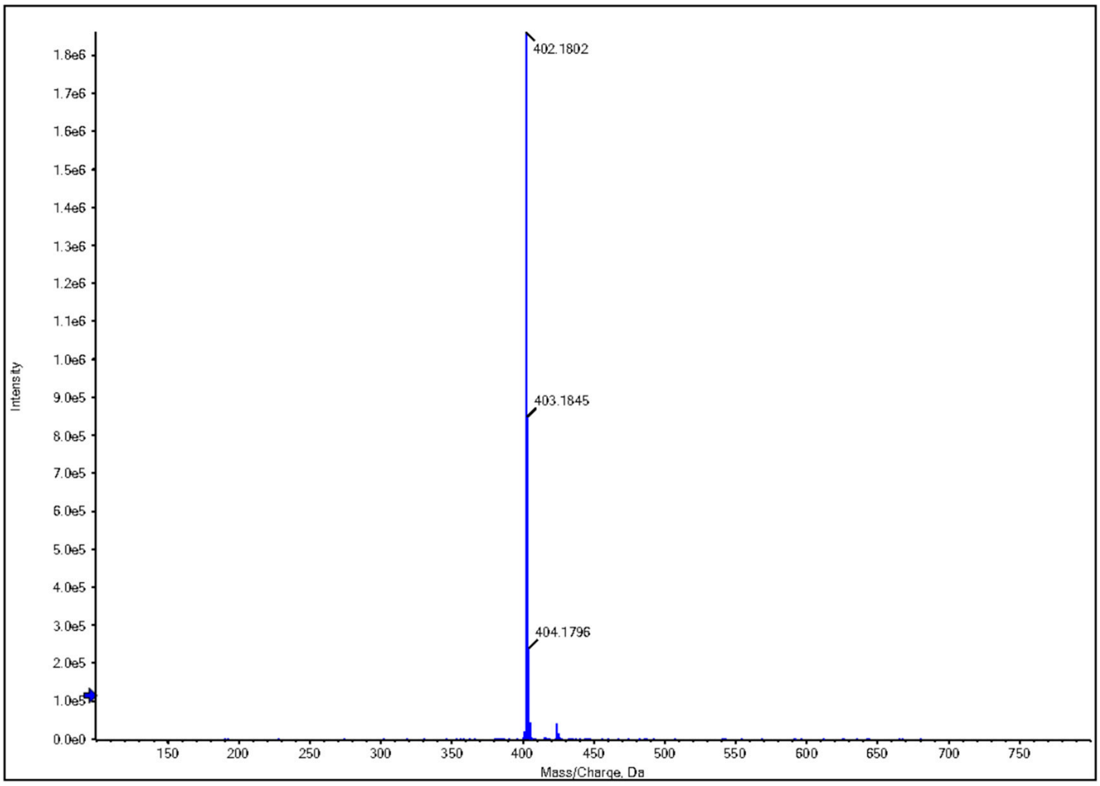

Figure S96. The HRMS spectra of Compound 1w

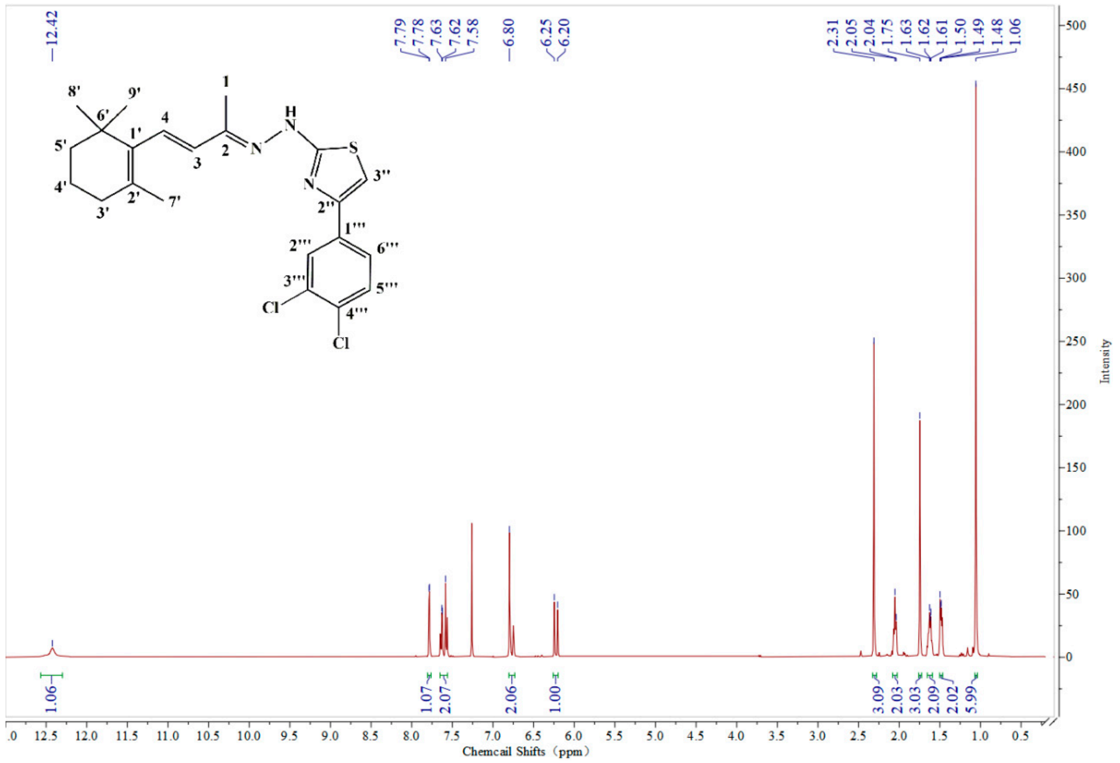

Figure S97. The ^{1}H NMR spectra Compound 1x

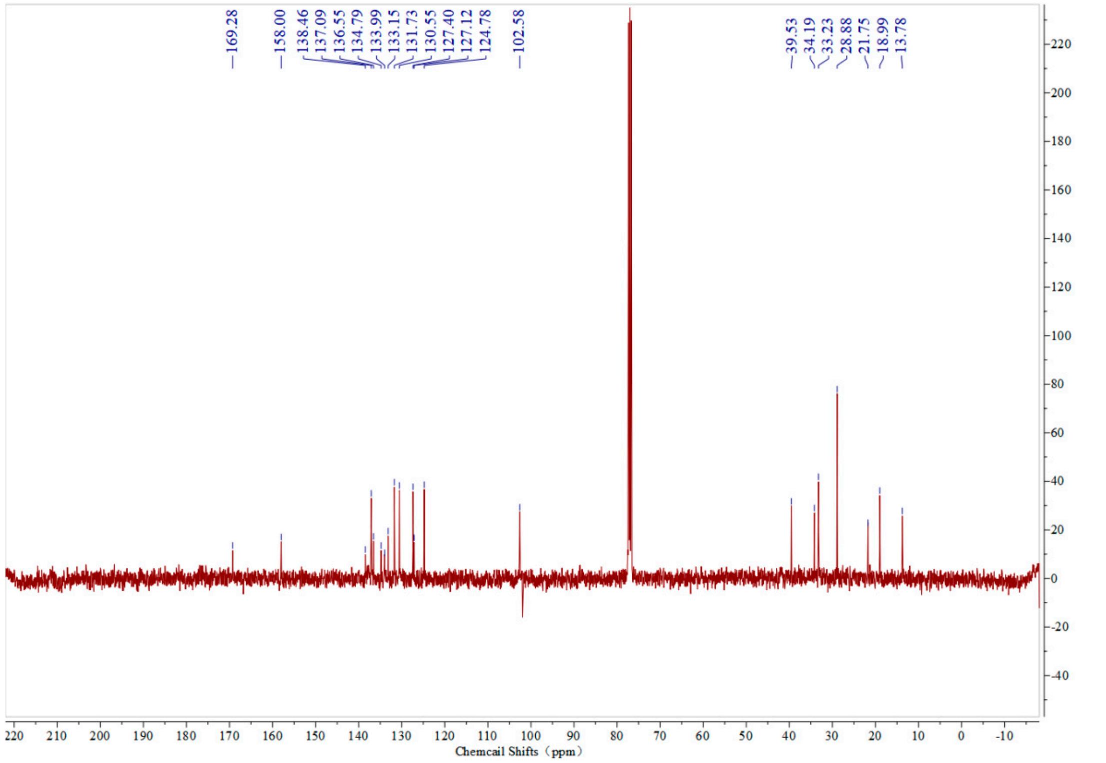

Figure S98. The ^{13}C NMR spectra of Compound 1x

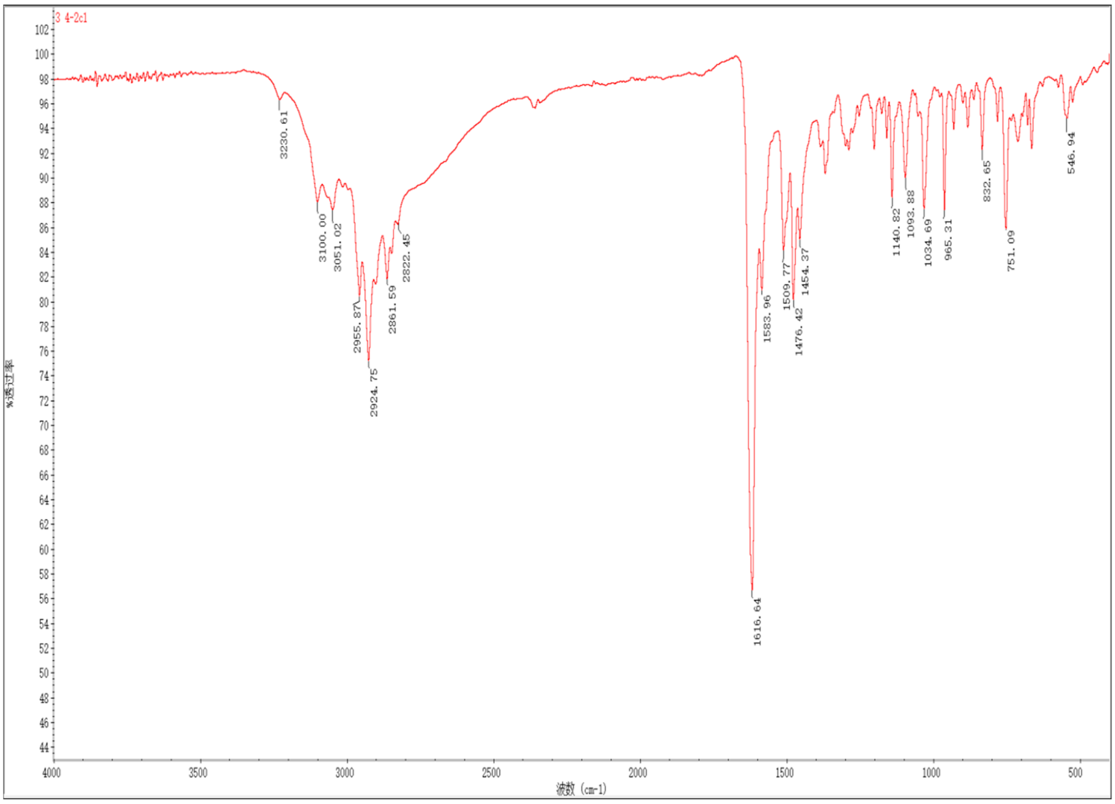

Figure S99. The FT-IR spectra of Compound 1x

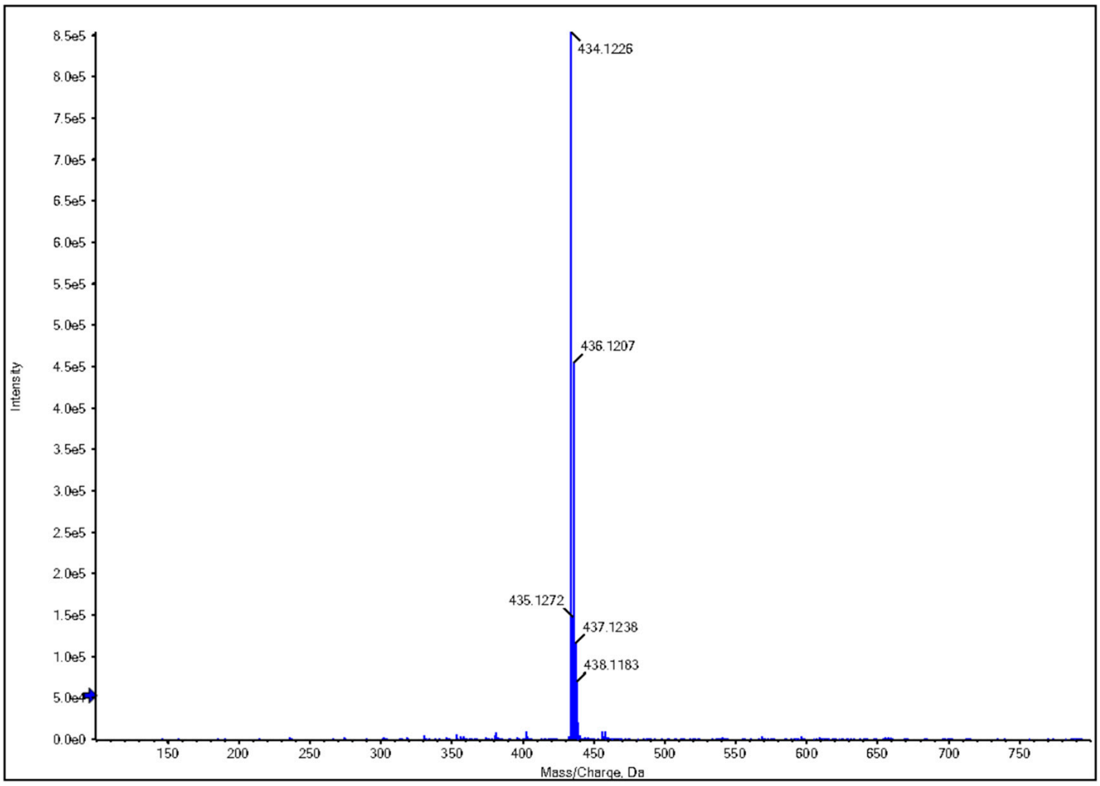

Figure S100. The HRMS spectra of Compound 1x

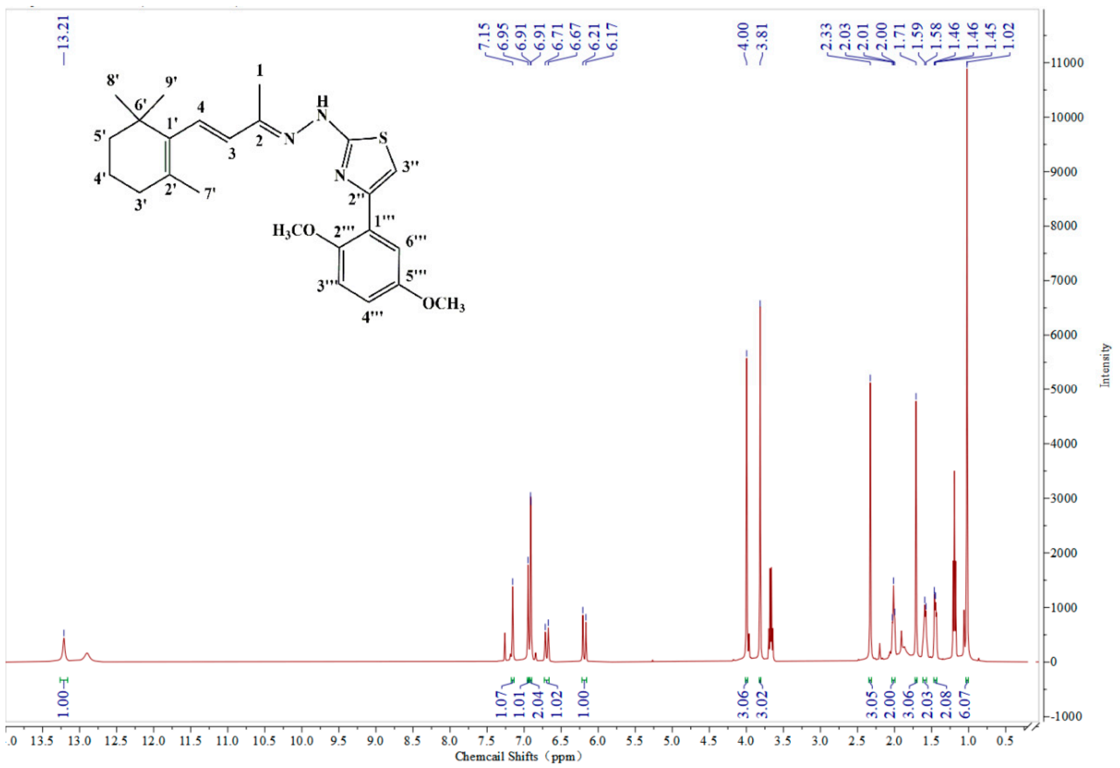

Figure S101. The ^{1}H NMR spectra Compound 1y

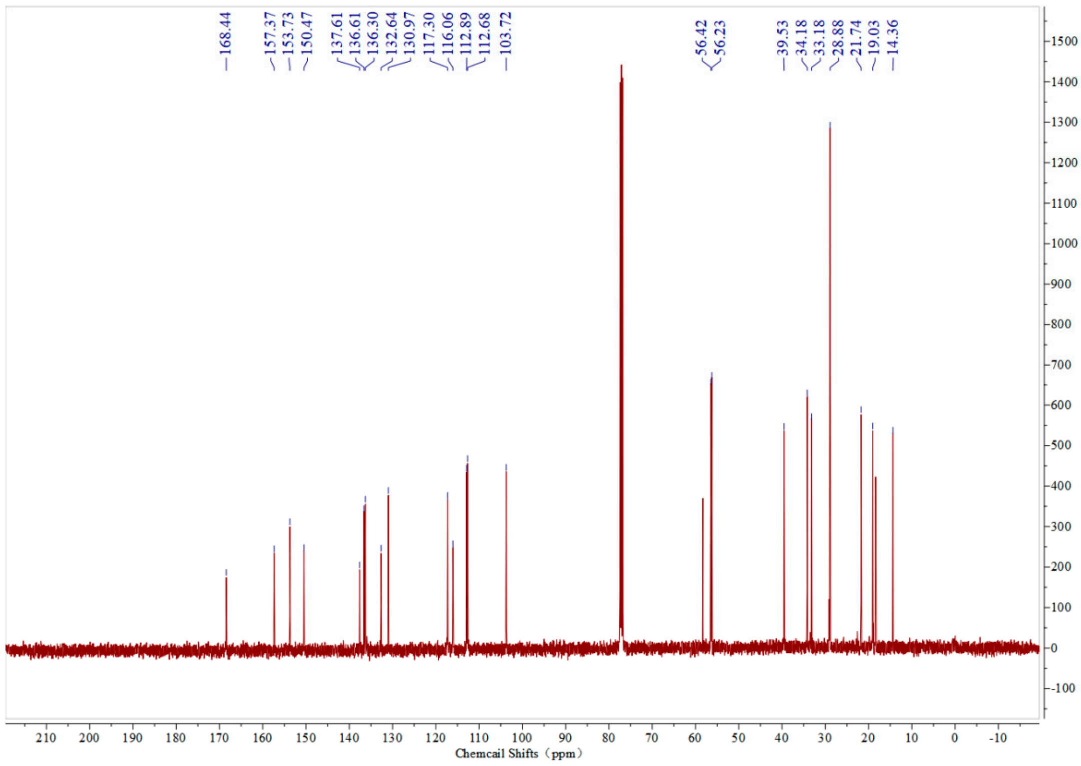

Figure S102. The ^{13}C NMR spectra of Compound 1y

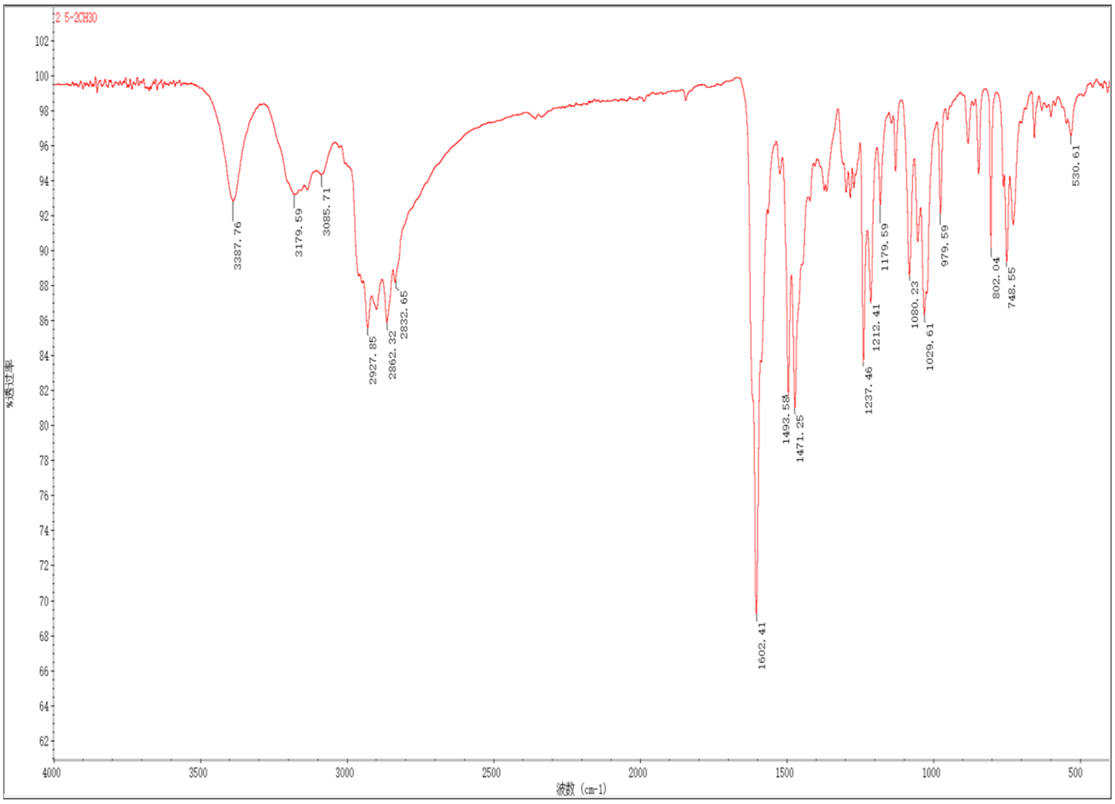

Figure S103. The FT-IR spectra of Compound 1y

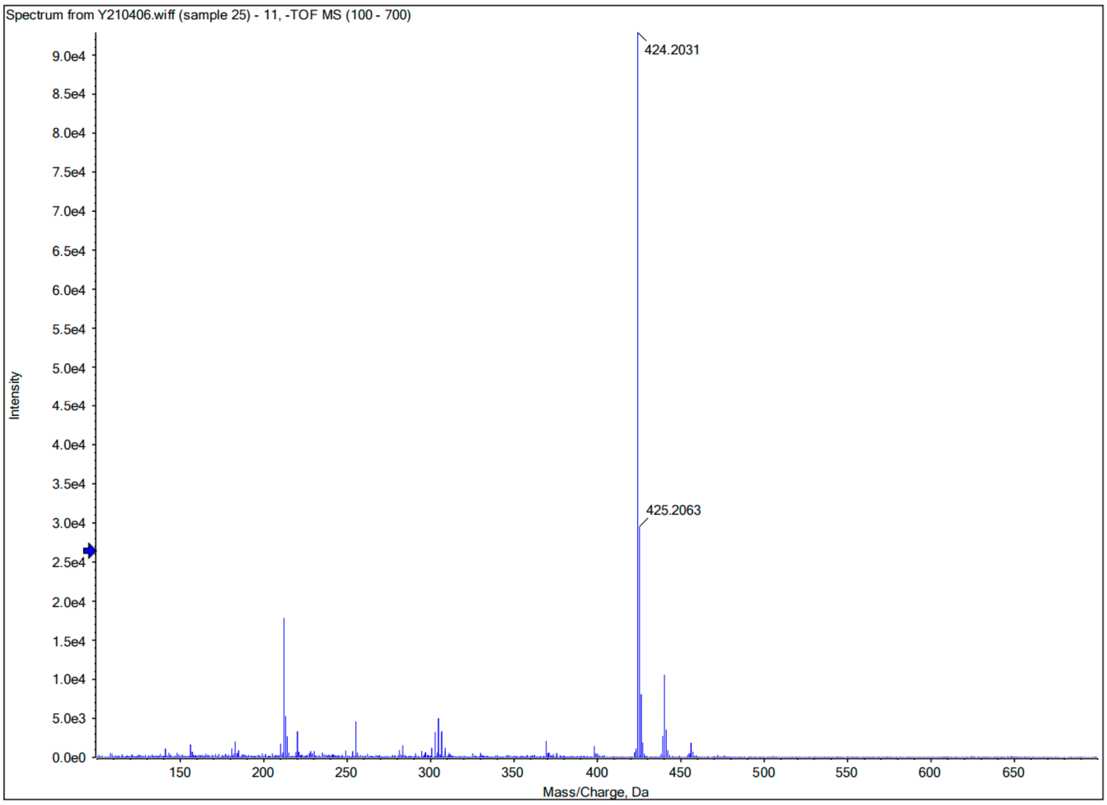

Figure S104. The HRMS spectra of Compound 1y
